# Networked salt-bridges mediate magnesium-dependent conformational dynamics and functional regulation in type IA topoisomerases

**DOI:** 10.1038/s41467-026-72556-9

**Published:** 2026-04-30

**Authors:** Yeonee Seol, Yuk-Ching Tse-Dinh, Keir C. Neuman

**Affiliations:** 1 Laboratory of Single Molecule Biophysics, National Heart, Lung, and Blood Institute, National Institutes of Health, Bethesda, MD USA; 2 Biomolecular Sciences Institute, Department of Chemistry & Biochemistry, Florida International University, Miami, FL USA

**Keywords:** Single-molecule biophysics, Enzyme mechanisms, DNA metabolism

## Abstract

Protein conformational dynamics are fundamental to enzyme function, yet the molecular mechanisms by which these dynamics are regulated remain poorly understood. Here, we reveal that a conserved network of salt-bridges, modulated by magnesium ions, serves as a key regulator of conformational transitions in Type IA topoisomerases (TopIA). Using a combination of single-molecule and ensemble measurements, molecular dynamics simulations, and targeted protein mutagenesis, we demonstrate that Mg²⁺ binding to a distinct divalent metal binding site orchestrates the opening and closing of the protein-mediated DNA gate—a critical step in TopIA’s catalytic cycle. Our results show that magnesium tunes the kinetics of the salt-bridge network’s configurational switching, directly impacting enzyme activity and providing a safeguard against DNA damage under Mg²⁺ depletion. This work provides a chemical and structural framework for understanding divalent cation-dependent regulation of protein function via networked salt-bridges. Our findings open additional avenues for the rational design of cation-sensitive proteins and inhibitors, and highlight an evolutionarily conserved strategy for coupling environmental sensing to molecular function.

## Abstract (web summary)

Here the authors reveal how magnesium regulates the conformational dynamics and activity of Type IA topoisomerases by reconfiguring salt-bridge networks in the protein. This simple, conserved mechanism may explain magnesium regulation of other proteins.

## Introduction

Type IA topoisomerases (TopIA) are essential and ubiquitous enzymes found across all domains of life^1^. TopIA is the first class of topoisomerases that was discovered in 1971^2^. The landmark discovery of ω protein—later identified as *E. coli* topoisomerase 1 (*ec*topo1)—resolved the longstanding mystery of how cells overcome topological constraints inherent to the double helical structure of DNA^3^. In addition to topoisomerase 1, which is largely involved in regulating and suppressing hyper-negative supercoiling, *E. coli* encodes a second TopIA, topoisomerase 3 (*ec*topo3), which functions in chromosome segregation and the resolution of replication intermediates and double holiday junctions via interactions with RecQ helicase^1^. Notably, topoisomerase 3 is the only type IA topoisomerase present in Eukaryotes^1^.

**Fig. 1:**
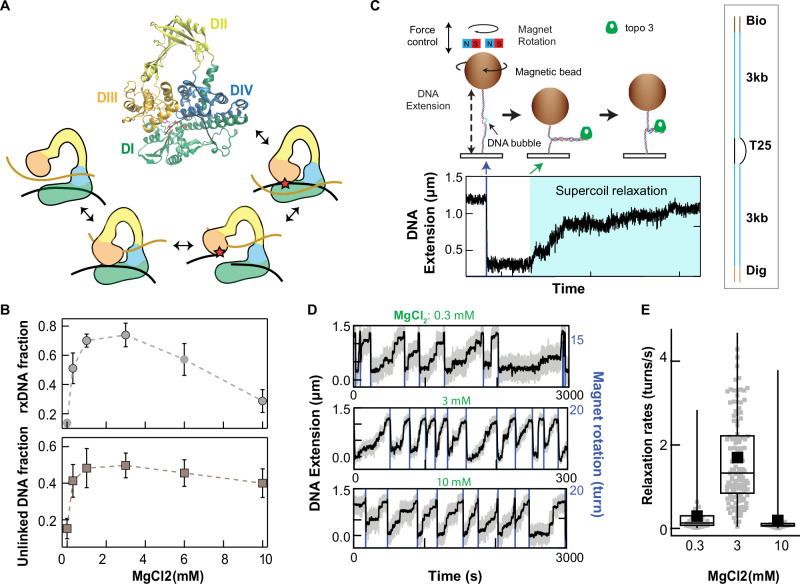
E. coli topoisomerase 3 (ectopo3) catalytic cycle and Mg-dependent activities. **A** Structure of ectopo3 (PDB 20I9) with a single-stranded DNA bound across domains I and IV. Individual domains are color-coded. Schematic of the topoisomerase IA catalytic cycle in which a protein-mediated DNA gate permits passage of the transfer strand (gold) through the gate strand (black) that has been cleaved by the enzyme (red star). **B** Negatively supercoiled DNA relaxation and kinetoplast DNA (kDNA) unlinking by ectopo3 as a function of magnesium (Mg) concentration (Supplementary Fig. 1). Fraction of relaxed (rxDNA) supercoiled DNA (scDNA) (top) and fraction of unlinked kDNA (bottom) as a function of Mg concentration. Quantification of gel electrophoresis results from 3 scDNA relaxation (3 biological replicates) and 5 kDNA unlinking (3 biological replicates) gels, with error bars corresponding to the standard error of the mean (SEM). **C** Cartoon of magnetic-tweezers DNA supercoil relaxation measurement. DNA was supercoiled by rotating the tethered magnetic bead with a magnet assembly above the sample chamber. Plectoneme formation leads to a reduction of DNA extension. Supercoil relaxation by ectopo3 results in an increase in DNA extension (blue shading in the trace). Right: DNA substrate containing an asymmetric DNA bubble to accommodate ectopo3. **D** Typical ectopo3 supercoil relaxation traces at three different Mg concentrations (0.3, 3, and 10 mM), note the 10-fold change in the time duration of the different traces. Once DNA supercoils were relaxed by the enzyme, the indicated number of magnet rotations (15 or 20) (right axis) was applied to re-supercoil the DNA. **E** Supercoil relaxation rates obtained from single-molecule relaxation trajectories at three Mg concentrations. Number of events (number of biological replicates): 38 (3) for 0.3 mM; 128 (3) for 3 mM; 35 (3) for 10 mM. Black solid squares indicate the mean of individual rates overlayed in the box plots. Error bars correspond to SEM. Data are presented as box plots showing the median, the 25th–75th percentiles, and the minimum and maximum values.

All TopIA share a similar toroidal core structure, comprising four domains that form a central cavity (Fig. 1A)^4^. The interface between domains I and IV forms a single-stranded DNA (ssDNA) binding groove, whereas domains II and III constitute an arch and a gate respectively^4–6^. During catalysis, a transient single-stranded break is introduced into the phosphate backbone of the bound single-stranded DNA by a transesterification reaction, mediated by a catalytic tyrosine in domain III (Fig. 1A)^4,7^. The resulting protein-DNA linkage serves as a ‶gate″, permitting a second DNA strand to enter into or out of the protein cavity— essential for unlinking catenanes or relaxing supercoiled DNA^4^. This proposed mechanism requires a large conformational change to open the protein-DNA gate sufficiently to accommodate a single or duplex DNA strand, raising the question of how such substantial structural rearrangements are achieved and regulated^8,9^. Whereas indirect evidence long supported the gate model^8,10,11^, recent single-molecule measurements provided direct visualization of a ~ 6-nanometer protein-DNA gate motion, confirming the conformational changes postulated by the gate model. These findings have also reinvigorated the debate over the roles of magnesium in TopIA activity and conformational regulation^12–16^.

Magnesium is the most abundant divalent metal in cells and is critical for numerous cellular processes, including cell cycle progression, cell proliferation, and immune response^17^. It often acts as a required co-factor for enzymatic activity and is essential for nucleic acid structure and function^18–22^. Accordingly, most DNA processing enzymes—including topoisomerases—feature a highly-conserved divalent metal binding motif to bind magnesium as a catalytic cofactor^14,20,23^. In both type IA and IIA topoisomerases (TopIIA), Mg^2+^ binding within the conserved topoisomerase-primase (TOPRIM) domain is essential for DNA transphosphorylation; absence of Mg²⁺ or mutations within this domain result in partial or complete loss of catalytic activity^24–26^. Whereas TopIIA absolutely require divalent metal ions for both DNA cleavage and religation, TopIA require divalent metal ions only for DNA religation; DNA cleavage by *ec*topo1, for example, can proceed without Mg^2+^^26–28^^,^. However, Mg²⁺-independent DNA cleavage by TopIA remains controversial. A recent study monitoring protein-DNA gate opening in multiple TopIA enzymes concluded that a lack of detectable gate opening in the absence of Mg²⁺ suggests a magnesium requirement for DNA cleavage, since cleavage precedes gate opening^12^. This apparent contradiction—that TopIA can cleave DNA without Mg^2+^, yet cannot open the enzyme-mediated DNA gate—suggests an unanticipated role for magnesium in regulating protein-gate motion, beyond its established catalytic function. Magnesium has been implicated in domain conformational changes, including those of the gate domain in *E. coli* topoisomerase 1^14,16,24^; however, there has been neither a direct observation nor a mechanistic basis established for magnesium-dependent protein-gate motion and subsequent enzymatic activity.

To elucidate the multifaceted roles of Mg^2+^ in TopIA chemistry and conformation dynamics, here we employ a combination of single-molecule and ensemble activity measurements, single-molecule gate dynamics measurements, and molecular dynamics simulations of *E. coli* topoisomerase IA. Whereas corroborating measurements and simulations were performed with *ec*topo1 to extend the results obtained with *ec*topo3 and for the sake of comparison with previous studies^12,16^, our current work is primarily focused on *ec*topo3 as it is a minimal topo IA consisting of a toroidal core without additional extended domains or required complex formation with other proteins. Our results confirm that Mg^2+^ is dispensable for DNA cleavage, but required for protein-mediated DNA gate opening. Specifically, we find that TopIA gate dynamics depend non-monotonically on Mg^2+^ concentration in a manner that mirrors the Mg^2+^ concentration dependence of TopIA relaxation and decatenation activity, thus underscoring the tight coupling between gate dynamics and enzyme catalysis. Molecular dynamic simulations reveal two competing salt-bridge configurations within domains II and III, each correlated with distinct gate opening pathways and kinetics. We identified a putative Mg²⁺ binding motif overlapping this salt-bridge network, suggesting a model in which transient Mg²⁺ binding catalyzes the switch between these configurations, thereby regulating gate opening and closing. Mutational disruption of the salt-bridge network altered Mg²⁺-dependent gate dynamics and catalytic activity, supporting these predictions. Together, our findings—and the conservation of overlapping Mg^2+^-binding and salt-bridge topologies among TopIA enzymes—suggest a general mechanistic framework for divalent metal ion regulation of protein conformations and dynamics.

## Results

### Mg^2+^-dependent enzymatic activities of *ec*topo3

Although Mg^2+^-dependent catalytic activity of *ec*topo1 has been extensively characterized^23,24,29,30^, comparatively little is known about *ec*topo3. We found that, similar to *ec*topo1^24,29^, *ec*topo3 exhibits a non-monotonic Mg^2+^-dependent enzymatic activity (Fig. 1B–E, Supplementary Fig. 1). Both DNA unlinking and supercoil relaxation activities exhibit similar dependence on Mg^2+^ concentration. The activities increased (Fig. 1B) above 0 mM, likely reflecting an increase in ssDNA transphosphorylation activity (Supplementary Fig. 2A). However, unlinking and relaxation activities decreased gradually from 3 mM to 10 mM, despite a slight increase in cleavage and religation rates (Supplementary Fig. 2B). This reduction was not due to changes in single-stranded DNA binding affinity, which was relatively insensitive to Mg^2+^ concentration (Supplementary Fig. 2C). The decreased activity at higher Mg^2+^ concentrations may reflect increased DNA duplex stability^31,32^, reducing local melting of negatively supercoiled DNA and thereby decreasing accessible single-stranded DNA regions required for *ec*topo3 binding. To circumvent this potential confounding effect, we performed single-molecule supercoil relaxation measurements using a bubble DNA substrate (Fig. 1C), which ensures enzyme binding independent of possible Mg^2+^ induced changes in ssDNA accessibility. We generated positive supercoils to ensure a single enzyme binding site and measured the relaxation activity at three Mg^2+^ concentrations (0.3, 3, and 10 mM) (Fig. 1D, E). Additionally, the chamber was washed multiple times to remove unbound proteins after introducing 100 pM of *ec*topo3, ensuring that the measured unlinking rate was independent of enzyme binding. Continuous relaxation events typically lasted for at least an hour. The average rates from single-molecule relaxation measurements showed a similar, but slightly more pronounced, Mg^2+^ concentration-dependence compared to ensemble measurements (Fig. 1E), confirming the inhibitory effects of high Mg^2+^ concentrations. These results suggest that the reduced activity at 10 mM Mg^2+^, despite cleavage and religation rates comparable to those at 3 mM (Supplementary Fig. 2B), could reflect an additional Mg^2+^-dependent rate-limiting step during the enzymatic cycle. One potential rate-limiting step is the passage of the transfer DNA segment through the protein-mediated DNA gate, a process that depends in part on gate dynamics. To directly probe the effects of Mg^2+^ on the protein-mediated DNA gate conformational dynamics, we measured the protein-DNA gate motion of *E. coli* TopIA at the single-molecule level using magnetic tweezers^33^.

### Mg^2+^ is required for opening of the protein-DNA gate and influences gate dynamics of both *E. coli* TopIA enzymes

**Fig. 2:**
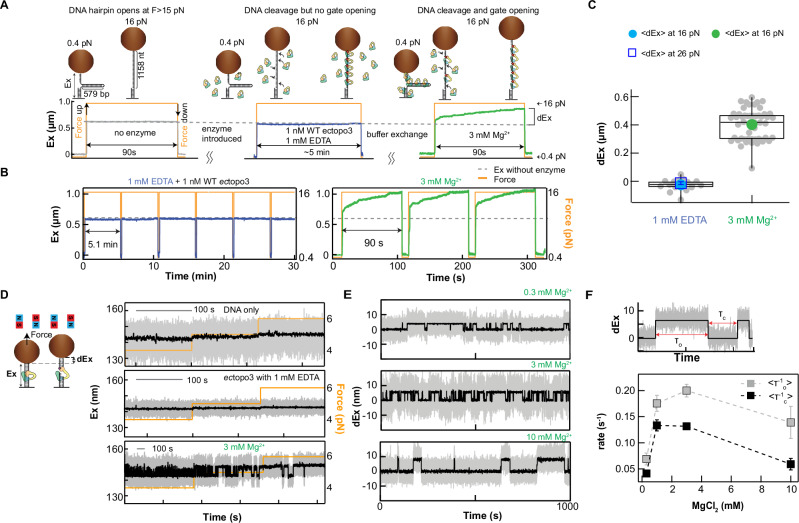
Magnesium-dependence of *ec*topo3 gate opening and dynamics. **A** Schematic of DNA hairpin-based gate opening measurements. The DNA hairpin was attached to the surface via digoxigenin-anti-digoxigenin bonds at one end and to a 2.8 µm super-paramagnetic bead via biotin-streptavidin bonds at the other. Force (orange line) on the bead was modulated from low to high during the measurements. At high force (16 pN), the hairpin opens, exposing ~ 1 kb of ssDNA to which multiple TopIA can bind, cleave, and extend through gate openings. Gate closing is inhibited at high force, simplifying analysis of the DNA extension (Ex) measurements. After a set duration at high force, the force was decreased (0.4 pN) to allow the hairpin to reform and to promote unbinding of TopIA from the ssDNA. Measurements were repeated for ~30 min with 1 mM EDTA (blue line) and for ~10 min with 3 mM Mg^2+^ (green line). **B** Example trace with 1 mM EDTA and 1 nM *ec*topo3 showed no change of DNA extension (blue line), indicating no gate-opening. After buffer exchange with protein-free buffer containing 3 mM Mg^2+^, the extension increased gradually (green line), indicating unsynchronized DNA binding, cleavage, and gate-opening of multiple enzymes. Force changes (orange line) are overlayed with the extension plots. **C** Box plots of extension change (dEx, gray points) and mean extension change at 1 mM EDTA (filled blue circle 16 pN, open blue circle 26 pN) and 3 mM Mg^2+^ (gray points and closed green circle 16 pN). Negative dEx values indicate hindering of hairpin opening by bound enzymes. Number of events (number of biological replicates): 19 (3) for 1 mM EDTA; 52 (3) for 3 mM Mg^2+^. Data are presented as box plots showing the median, the 25th–75th percentiles, and the minimum and maximum values. **D** Cartoon of single-molecule *ec*topo3 gate dynamics measurements (left). The DNA tether is kept under constant tension by external magnets that exert a force on the one-micron magnetic bead attached to the free end of the gapped DNA molecule. The DNA extension (Ex) is obtained by tracking the height of the bead at 200 Hz. Example traces of DNA only (no enzyme; top), DNA in the presence of 100 pM *ec*topo3 with 1 mM EDTA (middle), and the same DNA after flowing in protein-free 3 mM Mg^2+^ buffer (bottom). Orange line indicates 1 pN force increments from 4 to 6 pN. **E** *Ec*topo3 gate dynamics depend on Mg concentration. Examples of gate dynamics measurements at 0.3 mM (top), 3 mM (middle), and 10 mM Mg^2+^, at a force of 5 pN. **F** The durations of the open (τ_o_; gray solid squares) and closed (τ_c_; black solid squares) states obtained from the single-molecule trajectories measured at an equilibrium force of ~5 pN (top). Number of biological replicates: 3; 5; 5; 4 for 0.3, 1, 3, and 10 mM Mg, respectively. Error bars correspond to the standard deviation. Average closing (τ_o_^−1^) and opening (τ_c_^−1^) rates as a function of Mg^2+^ concentration obtained from the single-molecule trajectories (bottom). Number of events (number of biological replicates): 61 (3); 274 (5); 313 (5); 58 (4) for 0.3, 1, 3, and 10 mM Mg, respectively. Error bars correspond to the standard deviation.

To determine the requirement of Mg^2+^ for protein-DNA gate opening, we monitored the extension change of a 579 bp DNA hairpin under high force (> 16 pN), which exposes 1158 nucleotides of single-stranded DNA for multiple TopIA to bind (up to ~83 based on the reported 14 nt footprint of *ec*topo3 on ssDNA)^34,35^, and favors the protein-DNA gate to remain open (Fig. 2A), as previously established^15^. In agreement with previous results^12^, neither *E. coli* TopIA enzyme exhibited gate opening in 1 mM EDTA (Mg^2+^ depletion), as no discernible extension increase was observed compared to the case of no enzyme (dashed line) (Fig. 2A, B, Supplementary Fig. 3). DNA binding and catalytic competence of TopIA enzymes were confirmed by the robust gate opening of the remaining enzymes after buffer-exchange with 3 mM Mg^2+^ protein-free buffer. The ~400 nm extension increase over 90 s reflects DNA binding and gate opening of ~57 enzymes based on the previously estimated 7 nm gate opening measured under similar conditions (Fig. 2A–C). We note that the buffer exchange reduced the free enzyme concentration in the sample chamber. The fact that robust ssDNA extension increase was observed only after buffer exchange, despite the associated reduction in enzyme concentration, strengthens the finding that Mg^2+^ is required for gate opening, as no gate opening was observed at higher concentrations of enzyme in the absence of Mg^2+^. In parallel, DNA cleavage activity was verified under identical Mg^2+^ depletion conditions (Supplementary Fig. 4). Gate opening was not observed with high concentrations of monovalent salts (300 mM potassium) in the absence of magnesium (Supplementary Fig. 3C–F), suggesting that TopIA gate opening is specifically facilitated by Mg²⁺ rather than through nonspecific electrostatic screening.

To further probe Mg^2+^-dependence of protein-gate conformational changes, we measured gate dynamics over a range of Mg^2+^concentrations using a duplex DNA substrate containing a short single-stranded gapped DNA (Fig. 2D). As described previously^15^, transitions between open and closed gate states are detected under moderate tension (4–6 pN). Consistent with the absence of gate opening under Mg^2+^ depletion (Fig. 2B, C), no extension fluctuations indicating gate dynamics were observed for either *ec*topo1 or *ec*topo3 in 1 mM EDTA (Fig. 2D, Supplementary Fig. 5). Upon introduction of protein-free Mg^2+^ containing buffer, gate dynamics commenced (Fig. 2D, Supplementary Fig. 5), confirming that the enzyme was bound, but did not undergo gate dynamics in the absence of Mg^2+^. We next examined the sensitivity of *ec*topo3 gate dynamics to Mg^2+^ concentration (Fig. 2E). At 0.3 mM Mg^2+^, the extent of gate opening was similar to that at 3 mM, but the opening and closing rates were slower (Fig. 2E). This reduction in both opening and closing rates at a suboptimal Mg^2+^ concentration cannot be explained by slower DNA cleavage, suggesting that Mg^2+^ modulates both transitions. Surprisingly, gate dynamics were also reduced at 10 mM Mg^2+^, indicating a biphasic dependence with maximal rates near 1 mM (Fig. 2E, F).

This Mg^2+^-dependence closely matches the observed non-monatomic Mg^2+^-dependent relaxation and decatenation activities (Fig. 1), suggesting that gate dynamics largely determine enzymatic activity. Overall, these results establish that Mg^2+^ is indispensable not only for gate opening but, more importantly, for optimal gate conformational dynamics. To probe the specificity of Mg^2+^ effects on gate dynamics, we replaced Mg^2+^ with divalent metal ions that can support enzymatic activity; manganese (Mn^2+^) and Calcium (Ca^2+^)^23^. No gate dynamics was observed with *ec*topo3 in the presence of Ca^2+^, despite robust ssDNA cleavage (Supplementary Fig. 6). Consistent with the absence of gate dynamics, *ec*topo3 supercoil relaxation activity was inefficient with Ca^2+^, underscoring the coupling between gate dynamics and enzymatic activity (Supplementary Fig. 6). Whereas Mn^2+^ supported *ec*topo3 gate dynamics, the dynamics were strikingly different from those with Mg^2+^ (Supplementary Fig. 6). Furthermore, the absence of gate dynamics at 75 µM; a 7.5-fold higher concentration of Mn^2+^ than estimated in vivo (~10 µM)^36^ suggests that is unlikely that Mn^2+^ would replace Mg^2+^ in vivo. Overall, the observation of Mg^2+^-dependent and specific protein conformational dynamics is intriguing and begs the question as to the mechanistic basis.

### Overlapping Mg binding site and salt-bridge network suggest mechanistic basis for Mg-dependent gate dynamics

Although canonical Mg^2+^ binding sites are limited to the conserved TOPRIM domain, our findings suggest additional sites may exist. Using the BioMetAll metal binding prediction algorithm, which was shown to efficiently predict noncanonical and transient metal binding sites^37^, we identified several putative metal binding sites in both *ec*TopIA enzymes including non-TOPRIM sites (Supplementary Fig. 7 and 8). Notably, the highest-probability site in *ec*topo3 lies within domain II, comprising three glutamic acidic residues (E454, E457, and E458; Supplementary Fig. 7A). Since BioMetall does not distinguish among different divalent metal types, we specifically tested the possibility of magnesium binding at this additional site, as well as at TOPRIM, using the automated ligand docking program AutoDock (Supplementary Information). Consistent with the BioMetall predictions, AutoDock also identified reasonably stable Mg²⁺ interactions clustered around both predicted divalent metal binding sites (Supplementary Fig. 7B, C)^38^.

**Fig. 3:**
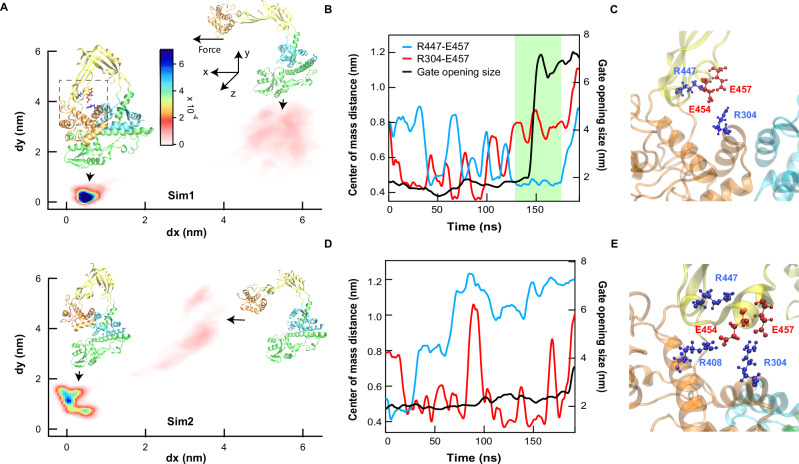
MD simulations reveal two distinct *ec*topo3 salt-bridge topologies that differentially influence gate opening. **A** 2-dimensional trajectories of two representative simulations under constant force (50 pN). Displacement of the catalytic tyrosine orthogonal to the direction of applied force (dy) and in the direction of applied force (dx). Corresponding structures of the closed and open configurations from MD simulations are shown. Top. sim1: trajectory showing a rapid two-state gate opening transition. Bottom. Sim2: trajectory showing an initial orthogonal movement to a closed intermediate state, followed by slower and incomplete gate opening compared to Sim1. The boxed area (gray dashed line) indicates the region of interest enlarged in C and E. **B** Gate opening distance (black solid line) and the center of mass distances between residues E457 and R304 (red line) and E457 and R447 (blue line) from sim 1 are plotted as a function of simulation time. Gate opening occurs during the prolonged E457-R447 salt-bridge formation (green shaded region). **C** MD simulation snapshot from sim1 illustrating the “intradomain” salt bridge configuration that promotes gate opening, in which both E454 and E457 are in close proximity to R447, not R304. **D** Gate opening distance (black solid line) and the center of mass distances between E457 and R304 (red line) and E457 and R447 (blue line) from sim 2 are plotted as a function of simulation time. The interdomain salt-bridge (E457-R304) persists, whereas the intradomain salt-bridge pair (E457-R447) remains separated, and the gate remains closed for the duration of the simulation (black solid line). **E** MD simulation snapshot from sim 2 illustrates the “interdomain” salt-bridge formation that promotes gate-closing, in which E454 and E457 in domain II are in close proximity to R408 and R304 in domain III, respectively, whereas R447 is pointed away from E457.

Strikingly, two of these residues, E454 and E457, were previously identified as components of an interdomain salt-bridge network implicated in regulating gate domain motion^39^. Salt-bridges are a form of electrostatic interaction between two oppositely charged amino acid groups in proteins that play critical roles in protein stability in addition to conformational specificity and flexibility^40,41^. Though often depicted as static, salt-bridges can be transient and interchangeable among clustered charged amino acid residues depending on the local environment^40,41^. In *ec*topo3, persistent interactions among two glutamic acids, E454 and E457 in domain II (DII) with lysine 303 (K303), arginine 304 (R304), and arginine 408 (R408) in domain III (DIII) were suggested to influence gate opening, revealing a potential role of salt-bridges in regulating protein conformation^39^. Extending these results and investigating the role of dynamic salt-bridge interactions in the gate opening transition, we performed steered molecular dynamics (SMD) simulations under constant force (50 pN)^42^, mimicking single-molecule gate-opening assays (“Methods”). Although simulations at such high-forces are not ideal for obtaining experimentally relevant kinetics or the exact energy landscape, they can still reveal the qualitative structural mechanisms, identify accessible intermediates and distinct conformational pathways^42^. Additionally, they can provide valuable molecular insights into how the residues of interest influence the gate-opening transition. The SMD results revealed that gate opening proceeds via two distinct conformational pathways under identical simulation conditions: one (sim1) opens via a two-state transition (Fig. 3A), and the other (sim2) opens via an intermediate state where domain III initially moves orthogonally to the applied force (*x*-direction) before transitioning to an open state (Fig. 3A). These pathways correlated with different intra- and interdomain salt-bridge configurations. Specifically, in sim1 domain III movement in the *x*-direction is hindered by the interdomain E457-R304 bridge until E457 forms an intradomain bridge with R447, facilitating opening (Fig. 3B, C). Conversely, persistent interdomain E454-R408 and E457-R304 bridges in sim 2 impede DIII movement in the *x*-direction and consequently gate opening (Fig. 3D, E).

We note that despite the presence of Mg^2+^ in the simulations, Mg^2+^ binding to the predicted residues was not observed as the SMD was not configured to model Mg^2+^–protein-residue interactions. However, considering the overlapping Mg^2+^ binding residues and the two competing salt-bridge network topologies, we propose that Mg^2+^ binding at the acidic triad (E454, E457, and E458) could transiently destabilize these salt-bridges, promoting configurational switching between intra- and inter domain salt-bridge interactions and thereby regulating gate conformations and dynamics. This model explains the lack of full gate opening in the absence of Mg^2+^ and slow gate dynamics at low Mg^2+^ concentrations (Fig. 2E, F). Similar dual competing salt-bridge configurations and an overlapping predicted metal binding site were found in *ec*topo1 (Supplementary Fig. 8), suggesting that Mg-dependent salt-bridge dynamics underlie *ec*topo1 gate dynamics in an analogous manner as *ec*topo3. Interestingly, the charged residues (442–447) that form the proposed salt-bridge network and the putative Mg²⁺ binding site in *ec*topo1 were previously suggested to be involved in regulating gate opening^34^. A recent study revealed that mutating these charged residues to glycines leads to reduced relaxation activity in vitro, increased cellular negative supercoiling, and transcriptomic changes reminiscent of topo1 depletion in vivo^43^.

### Mutagenesis of salt-bridge network components supports Mg-dependent protein conformational dynamics hypothesis

**Fig. 4:**
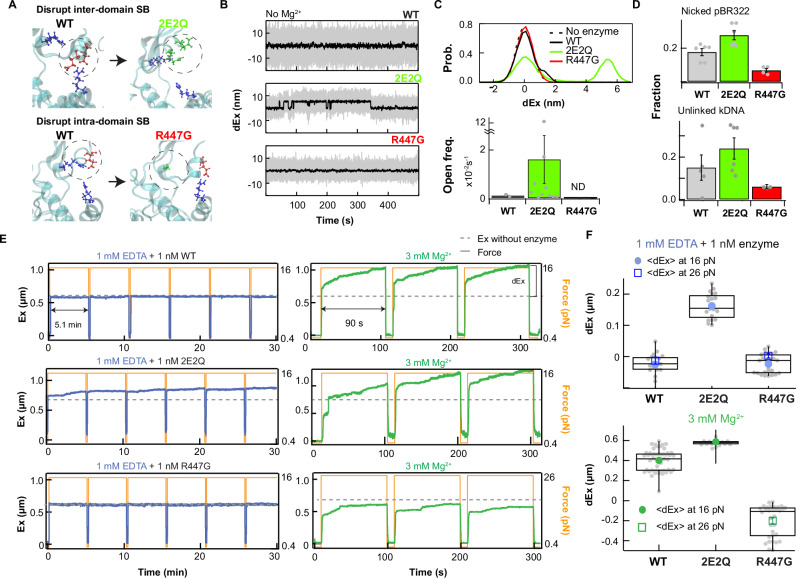
Two *ec*topo3 salt-bridge mutations confirm that Mg-dependent salt-bridge interactions control protein-gate dynamics. **A** Mutagenesis scheme to selectively disrupt salt-bridge interactions. **Top**. Mutations of E454 and E457 to Q454 and Q457 are predicted to prevent interdomain salt-bridge formation with R408 and R304 that promotes gate closing. **Bottom**. Mutation of R447 to G447 is predicted to abolish intradomain salt-bridge formation between R447 and E457 that facilitates gate opening. **B** Example gate dynamics trajectories for the three *ec*topo3 variants in the absence of added Mg^2+^. **C** Probability distributions of extension changes for DNA alone and in the presence of the three *ec*topo3 variants in the absence of Mg^2+^ (top) (WT: black, 2E2Q: green, R447G: red solid lines). Frequency of gate opening of the three *ec*topo3 variants (bottom) (WT: gray, 2E2Q: green, R447G: red solid bars; individual data points: gray circles). Number of biological replicates: 5 (WT); 10 (2E2Q); 5 (R447G). **D** Plasmid DNA nicking (top) and kinetoplast DNA unlinking activity (bottom) of three topo 3 variants in the absence of added Mg^2+^ (WT: gray, 2E2Q: green, R447G: red solid bars; individual data points: gray circles). Number of biological replicates of nicking: 6 (WT); 6 (2E2Q); 4 (R447G), and unlinking: 5 (WT); 6 (2E2Q); 3 (R447G). Error bars correspond to SEM. **E** Gate opening of 2E2Q is independent of Mg^2+^, in contrast to WT and R447G. Example traces of gate-opening measurements of WT, 2E2Q, and R447G on hairpin DNA substrate (“Methods”). The reduced extension of the hairpin relative to the fully opened hairpin (dashed line) in the presence of R447G with 3 mM Mg^2+^ likely results from enzyme binding to the duplex DNA, preventing full hairpin opening (negative dEx). Extension with 1 mM EDTA is plotted in blue and with 3 mM Mg^2+^ in green, while force is in orange. **F** Box plots of maximum extension change (dEx, gray points) for three enzymes with 1 mM EDTA (top) and with 3 mM Mg^2+^ (bottom). Mean extension changes at 1 mM EDTA are shown as filled blue circles (16 pN), open blue square (26 pN) and those at 3 mM Mg^2+^ as closed green circle (16 pN) and open green square (26 pN). Number of events (number of biological replicates): 19 (3) for WT; 20 (3) for 2E2Q; 25 (3) for R447G with 1 mM EDTA and (52) 3 for WT; 21 (3) for 2E2Q; (32) 3 for R447G at 3 mM Mg^2+^. Data are presented as box plots showing the median, the 25th–75th percentiles, and the minimum and maximum values.

To directly test Mg^2+^-dependent reorganization of the salt-bridge network in regulating gate dynamics, we engineered two *ec*topo3 mutants to specifically destabilize one or the other of the two competing salt-bridge configurations. 2E2Q is a double point mutant in which glutamic acids 454 and 457 were mutated to glutamine (Q) to disrupt interdomain salt-bridge interactions (Fig. 4A) and abolish the requirement of Mg^2+^ binding to facilitate gate opening. If, as we postulate, interdomain salt-bridge interactions hinder gate-opening, particularly in the absence of Mg^2+^, then we would expect to observe gate opening in 2E2Q, even in the absence of Mg^2+^. Conversely, R447G is a point mutant in which arginine 447 was mutated to glycine to disrupt its interaction with E457 or E454 (Fig. 4A). We postulate that the intradomain R447-E457 or R447-E454 salt-bridge promotes gate opening by competing with interdomain salt-bridge formation. Thus, disrupting this intradomain salt-bridge would stabilize the closed state and hinder gate opening. MD simulations support these expectations: 2E2Q exhibited increased probability of, and faster, gate opening, whereas R447G exhibited decreased probability of, and slower, gate-opening in comparison to WT *ec*topo3 (Supplementary Fig. 9). Additionally, the MD simulations revealed that the orthogonal fluctuations of the gate domain (along the *y*-axis) did not result solely from interdomain salt bridge interactions, but from the combined effects of the intra- and interdomain salt bridges as the orthogonal motion observed in the WT simulations diminished for both mutants, particularly R447G (Supplementary Fig. 9A).

Single-molecule gate dynamics and gate opening measurements of the two mutants supported the Mg^2+^-dependent salt-bridge disruption hypothesis (Fig. 4B–F). 2E2Q exhibited robust gate opening and dynamics in the absence of Mg^2+^ (Fig. 4B, C). Even under Mg^2+^ depletion (1 m EDTA), we observed gate opening in 2E2Q in stark contrast to the WT enzyme (Fig. 4E, F). On the other hand, R447G exhibited no discernible gate opening, not even the partial gate opening observed for WT in the absence of Mg^2+^ (Fig. 4B, C). Furthermore, R447G not only resisted gate opening, consistent with the MD simulation results (Supplementary Fig. 9), but also prevented the DNA hairpin from fully opening, indicated by the negative average extension change (dEx), likely by binding to both strands and stabilizing the DNA hairpin (Fig. 4E, F). These results are also in line with ensemble DNA cleavage and decatenation measurements in the absence of Mg^2+^. 2E2Q exhibited at least two-fold higher single-stranded DNA cleavage than WT *ec*topo3, consistent with an increased propensity of gate opening and therefore reduced religation (Fig. 4D). Conversely, R447G exhibited significantly less single-stranded DNA cleavage than WT, consistent with an increased propensity of gate closure and lack of gate motion, both favoring religation. These differences were not due to altered ssDNA binding affinities as the apparent *K*_D_ of the 2E2Q and R447G mutants were comparable to that of the WT enzyme (Supplementary Fig. 10). The decatenation activity of 2E2Q was also two-fold higher than that of WT in the absence of Mg^2+^, whereas decatenation activity of R447G was two-fold less than WT, further confirming the correlation between gate dynamics and catalytic activity (Fig. 4D).

At optimal Mg^2+^ (3 mM), 2E2Q displayed a broader distribution of open/closed lifetimes and slower overall gate dynamics than WT (Supplementary Fig. 11A, B) resulting in reduced religation and decatenation activities (Supplementary Fig. 11C, D) despite increased DNA cleavage (Supplementary Fig. 12). Over the range of Mg^2+^ concentration, 0.3–10 mM, 2E2Q exhibited slower, Mg^2+^-insensitive gate dynamics, supporting the hypothesis that the two acidic residues are essential for Mg^2+^-dependent gate conformational dynamics. R447G had longer closed and shorter open state lifetimes than the WT enzyme, consistent with predictions of the salt-bridge disruption model (Supplementary Fig. 11A, B). The longer-lived closed state, and the associated increase in religation rate of the R447G mutant, are consistent with the ~ two-fold reduction in decatenation activity as compared to the WT enzyme (Supplementary Fig. 11C, D). Additionally, both salt-bridge mutants exhibited largely Mg^2+^ independent decatenation above 0.3 mM Mg^2+^, the threshold at which DNA transphosphorylation is no longer limiting. In summary, these mutagenesis experiments confirm that optimal gate dynamics and enzymatic activity depend on Mg^2+^- mediated salt-bridge reconfiguration, highlighting a critical mechanism by which protein conformational dynamics are regulated by environmental Mg^2+^.

## Discussion

In this study, we uncover a previously unappreciated mechanism by which magnesium ions regulate protein conformation and function, potentially through dynamic modulation of a conserved salt-bridge network in *E. coli* topoisomerase IA. Our findings demonstrate that transient Mg^2+^ binding may disrupt specific inter-domain salt-bridges, facilitating conformational changes critical for protein-gate dynamics, thereby controlling enzymatic activity (Fig. 3, Supplementary Fig. 7). Neutralization of two acidic residues (E454Q and E457Q) within the divalent metal-binding triad disrupted the salt-bridge interaction between domains II and III and eliminated the Mg^2+^ requirement for gate opening and dynamics, increasing DNA decatenation in the absence of Mg^2+^ (Fig. 4, Supplementary Fig. 9). The slower, Mg^2+^-insensitive gate dynamics seen in 2E2Q in the presence of Mg^2+^ (Supplementary Figs. 11, 12) indicate that E454 and E457 contribute to Mg^2+^-dependent gate regulation. On the other hand, mutation of R447 to G447 hinders protein-gate opening, verifying the role of R447 in competing with the inter-domain salt-bridge configuration and facilitating gate opening (Fig. 4, Supplementary Fig. 9).

**Fig. 5:**
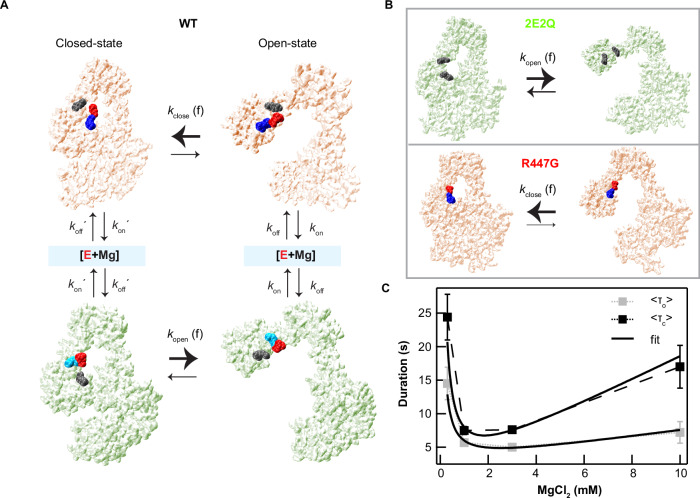
Mg-dependent salt-bridge switch controls gate dynamics. **A** Mg-dependent salt-bridge switch model: Mg binding alters the salt-bridge interactions of the acidic residues in domain II with inter- and intra-domain basic residues and catalyzes switching between two different salt-bridge configurations (open competent and closed competent). The residues that are involved in the salt-bridges for each configuration are colored in either red, blue, or cyan, whereas the uninvolved residues are indicated in gray. In the closed-state, the interdomain salt-bridge between domains II and III (red-blue spheres; closed competent) works as a latch that hinders the opening of the protein-gate. Mg transiently interacts with the acidic residues (indicated as [E+Mg]), disrupting the salt bridge and facilitating the salt-bridge to switch between the two configurations (closed vs open competent). Salt-bridge formation with R447 (red-cyan spheres; open competent) promotes transitioning of the gate to the open state. Similarly, in the open-state, Mg binding facilitates transitioning to the “closed competent” salt-bridge configuration, promoting the closed-state transition. At low Mg concentrations, the reduced rate of Mg binding results in longer open and closed state durations. At high Mg concentrations, Mg-dependent transitions to either the open or closed state would slow down as the higher occupancy of the [E+Mg] state would decrease the effective switching frequency, increasing both the closed and open state durations. **B** The acidic residue mutations (2E2Q) disable the “closed-competent” configuration, which inactivates the Mg-dependent gate latch function and allows unregulated gate opening (top). The R447 mutation disables the “open competent” configuration, which promotes the closed state (bottom). **C** Salt-bridge switch kinetic model (black lines) (supplementary info) recapitulates the Mg-dependent characteristics of the experimentally determined open and closed gate lifetimes (gray and black squares, respectively). Number of biological replicates: 3, 5, 5, and 4 for 0.3, 1, 3, and 10 mM Mg, respectively. Error bars correspond to the standard deviation.

Based on these results, we developed a mechanistic model and related kinetic scheme to explain the observed magnesium-dependent gate dynamics and enzymatic activity of *ec*topo3 (Fig. 5, Supplementary Fig. 13 and supplementary information). The important aspects of the model posit that transient Mg^2+^ binding to the acidic triad disrupts the existing salt-bridge configuration and that gate transitions are coupled to switching between salt-bridge configurations. This model captures the unusual parabolic Mg-dependence of gate dynamics and activity: switching between states requires the breaking of one salt-bridge and the formation of another. At low Mg^2+^ concentration, the rate of salt-bridge breaking is limiting, whereas at high Mg^2+^ concentration, the rate of salt-bridge formation is limiting. This model naturally results in an optimum Mg concentration at which the rates of salt-bridge disruption and formation are balanced. Fitting of the associated kinetic scheme quantitatively captures the Mg^2+^-dependence of the gate kinetics (Fig. 5C, Supplementary Information).

Our study reappraises Mg^2+^ biological roles beyond facilitating transphosphorylation of nucleolytic process, revealing an additional regulatory mechanism in *E. coli* TopIA through influencing protein conformational dynamics. As magnesium is vital to cellular function and survival, regulatory roles of Mg^2+^ are prevalent in enzymes that are involved in magnesium homeostasis and transport, in addition to cell cycle regulation and proliferation^17–19,44,45^. The detailed mechanisms of magnesium-dependent regulation are not fully understood, but prior studies suggest that magnesium may trigger protein conformational changes through interactions with salt-bridges in response to local Mg^2+^ concentration^44–47^. As salt-bridges are important factors stabilizing protein structure and regulating conformational dynamics^41,48^, magnesium-dependent disruption of salt-bridges represents a general and adaptable mechanism to regulate protein stability and conformational dynamics.

Whereas the current study identified magnesium -dependent protein conformational dynamics in both *E. coli* TopIAs, the magnesium -dependent regulation scheme could be mechanistically conserved among bacterial TopIA enzymes. Previous analysis of the TopIA family revealed semi-conserved acidic regions in multiple bacterial TopIA enzymes^49^ that contribute to the predicted divalent metal binding sites we identified in *ec*topo3 and *ec*topo1. Extending this analysis, we find that among bacterial topo3 enzymes, which share as low as 25% identity with *ec*topo3, there is a remarkable sequence and structural conservation of this potential divalent metal binding site overlapping with identified inter- and intra-domain salt-bridges^50^ (Supplementary Fig. 14). For *ec*topo1, sequence alignments of similar (>60%) bacterial topo1 reveal a high degree of sequence and structural conservation (Supplementary Fig. 15). Interestingly, bacterial topo1 with lower sequence similarity to *ec*topo1 possess a second acidic region that appears to be analogous to that in *ec*topo3, possibly participating in an inter-domain salt-bridge with basic residues in domain III (Supplementary Fig. 15).

Whereas the overlapping Mg-binding and dual salt-bridge architecture is not conserved at the sequence level in higher organisms, the lack of gate opening in the absence of Mg^2+^ observed for the human topo3α -RMI1-RMI2 (TRR) complex^12^ despite being able to cleave DNA in the absence of Mg^2+^^51^, suggests mechanistic conservation of Mg^2+^-regulated gate dynamics. In line with this, we identified a salt-bridge between K334 (domain III) and E528 (domain IV) that likely hinders opening of the protein-gate in TRR and an overlapping metal-binding acidic triad (E331, E333, and E528) (Supplementary Fig. 16) in human topo3α. This suggests that magnesium -dependent gate regulation is potentially functionally conserved in higher organisms, but this remains to be confirmed.

The model we propose describes a mechanism through which protein-gate dynamics are coupled to Mg^2+^ concentration with an optimum near physiological concentration estimates^19,52^. More importantly, considering the extensive involvement of TopIA in genomic DNA processing, Mg-dependent protein-gate control may represent a “fail-safe” mechanism to prevent DNA damage under magnesium starvation conditions. As TopIA, including human topo3α, can cleave DNA without Mg^2+^, high levels of DNA cleavage could accumulate if the protein-DNA gate is allowed to open unhindered, as observed with the 2E2Q mutant, which represents a poorly regulated protein gate.

In summary, we identified a layer of enzyme regulation mediated by networked salt-bridges and magnesium ions, underscoring the intricate interplay between protein structure, dynamics, and the cellular environment. This knowledge advances our understanding of enzyme function and opens avenues for rational drug and protein design. Although our data provide compelling evidence for Mg^2+^-mediated regulation in TopIA, further structural and biophysical studies are needed to resolve the dynamic interplay between divalent metal binding and salt-bridge switching at an atomic detail, particularly in eukaryotic homologs.

## Methods

### DNA substrates

#### Coilable “bubble” DNA for DNA supercoil relaxation assay

DNA substrates containing a bubble in the middle were generated by PCR of two 3 kb fragments using pET28b as template and digested with BsaI to create 5ʹ-end 4 nt overhangs (supplementary table 1). The two 3 kb DNA fragments were ligated to a preassembled DNA bubble to form 6 kb DNA. Biotinylated and digoxigenylated 500 bp DNA handles, generated as previously described, were ligated to each end of the 6 kb DNA. The DNA bubble was formed by annealing two DNA oligos (5ʹGCTTAGCTTAGAATCA TTTTTTTTTTTTTTTTTTTT TTTTT GCATCTAGACAGTGAC and 5ʹGTCACTGTCTAGATGC - GAT TTG GGA TGT – TGATTCTAAG CTAAGC)^53,54^.

#### Gapped DNA for protein-DNA gate activity measurements

DNA substrates containing a 37 nt single-stranded gap were generated similarly as previously described^33,55^. Briefly, 430 bp DNA segments were produced by PCR amplification of pKZ1, which contains two nt.BbvcI sites 37 nt apart on the same strand, with two primers that encode the BsaI recognition sequence (supplementary table 1). Restriction digestion of the DNA segments yields two different 5ʹ overhangs, each of which is complementary to the 5ʹ overhang of either a biotinylated or digoxigenated handle. After nicking with nt.BbvcI to produce a single-stranded DNA gap, the DNA segment was ligated to the 500 bp handles. DNA segments were digested with PstI to remove un-gapped DNA, as its recognition sequence is located between the two nicking sites, and the restriction endonuclease can cleave duplex but not single-stranded DNA. Intact DNA with both handles was purified by gel extraction.

#### 579 bp DNA hairpin substrate for complete gate opening measurement

A~ 597 bp DNA hairpin with biotin- and digoxigenin-labeled ends was constructed for single-molecule gate-opening experiments on an extended ssDNA segment similar to the previous study^33,56^. Plasmid pKZ1, which contains two nt.BbvcI sites 37 nt apart on the same strand was PCR amplified with two primers that encode BsaI recognition sequences (supplementary table 1). After digestion of the PCR product with BsaI for generating the differential complementary ends and nt.BbvcI for making a 37 nt single-strand DNA gap, a T-loop and a multi-digoxigenin labeled 166 bp DNA were ligated to each complementary end of the DNA segment to complete the hairpin (T-loop) and add a dig-labeled DNA handle for surface attachment. A 3´ biotin-labeled segment consisting of dT_40_ adjacent to 34 nucleotides complementary to the single-strand DNA gap was ligated to the 5´ end of the DNA gap to provide a biotin-labeled DNA handle.

### Supercoiled DNA relaxation assay

The 20 µl reaction mixtures for the relaxation assay that contained 4 nM of pBR322 and 80 nM enzymes with varying MgCl_2_ in the reaction buffer (20 mM Tris pH 8.0, 100 mM potassium glutamate, 1 mM Dithiothreitol) were incubated at 37 °C for 1 h. The reaction was stopped by adding 1% SDS and 4 units of proteinase K (New England Biolabs) and incubating at 42 °C for 3 h. 5 µl of individual reaction mixtures with 1 µl loading dye were loaded on a 1% agarose gel. After 13 h at 35 V, the agarose gel was stained with Sybr gold (Invitrogen) and its image was captured using a UVP imager. The fraction of the remaining intact supercoiled DNA per reaction conditions were quantified using Image J and Igor (Wave Metrics).

### Kinetoplast DNA (kDNA) unlinking assay

The 20 µl reaction mixtures for the decatenation assay that contained 2 µl of kDNA (K2003, Inspiralis) and 80 nM enzymes with varying MgCl_2_ in the reaction buffer (20 mM Tris pH 8.0, 100 mM potassium glutamate, 1 mM Dithiothreitol) were incubated at 37 °C for 1 h. The reaction was stopped by adding 1% SDS and 4 units of proteinase K (New England Biolab) and incubating at 42 °C for 3 h. 5 µl of individual reaction mixtures with 1 µl loading dye were loaded on a 1% agarose gel. After 13 h at 35 V, the agarose gel was stained with Sybr gold (Invitrogen) and its image was captured using a UVP imager. The fraction of the resolved kDNA per reaction conditions were quantified using Image J and Igor (Wave Metrics).

### Single-molecule supercoiled DNA relaxation assay

Detailed experimental procedures were previously described^53,54,57^. In brief, to prepare torsionally constrained (“coilable”) DNA tethers, 2.5 pM of multiple digoxigenin and biotin end-labeled DNA prepared as described above was mixed with 32 ng of anti-digoxigenin (Roche, 11333089001) in 40 µl 1 x PBS buffer for 30 min at room temperature. This mixture was introduced into a sample cell (~30 µl total volume) coated with a low concentration of stuck beads and incubated overnight at 4 °C. Unbound DNA was washed out with 400 µl of wash buffer (WB; 1 x PBS, 0.02% w/v BSA). 10 µl of 0.1% w/v streptavidin-coated magnetic beads (My One, Invitrogen) in WB was then introduced into the chamber and incubated for 1 hour to attach to the free biotinylated end of DNA. Unbound magnetic beads were washed out with 1 ml of WB and then 400 µl of topoisomerase buffer (TBB; 20 mM Tris, pH adjusted to 8.0, 100 mM potassium glutamate, 1 mM Dithiothreitol, 0.002% v/v Tween-20, and 0.02% w/v BSA) supplemented with 5 mM EDTA and 5 mM EGTA to remove divalent ions bound to DNA. After 10 min incubation to chelate divalent ions, the sample chamber was washed with 400 µl of TBB. Once coilable DNAs were identified based on the DNA extension reduction due to plectoneme formation upon twisting DNA by turning the magnets (Fig. 1C), 200 µl of 75 pM enzyme in TBB was introduced and after 5 min incubation, the chamber was washed with 400 µl TBB supplemented with the desired concentration of MgCl_2_ (0.3, 3, and 10 mM). All measurements were performed at a 0.2 pN force applied to the magnetic beads at room temperature. 15–20 right-handed turns were automatically introduced when all DNA tethers were fully relaxed, and the measurements continued for hours.

Relaxation rates were estimated by first obtaining the durations between the introduction of the 20 turns and the full DNA relaxation using custom-written scripts in Igor Pro 9. The statistical mean and standard deviation of these durations were calculated using built-in analysis in Igor^54^.

### Gate opening measurements of TopIA using 579 bp DNA hairpin

Similar to the previous study^15^, gate opening of multiple TopIA enzymes was measured using a 579 bp DNA hairpin under high force. The fully opened DNA hairpin provides a long stretch of single-stranded DNA region above a critical opening force (14–15 pN) under which the protein-gate of TopIA enzymes remains open in the presence of magnesium. 579 bp DNA hairpin tethers were prepared as described above. 1 µM of *ec*TopIA enzymes were preincubated in 1 mM EDTA first and then diluted to 1 nM in the TBB buffer containing either 1 mM EDTA or 3 mM Mg^2+^. Before the addition of enzymes, the average opening extension of the DNA hairpin was measured at 16 pN and 26 pN as a base-line without enzyme (Ex0). After the introduction of 1 nM enzyme, the extension changes (Ex) were continuously monitored for 10–30 min under periodic changes of forces from high (either 16 or 26 pN) to low (0.4 pN). 16 pN was applied as the high force, whereas 26 pN was applied when the extension was either equal to or less than Ex0 at 16 pN. The relative maximum extension changes (dEx) were obtained by subtracting Ex0 from the maximum extension change at high force to estimate protein-mediated DNA gate opening. In the presence of 1 mM EDTA, the high force duration was set at 5.1 min and the total measurement duration was 30 min to account for the slower DNA cleavage rate by TopIA. In the presence of 3 mM Mg^2+^ the high-force and total durations were 90 s and 10 min, respectively. After measurements in the presence of 1 nM enzyme (with either 1 mM EDTA or 3 mM Mg^2+^), the sample chamber was washed with 800 µl ( ~ 20 sample chamber volumes) of 1 mM EDTA followed by 200 µl of either 3 mM Mg^2+^ (for 1 nM enzyme with 1 mM EDTA condition) or 1 mM EDTA (for 1 nM enzyme with 3 mM Mg^2+^ condition).

### Single-molecule TopIA gate dynamics measurements

The procedure for preparing samples containing gapped DNA tethers was the same as described in the single-molecule supercoil relaxation assay section above. To measure force-dependent gate-opening kinetics of topIA, the positions of DNA tethered magnetic beads were tracked continuously in real time from CCD images captured at 200 Hz while force was increased in 1 pN increments from 3 pN to 10 pN over 15 min. To study the effect of magnesium, measurements were repeated after washing the chamber with 200 µl of topoisomerase buffer (20 mM Tris, pH adjusted to 8.0, 100 mM potassium glutamate, 0.3 –10 mM MgCl_2_, 1 mM Dithiothreitol, 0.002% v/v Tween-20, and 0.02% w/v BSA) without additional topIA. TopIA remained bound and active during repeated buffer exchanges over hours of measurements. The position of the sample cell with respect to the objective was actively stabilized by tracking the position of a fiducial bead affixed to the surface of the flow cell and moving the three-dimensional piezoelectric driven sample stage to compensate for drift^53^.

Open and closed states from the single molecule traces were detected using ICON^58^ and the individual state durations were obtained with a custom written analysis script in Igor Pro 9.

### Expression and purification of *E. coli* topoisomerase 3 and variants

The pET-15b^*topB*^ plasmid encoding a his-tagged K12 *E. coli* topoisomerase 3 was provided by J. Keck (University of Wisconsin Madison). A TEV protease cleavage site with an additional two glycine residue spacer was inserted upstream of the *topB* gene and downstream of the His-tag using the QuikChange II XL mutagenesis kit (Stratagene) and the sequence of the resulting plasmid (pET-15b^*topB+TEV*^) was verified.

#### Expression

*Expression*: Rosetta (DE3) pLysS Competent Cells (EMD Millipore) transformed with pET-15b^*topB+TEV*^ were grown at 37 °C in Luria-Bertani broth containing 50 μg/ml carbenicillin and 34 μg/ml chloramphenicol. Protein expression was induced with 1 mM isopropyl β-D-thiogalactopyranoside. After induction at 37 °C for 20 h, cells were harvested by centrifugation. The collected cells were suspended in buffer A (50 mM Tris HCl, pH 7.5, 300 mM NaCl, and 10% v/v glycerol) and emulsified using a high-pressure cell homogenizer (Avestin). The cell lysate was then centrifuged at 235,000 x *g* at 4 °C for an hour in a Ti45 rotor (Beckman). The supernatant was loaded onto a HisTrap-FF 5 ml Ni-NTA column (G.E.). His-tagged protein was eluted with buffer B (50 mM Tris HCl, pH 7.5, 0.5 M imidazole, 0.3 M NaCl, 10 mM β-mercaptoethanol, 10% v/v glycerol). The eluted proteins were concentrated and buffer-exchanged with buffer A using an Amicon Ultra-15 centrifugal filter unit with Ultracel-50 membrane (Millipore). The His tag was removed from the protein by digestion with 0.5 unit/μg Pro-TEV (Promega) for 6 h. The protein digestion mixture was run twice through a Ni-NTA column (Qiagen) to remove Pro-TEV and uncleaved protein. The size and purity of cleaved topo3 enzymes were evaluated by SDS PAGE (Invitrogen).

Two topo3 variants (2E2Q: E454 and E457 mutated to Q454 and Q457, R447G: R447 to G447) were generated using a Q5 site-directed mutagenesis kit (NEB) with primers encoding two point mutations for 2E2Q (primer1: ggcagcaaacagcgcgatcaagaaaacgacgg; primer 2: taacagcgtgcgccagcctg) and one point mutation for R447G (primer 1: ctgaagcaggctggggcacgctgttagg; primer 2: caagaaaacgggctttagcgacaaatttgcct). The mutations were verified by sequencing. 2E2Q and R447G expression and purification were performed identically as the WT topo 3 protein.

### Steered molecular dynamics (SMD)

To access gate opening through molecular dynamics simulations, we employed all-atom SMD by applying a constant force on the catalytic tyrosine of *ec*topo3 and a harmonic constraint on the bound ssDNA and three amino acid residues that interact with the ssDNA on the other side of the gate. Initial structures were prepared by homology modeling with SWISS-MODEL^59^ of the crystal structures PDB 2O19 (*ec*topo3)^34,60^ as a template. The prepared structure was rotated so that the bound DNA backbone was aligned along the direction of applied force (*x-*axis*)* using VMD 1.9.3^61^. The Cα of the catalytic tyrosine, Y328 for *ec*topo3, was chosen as the SMD atom (the atom on which force was applied). Harmonic constraints (50 kcal/mol), to counteract the applied force, were added to CA atoms of protein residues proposed to stabilize bound DNA (103, 105 and 165 for t *ec*topo3)^60,62^ and C1 atoms of the 6^th^ and 7^th^ positions of the bound ssDNA for both enzymes. Before performing SMD, the system was first solvated in an explicit water box large enough to accommodate the open state of the protein gate using the VMD Solvate plugin and then neutralized by adding 100 mM Na^+^ ions with the VMD Autoionize plugin^61^ followed by the addition of 3 mM Magnesium ions. Next, the systems were minimized and then equilibrated in the NPT ensemble (*P* = 1 atm, *T* = 310 K) for 9.5 ns, followed by additional equilibration in the NVT ensemble for 19.2 ns (*T* = 310 K, *V* = 148 x 109 x 109 Å^3^ for *ec*topo3). SMD was performed using NAMD (v2.14) for 300 ns during which the SMD atom was under a constant force of 50 pN^42,63,64^, and the trajectories of all atoms were recorded in DCD format every 10 ps. In order to satisfy the convergence criteria (reproducibility, structural convergence, and experimental relevance), 8 independent replicate simulations per condition were performed, and the experimentally comparable metric (gate conformational change) was determined from the simulation results. Salt-bridge detection in the simulation trajectories was performed using the VMD Salt Bridges plugin^61^, which identified all salt-bridge-forming sidechain pairs and provided the center-of-mass distances (COM) between these pairs at each time step of the trajectories. The Cα atom trajectories of the catalytic tyrosine for generating gate conformational changes in x and y were obtained with the Python script based on ProDy^65^, which calculated the COM of all atoms within 5 Å of the catalytic tyrosine Cα atom. The x and y position probability density histograms, the generation of plots and statistical analysis were performed in Igor Pro 9 (Wavemetrics).

### Reporting summary

Further information on research design is available in the Nature Portfolio Reporting Summary linked to this article.

## Supplementary information

Supplementary Information

Reporting Summary

Peer Review file

## Source data

Source Data 1

Source Data 2

Source Data 3

Source Data 4

Source Data 5

Source Data 6

Source Data 7

Source Data 8

## Data Availability

All data that support the findings of this study presented in the manuscript and supplementary information file are provided as source files. Source data are provided with this paper and in Figshare at 10.25444/nhlbi.31127188.
