# Supplementary material for: Networked salt-bridges mediate magnesium-dependent conformational dynamics and functional regulation in type IA topoisomerases
Source: Nat Commun. 2026 Apr 30;17:5907. doi: 10.1038/s41467-026-72556-9 (PMC13338365; doi:10.1038/s41467-026-72556-9)

Networked Salt-Bridges Mediate Magnesium-Dependent Conformational Dynamics and Functional Regulation in Type IA Topoisomerases

Yeonee Seol[1], Yuk-Ching Tse-Dinh[2], Keir C. Neuman[1]*

*Corresponding author: neumankc@nih.gov

The PDF file includes:

Supplementary Note
Supplementary Table 1
Supplementary Figs. 1 to 16
References 1-17

Supplementary Note

Magnesium dependent *ec*topo3 DNA cleavage and religation

To obtain the magnesium-dependent ssDNA cleavage and religation kinetics, we first generate a covalent enzyme-DNA cleavage complex by incubating negatively supercoiled plasmid with *ec*topo3 in the absence of magnesium, which supports cleavage but not religation. We then measure the reduction in cleaved DNA over time under different magnesium concentrations (Supplementary Fig. 2A), from which the cleavage and ligation kinetics, and equilibrium can be obtained. In detail, enzyme-DNA covalent cleavage complexes were prepared by incubating DNA with *ec*topo3 at 37°C for 30 min (900 nM negatively supercoiled pBR322 and 18 µM enzyme in 20 µl of topoisomerase buffer supplemented with 1 mM EDTA to prevent religation after cleavage). 10 µl of the enzyme-DNA cleavage complex was pipetted into each tube containing 90 µl of topoisomerase buffer with different $MgCl_2$ concentrations (0, 0.3, 3, and 10 mM) preequilibrated at 37°C. For each time point (0, 15, 30, 60, 150 and 300 s) 10 µl from each of the 4 different $MgCl_2$ concentrations was transferred to a new tube containing 1 µl of 10% SDS and 4 units of Proteinase K for inactivation. Inactivation was performed at 42°C for 2 hours. The religation samples were run on a 1% agarose gel for 2 hours at 80V. The gel was stained with Sybrgold and the image was analyzed with Fiji to quantify the cleaved DNA bands on the gel for each condition [1].

The change in the cleaved DNA over time was fit with Eq. s1 (Supplementary Fig. 2A top).

$$[C] \underset{k_C}{\overset{k_L}{\rightleftarrows}} [L];$$

$$\frac{d[C]}{dt} = k_C[L] - k_L[C]; [L] = [total\ DNA] - [C]\ ;$$

$$\frac{[C]}{[total\ DNA]} = \frac{k_L}{k_C+k_L}\exp(-(k_C+k_L)t) + \frac{k_C}{k_C+k_L} \qquad \text{Eq. s1}$$

$$K_{L/C} = \frac{k_L}{k_C}$$

$[C]$ indicates the cleaved DNA concentration and $[L]$ is the ligated DNA concentration. k_C and k_L are the cleavage and religation rates respectively. k_C, k_L, and the religation and cleavage equilibrium, $K_{L/C}$ for different Mg^{2+} were obtained from the fits (Supplementary Fig. 2B).

Single-stranded DNA binding affinity measurements using fluorescent polarization anisotropy (FPA)

FPA measurements were performed with 5′ fluorescein labelled dT55 single-stranded DNA. Using a 96 well plate, varying enzyme concentrations (0 – 8 nM) and Mg^{2+} concentrations (0 – 10 mM) in topoisomerase buffer (20 mM Tris, pH adjusted to 8.0, 100 mM potassium glutamate, and 1mM Dithiothreitol) were added to individual 96 wells containing 2 nM ssDNA and the reactions were incubated at room temperature for 30 min prior to the measurement. Measurements were repeated three times.

Statistical analysis of SMD simulations to estimate the gate opening transition probabilities and the average waiting times prior to gate opening

For simplicity, we assume that the protein gate opening transition is a single energy barrier crossing event, which results in an exponential waiting time distribution with an average rate, λ. The average rate can be significantly overestimated if the waiting times for the simulations with opening transitions are considered to obtain the opening rate estimate since many of the simulations end before the gate opens. If the simulation durations are all the same, Tm, then the average probability of opening in a simulation duration is $\overline{p}$:

$$\overline{p} = \int_0^{Tm} \lambda \exp(-\lambda t)\ dt = 1 - exp(-\lambda Tm)$$

Thus, we can estimate the rate of opening by determining the average probability of opening in the simulation time *Tm*. Given this average probability of opening for each simulation, individual simulations can be treated as a Bernoulli trial with opening probability *p* and the probability of observing *k* openings in *n* simulations can be described with the binomial probability mass function.

Based on Bayes's theorem, the posterior conjugate of the binomial likelihood is a beta distribution [2]. Setting the beta uniform distribution β (1, 1) as a conjugate prior, the gate opening probability density distribution is calculated as:

$$PDF\ (p;\ n, k) = \frac{p^k (1-p)^{n-k}}{\beta\,(1, 1)}$$

The expected probability, $\overline{p}$ and standard deviation, σ_{p_k} are

$$\overline{p} = \frac{k+1}{n+2}$$

$$\sigma_{p_k} = \frac{(k+1)(n-k+1)}{(n+2)^2(n+3)}$$

Thus, we can estimate the expected waiting time based on the expected probability and the standard deviation for the given simulation time derived from the number of opening events, *k*, observed in *n* simulations. The average waiting time, $1/\lambda$ and the standard deviation are calculated with the average probability, $\overline{p}$ and its standard deviation, σ_{p_k} for Supplementary Fig. 9B as below:

$$T_{wait} = \frac{1}{\lambda} = \frac{-Tm}{\ln(1-\overline{p})}$$

$$dT_{wait} = \frac{Tm}{(1-\overline{p})[\ln(1-\overline{p})\ ]^2}\,\sigma_{p_k}$$

Analysis of the Mg-dependent gate open and closed state durations arising from the salt-bridge switch model

Based on the Mg-dependent salt-bridge switch model (Fig. 5A and Supplementary Fig. 13), we calculate the open and closed time durations (τ_O and τ_C) from the transition rates between states in the kinetic scheme using a recursive relation [3,4]:

$$\tau_o = \frac{k_{on}\left(k_{off} + k_{on} + k_{close}\right) + k_{off}\left(k_{on} + k_{close}\right) + k_{off}k_{close}}{k_{on}k_{off}k_{close}}$$

$$\tau_c = \frac{k_c k'_{on}\left(k'_{off} + k'_{on} + k_{op}\right) + k_C k'_{off}\left(k'_{on} + k_{op}\right) + k_C k'_{off}k_{op} + k_L\left(k'_{off}k'_{on} + k'_{off}k_{op} + k'_{off}k_{op}\right) + k'_{on}k'_{off}k_{op}}{k_c k'_{on}k'_{off}k_{op}}$$

To test the proposed kinetic model, we fit the experimentally determined Mg- dependent gate dynamics (Fig. 2F) to the derived expressions for the open and closed state lifetimes (Fig. 5C). The aim is to qualitatively reproduce the parabolic Mg-dependence rather than accurately determine all fit parameters, which would be difficult considering the number of kinetic parameters, particularly for τ_C.

To simplify the fitting and reduce the number of fitting parameters, we set k_c and k_L to be proportional to the magnesium concentration, consistent with the ensemble measurement (Supplementary Fig. 2B). Additionally, we use the average $K_{L/C}$ from the ensemble measurements to relate k_c and k_L.

$$k_c = c([\mathrm{Mg}^{2+}])$$

$$k_L = < K_{L/C} > k_c$$

c was the only fit parameter related to the cleavage and religation rates. The other fit parameters are the Mg-binding (k_{on} and k'_{on}) and unbinding (k_{off} and k'_{off}) rates in the open and closed states, and the mechanical opening and closing rates, k_{open} and k_{close}, respectively.

Fitting parameters for the Mg-dependent τ_C and τ_O durations at a force of 5 pN:

τ_C		τ_O	
k'_{on}	310 $mM^{-1}s^{-1}$	k_{on}	0.7 mM^{-1} s^{-1}
k'_{off}	9.0 s^{-1}	k_{off}	0.8 s^{-1}
k_{open} (5 pN)	19.4 s^{-1}	k_{close} (5 pN)	1.6 s^{-1}
c	0.17 $mM^{-1}s^{-1}$		
$K_{d, Mg}$ (closed state)	29 µM	$K_{d, Mg}$ (open state)	1.1 mM

The high magnesium K_d values are consistent with weak Mg binding. The higher $K_{d, Mg}$ for the open state, $K_{d, Mg}$ (open state), suggests that the conformation of the open gate state renders Mg binding less favorable. Given the large uncertainties, simplifying assumptions, and simplified kinetic model, the fit displayed in Fig. 5C in the main text is primarily a demonstration that the kinetic scheme derived from the Mg-dependent salt-bridge model can quantitively describe the parabolic magnesium dependence of both the gate opening and closing rates with a single magnesium binding site. The actual fit parameters should be considered as estimates.

Docking of hexahydrated magnesium ion as a ligand to *ec*TopIA structures using AutoDock

The hexahydrated magnesium ion structure was extracted from PDB entry 1DNZ, which contains hexahydrated Mg^{2+} ions bound to DNA. The mean Mg^{2+}–O bond distance in the $[Mg(H_2O)_6]^{2+}$ (2.063Å) closely matches the experimentally determined value (2.069Å) reported in the studies of hydrated metal ion structures[5]. Prior to docking, the ligand's PDB was converted to PDBQT format suitable for docking using AutoDockTool. The hydrated version of the ligand was generated by replacing HOH atoms in the PDB file with "W" atoms (representing water in the modified AutoDock 4.2 force field) in the PDBQT file.[6]

Target protein structures that were used for BioMetall (Supplementary Fig. 7 and 8) were prepared following the standard AutoDock protocol.[6,7] A grid box (40Åx40Åx40Å) was placed around the ligand binding site, but the initial ligand position was randomized. Grid maps were generated for docking

including W affinity map. Docking was done using the default Genetic Algorithm search parameter and the modified AutoDock4.2 force field to include magnesium ion and water.[7]

The docking results were analyzed and the docking images were generated using AutoDockTools4[7].

Sequence alignments of *ec*topo1 and *ec*topo3

A total of 471 bacterial topoisomerase 3 sequences were obtained from UniProtKB[8] by searching under the taxonomy "bacteria," the gene name "topB," and the enzyme function "DNA topoisomerase". Redundant or closely related subspecies were excluded to ensure an unbiased selection of distinct identity groups for sequence comparison with *ec*topo3. These 471 sequences were aligned using the Muscle5 algorithm[9] in Jalview[10] and the resulting conservation and consensus sequences are presented in Supplementary Fig. 14.

A total of 962 bacterial topoisomerase 1 sequences with greater than 50% identity were obtained from UniRef clusters[8]. Redundant or closely related sequences from the initial 1000 sequences were removed to ensure an unbiased selection of distinct identity groups for sequence comparison with ectopo1. These 962 sequences were aligned using the Muscle5 algorithm[9] in Jalview[10], and the resulting conservation and consensus sequences are shown in Supplementary Fig. 15.

A total of 816 low identity (<50%) bacterial topoisomerase 1 sequences of were compiled from BLAST[8] searches using several representative entries from different identity groups (50%, 45%, 40%, 35%, 30%, 20%, and 15%). Redundant or closely related sequences were removed to ensure an unbiased selection of distinct identity groups. These 816 sequences were aligned using Muscle5 algorithm[9] in Jalview[10], and the resulting conservation and consensus sequences are shown in Supplementary Fig. 15.

Table S1: list of DNA oligos and PCR templates that were used for generating substrates.

All oligonucleotides were custom ordered from Eurofin Genomics.

Coilable bubble DNA	
3kb biotin side left primer	GGGTCTCGCAACCTCAGTACAATCTGCTCTGATG
3kb biotin side right primer	GGGTCTCGCCCATCCGGATATAGTTCCTCCTTTC
3kb digoxigenin side left primer	GCTGGGTCTCGCCACTTAATCGCCGCGACAATTTGCGACG
3kb digoxigenin side right primer	GCTGGGTCTCGACCAACGCTCAAGTCAGAGGTGGCGAAAC
DNA bubble top	5´ phos-TGGGGCTTAGCTTAGAATCA TTTTTTTTTTTTTTTTTTTT TTTTT GCATCTAGACAGTGAC
DNA bubble bottom	5´ phos-GTGGGTCACTGTCTAGATGC GAT TTG GGA TGTTGATTCTAAG CTAAGC
PCR template	pET28b+ (Novagen, 69865-M**)**
Gapped DNA	
438 bp for primer	GCTGGGTCTCGACCACGGATATAGTTCCTCCTTTC
438 bp rev primer	GCTGGGTCTCGCAACTTGTGAGCGGATAACAATTC
Gapping oligo	GGCGCAGCTTCCGACTGCAGCCTGACGCCAGGGCTGA
PCR template	pKZ1
579 bp DNA hairpin	
DNA hairpin dig-handle side primer	GCTGGGTCTCGACCA CGGATATAGTTCCTCCTTTC
DNA hairpin T-loop side primer	GCTGGGTCTCGCAACGCGAAAGGTTTTGCGCCATTCG
T-loop	5´ phos-GTTGCATGGCAGTAGGTTTTCCTACTGCCATG
3´Bio polydT40	5´ phos-TCAGCCCTGGCGTCAGGCTGCAGTCGGAAGCTG-poly T (40)
PCR template	pKZ1
Multi-bio or dig labelled DNA handles	
Multi-biotinylated handle primer	GCTGGGTCTCGGTTGTTCCCTTTAGTGAGGGTTAATTG
Multi-digoxigenin handle primer	GCTGGGTCTCGTGGTTTCCCTTTAGTGAGGGTTAATTG
Reverse primer	TATAGTCCTGTCGGGTTTCG (500 bp) GAGTTAGCTCACTCATTAGGCACCC (166 bp)
PCR template	pKZ1

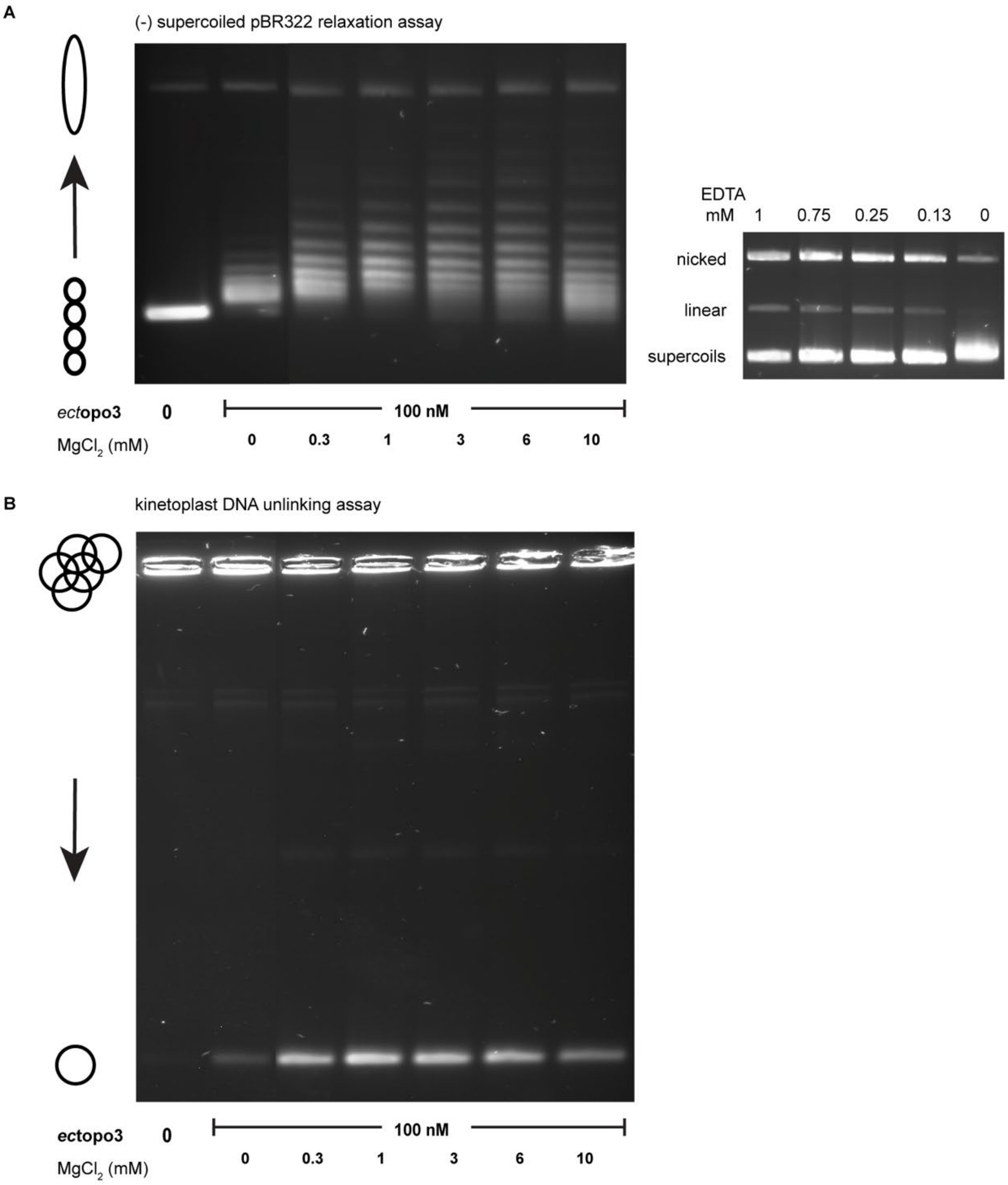

Supplementary Fig. 1. **A.** Example agarose gel (1%) displaying *ec*topo3 relaxation of negatively supercoiled plasmid DNA as a function of Mg concentration (methods). Inset: titrating EDTA to determine the concentration required to deplete divalent metal ions (1 mM) as evidenced by the lack of relaxation. **B.** Example agarose gel (1%) displaying *ec*topo3 decatenation of kDNA as a function of magnesium concentration. We note that supercoil relaxation and unlinking activity were detected without the addition of magnesium to the reaction ($MgCl_2$=0). This is likely due to trace magnesium or other divalent metal ions present in the buffer. Catalytic activity, but not DNA cleavage, was inhibited by 1 mM EDTA that chelates Mg^{2+}.

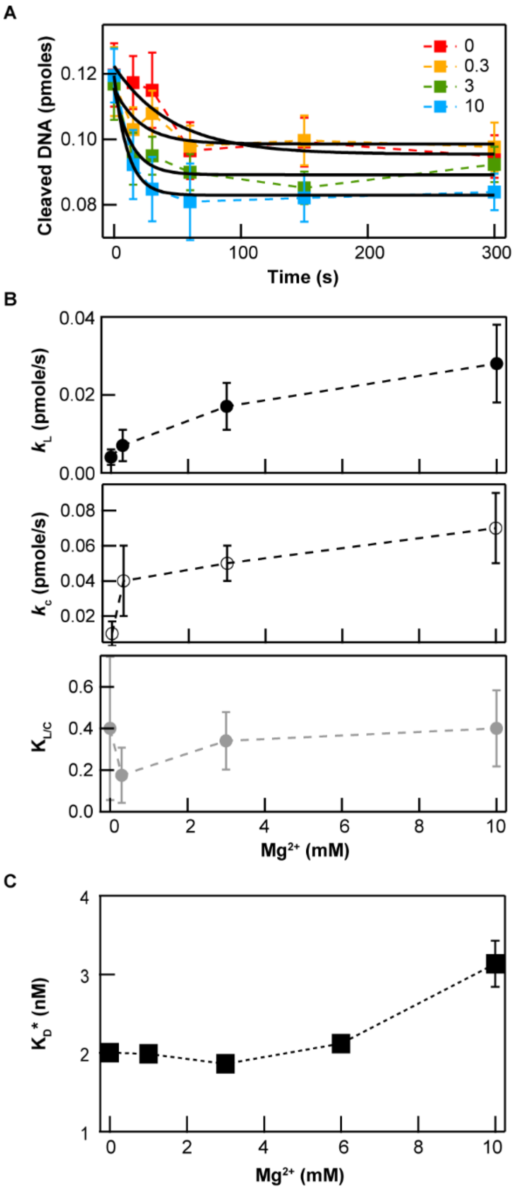

Supplementary Fig. 2. Ensemble measurement of *ec*topo3 religation-cleavage equilibrium. **A.** The amount of cleaved DNA substrate over time as a function of Mg^{2+} concentration (markers correspond to Mg^{2+} concentration from 0 to 10 mM) fit with eq. s1 (Supplementary Note). The error bars correspond to SEM (N=5 samples). **B.** The effective ligation and cleavage rates (k_L and k_c, respectively) in addition to the religation-cleavage equilibrium ($K_{L/c}$) as a function of Mg^{2+} for *ec*topo3 obtained from the fits to the data in panel A. Both k_L and k_C increased monotonically with Mg^{2+} concentration above 0 mM. On the other hand, $K_{L/c}$ remained largely independent of Mg^{2+}. The error bars correspond to fitting errors. **C.** Single-stranded DNA binding affinity of *ec*topo3 as a function of Mg^{2+} concentration (Supplementary Note). The error bars correspond to SEM (N=3 samples).

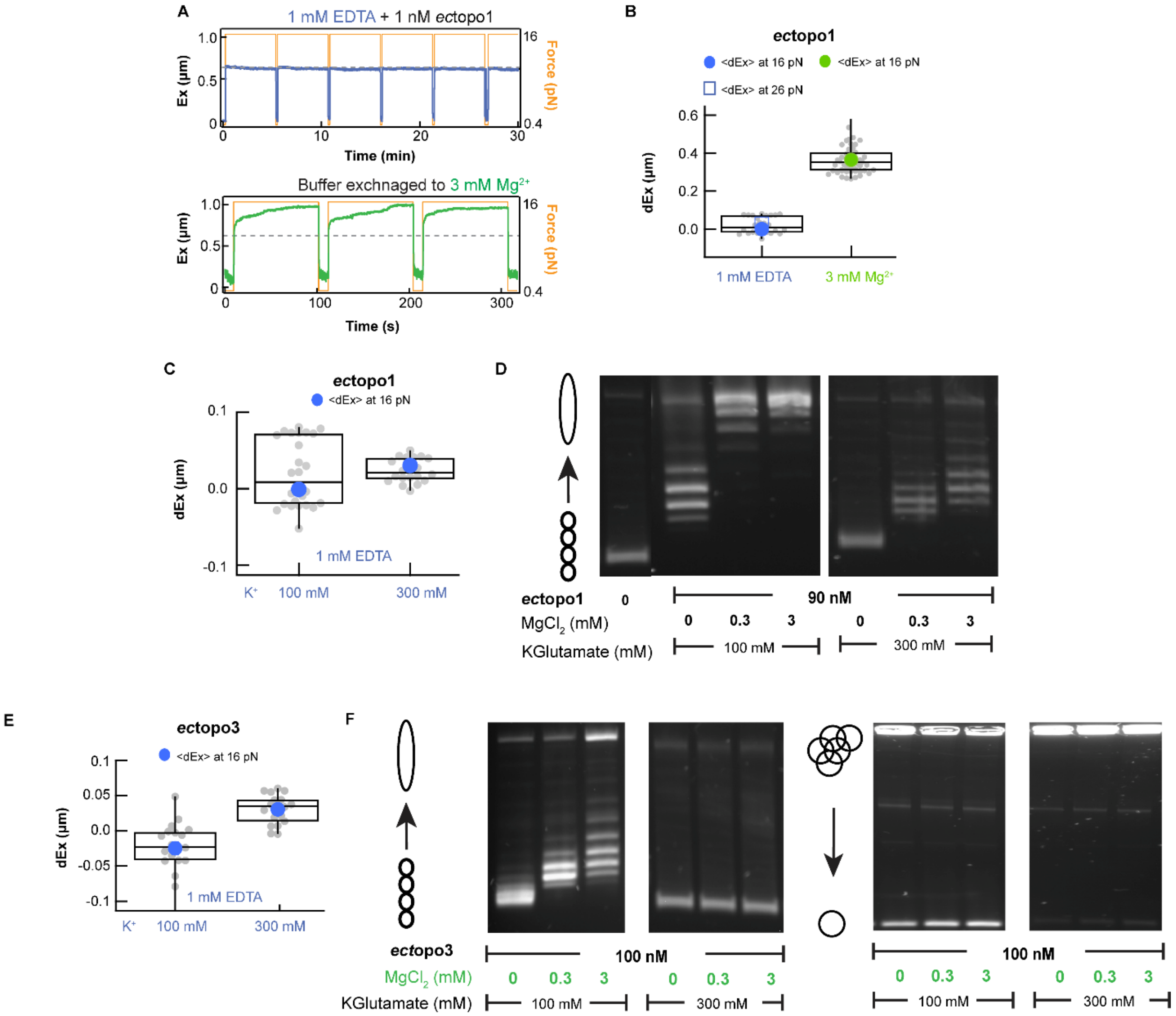

Supplementary Fig. 3: **Full gate opening of *ec*topo1 requires magnesium**. **A.** Example trace of *ec*topo1 gate opening measurement at 1 mM EDTA (blue line) showed no change of DNA extension, indicating no gate-opening (left panel). After removing 1 mM EDTA by introducing protein-free buffer with 3 mM Mg^{2+}, the DNA extension (green line) increased, indicating gate-opening. Force changes (orange line) are overlayed with the extension plots. **B.** Box plots of extension change (dEx) at 1 mM EDTA and 3 mM Mg^{2+} (gray points and black solid lines). The corresponding averages of dEx, <dEx> at 1 mM EDTA under 16 pN and 26 pN (blue filled circles and blue open squares, respectively) and at 3 mM Mg^{2+} under 16 pN (green filled circle) are overlayed on the box plots. Number of events (number of biological replicates) : 26 (3) for 1 mM EDTA; 51 (3) for 3 mM Mg^{2+}. Data are presented as box plots showing the median, the 25th–75th percentiles, and the minimum and maximum values. **C.** High monovalent salt did not support *ec*topo1 gate opening. Box plots of extension change (dEx) at 100 mM and 300 mM KGlutamate under magnesium depletion conditions (1 mM EDTA). Number of events (number of biological replicates) : 26 (3) and 20 (3) for 100 mM and 300 mM KGlutamate respectively. Data are presented as box plots showing the median, the 25th–75th percentiles, and the minimum and maximum values. <dEx> data (blue filled circles) for 100 mM and 300 mM KGlutamate were overlayed on the box plots. **D.** Increasing potassium monovalent salt from 100 to 300 mM became inhibitory for *ec*topo1 supercoil relaxation activity. N = 1 biological replicates. **E.** High monovalent salt did not support *ec*topo3 gate opening. Box plots of extension change (dEx) at 100 mM and 300 mM KGlutamate under magnesium depletion conditions (1 mM EDTA) (gray points and black solid lines). Number of events

(number of biological replicates) : 19 (3) and 18 (3) for 100 mM and 300 mM KGlutamate respectively. Data are presented as box plots showing the median, the 25th–75th percentiles, and the minimum and maximum values. <dEx> data (blue filled circles) for 100 mM and 300 mM KGlutamate were overlayed on the box plots. **F.** Increasing potassium monovalent salt from 100 to 300 mM became inhibitory for both supercoil relaxation (left) and decatenation (right) activities by *ec*topo3. N = 1 biological replicates.

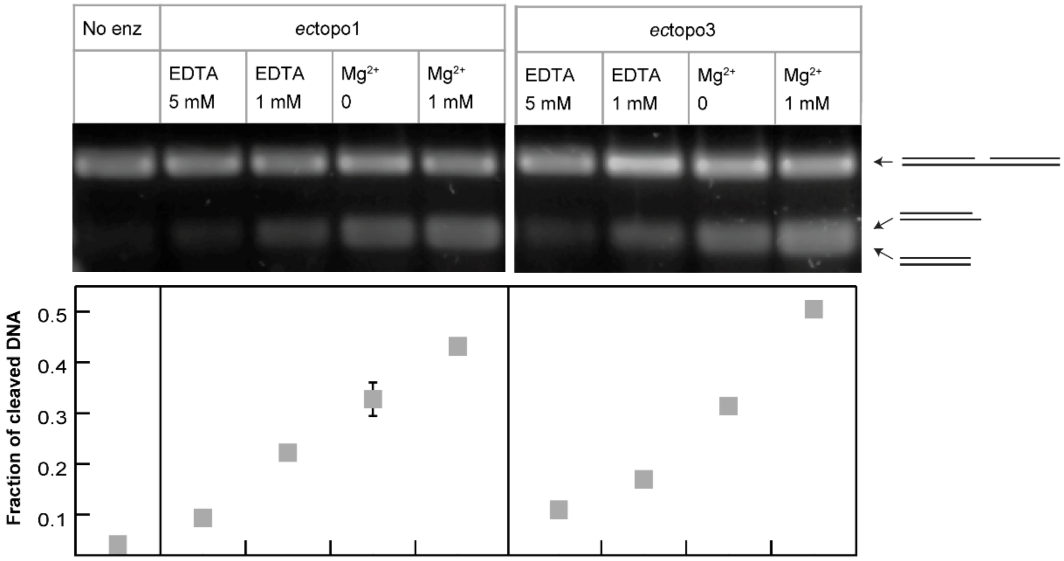

Supplementary Fig. 4: **Ensemble cleavage of the gapped DNA substrate used in the single-molecule TopIA gate dynamics measurements**. Both *ec*TopIA cleaved gapped DNA substrates under different conditions (30 minute incubation). The ensemble cleavage reactions were visualized on a 2% agarose gel (top). The quantitative fractions of cleaved DNA corresponding to each gel lane are shown below the gel. The error bars correspond to fitting errors. N = 1 biological replicate.

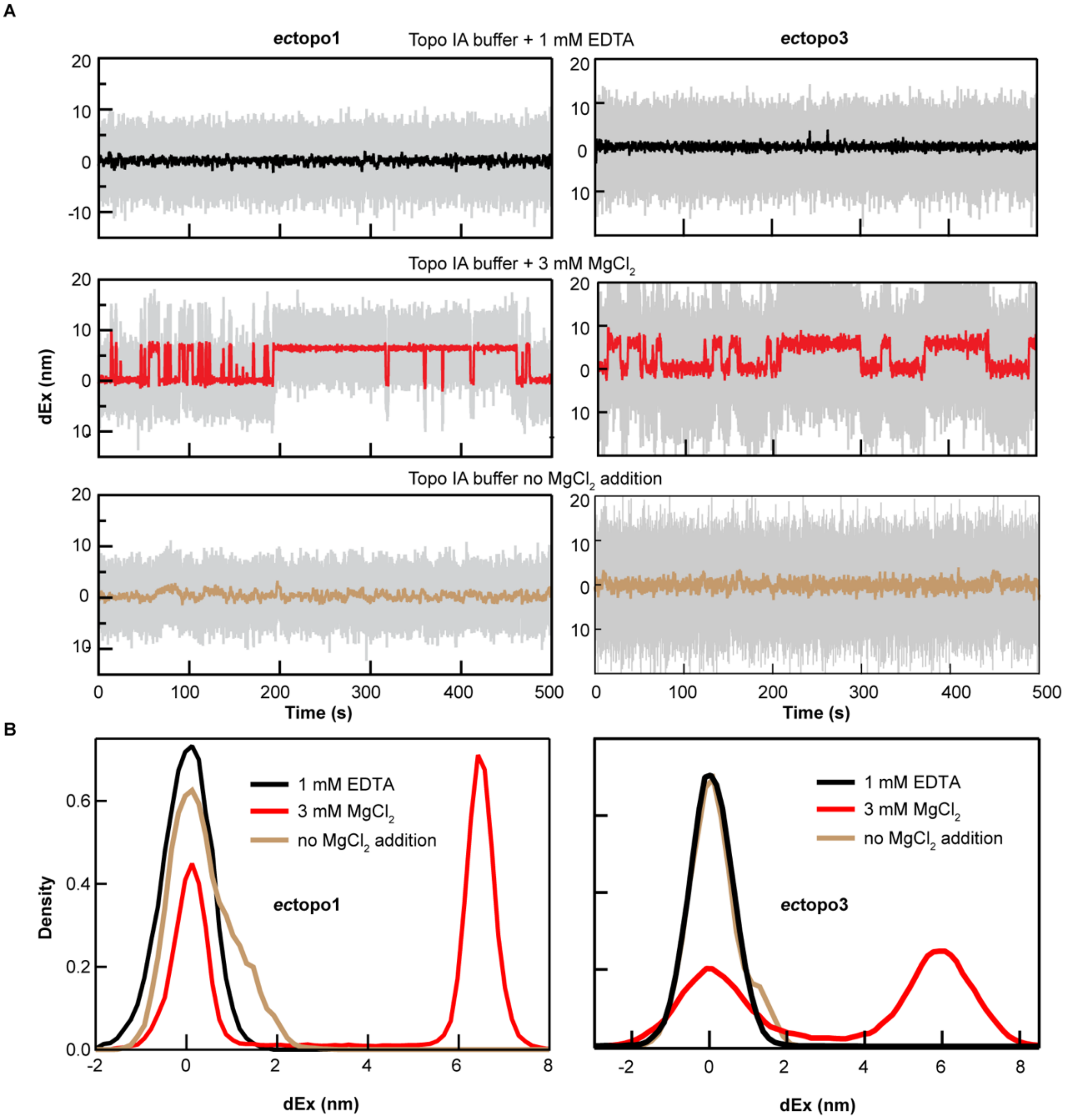

Supplementary Fig. 5. Both *ec*topo1 and *ec*topo3 require Mg^{2+} for complete gate opening and dynamics. A. Example traces with buffer supplemented with 1 mM EDTA, 3 mM Mg^{2+}, or in the absence of Mg^{2+}. **Top.** No visible extension changes were observed for either topIA enzyme under Mg depletion conditions (1 mM EDTA). **Middle.** ~6 nm extension fluctuations associated with gate dynamics were observed for both TopIA enzymes in the presence of Mg. **Bottom.** Occasional small fluctuations (1-2 nm) were observed without added Mg^{2+}. A force of 7 pN was applied during *ec*topo1 measurements, while 5 pN was used for *ec*topo3 measurements, as *ec*topo1 requires a higher force to achieve equilibrium gate dynamics compared to *ec*topo3. **B.** The probability density distributions of the example traces shown in **A**.

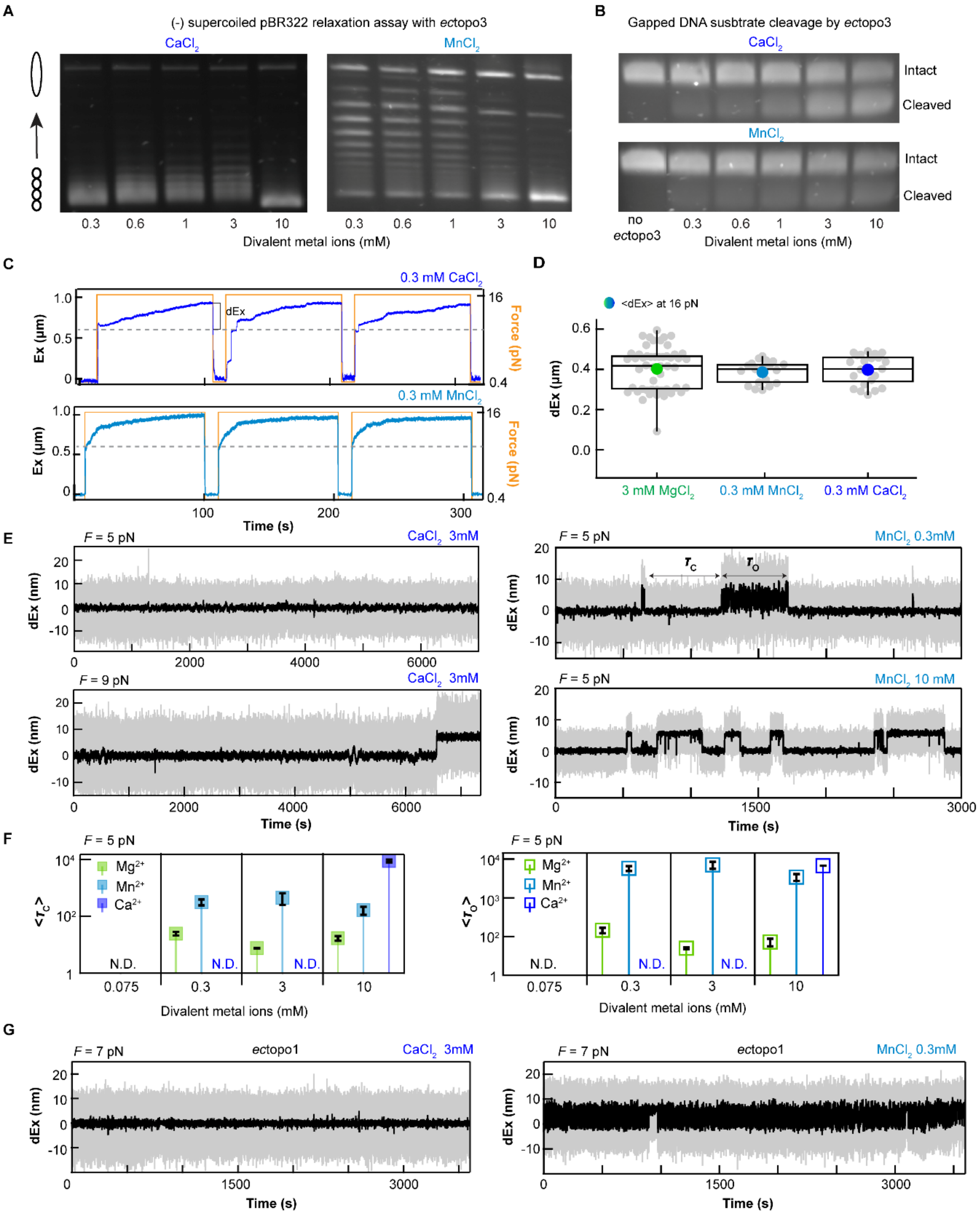

Supplementary Fig. 6: Mn^{2+}, but not Ca^{2+}, supports gate dynamics of both *ec*topo1 and *ec*topo3. **A.** Example agarose gel (1%) displaying *ec*topo3 relaxation of negatively supercoiled plasmid DNA as a function of $CaCl_2$ and $MnCl_2$. The relaxation activity of *ec*topo3 was generally less efficient with Mn^{2+} and Ca^{2+} than with Mg^{2+}, though the processivity was higher at low Mn^{2+} concentrations. N = 1 biological replicate. **B**. Ensemble cleavage of gapped DNA substrate as a function of different divalent metal ion concentrations (30 minute incubation). The ensemble cleavage reactions were visualized on a 2% agarose gel. Both Mn^{2+} and Ca^{2+} allow efficient single-stranded DNA cleavage by *ec*topo3. N = 1 biological replicate. **C.** Example traces of *ec*topo3 gate opening measurements in the

presence of 0.3 mM $CaCl_2$ (top; light blue solid line) and $MnCl_2$ (bottom; cobalt blue solid line). The measurements were taken after removing 1 mM EDTA by introducing protein-free buffer with 0.3 mM of either $CaCl_2$ or $MnCl_2$, respectively. The DNA extension increased above the dashed line (the extension of the fully opened DNA hairpin), indicating multiple gate-opening events. Force changes (orange line) are overlayed with the extension plots. **D.** Box plots of extension change (dEx) with $MgCl_2$, $MnCl_2$ and $CaCl_2$. (gray points and solid lines). The corresponding averages of dEx, <dEx> at 3 mM $MgCl_2$ (green filled circle), 0.3 mM $MnCl_2$ (light blue filled circle), and 0.3 mM $CaCl_2$ (cobalt blue filled circle) are overlayed on the box plots. Number of events (number of biological replicates) : 52 (3); 19 (3); 23 (3) for 3 mM $MgCl_2$, 0.3 mM $MnCl_2$ and 0.3 mM $CaCl_2$. Respectively. Data are presented as box plots showing the median, the 25th–75th percentiles, and the minimum and maximum values. **E.** Examples of gate dynamics measurements at 3 mM $CaCl_2$ under 5 pN tension (top left) and 9 pN tension (bottom left); 0.3 $MnCl_2$ mM (top right), and 10 mM $MnCl_2$ (bottom right) under 5 pN tension. No gate conformational changes were observed in the presence of $CaCl_2$ at 5 pN. Whereas the gate activity was infrequent under all $MnCl_2$ concentrations, rapid gate fluctuation states were observed at 0.3 mM $MnCl_2$, which disappear at higher $MnCl_2$ concentrations. **F.** The average durations of the closed (τ_c) and open (τ_o) states obtained from the single-molecule trajectories measured at an equilibrium force of ~5 pN for $MgCl_2$ (closed: green filled square; open: green open square), $MnCl_2$ (closed: light blue filled square; open: light blue open square), and $CaCl_2$ (closed: cobalt blue filled square; open: cobalt blue open square). Number of events (number of biological replicates): 61 (3); 313 (5); 58 (4) for 0.3, 3, and 10 mM $MgCl_2$ respectively. Error bars correspond to the standard deviation. Number of events (number of biological replicates): 0 (3), 0 (3), 0 (3), 1(4) for 0.075, 0.3, 3, 10 mM $CaCl_2$ respectively. 0 (3), 36 (3), 6 (3), 20 (4) for 0.075, 0.3, 3, 10 mM $MnCl_2$ respectively. Error bars correspond to the standard error of the mean. **G**. *ec*topo1 displays similar gate dynamics characteristics as *ec*topo3 with Ca^{2+} and Mn^{2+}. No gate dynamics were observed with Ca^{2+} (left) while the rapid gate domain fluctuations persisted with Mn^{2+} (right) for the entire measurement duration (1 hour) at 7 pN.

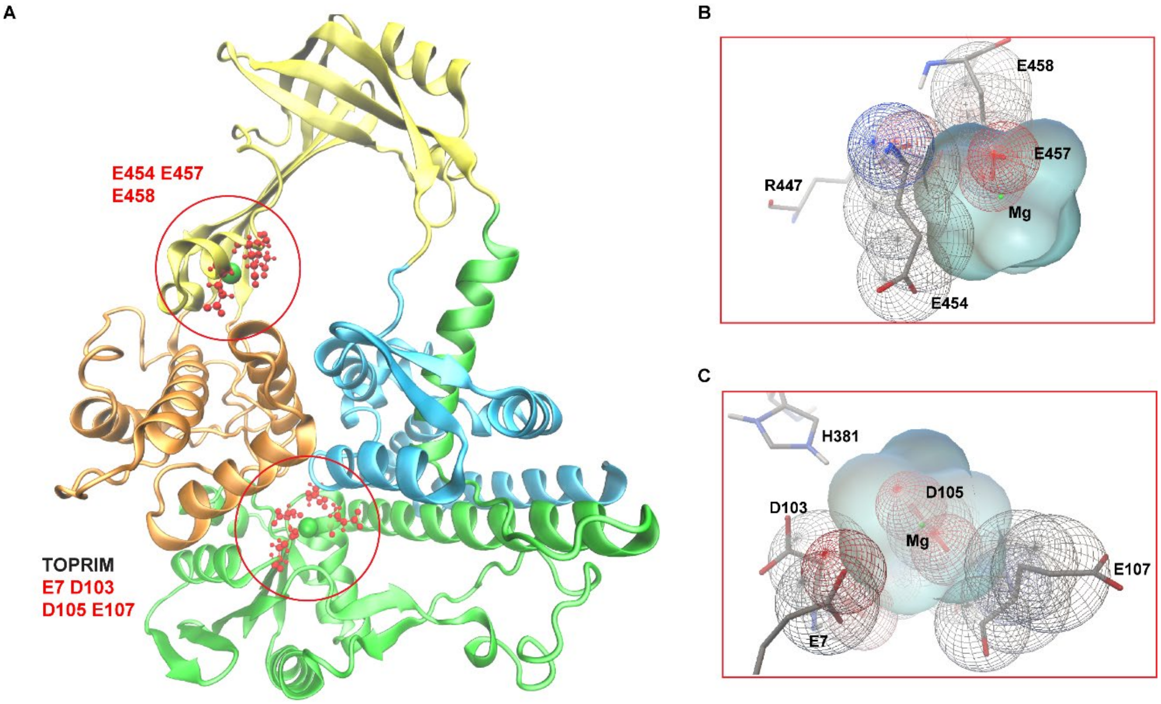

Supplementary Fig. 7. A. Divalent metal binding site in *ec*topo3 formed by the acidic triad: E454, E457, and E458 predicated by BioMetAll [11]. The conserved magnesium binding site within the TOPRIM domain was also predicted as a potential divalent metal binding site. The bound metal ions are indicated as green spheres and the residues forming the acidic triads are represented in red ball and stick configurations. The structure of *ec*topo3 was obtained via homology modelling based on PDB 2O19 [12,13]. **B.** Hydrated magnesium ion as a ligand was used for docking to the noncanonical metal binding site of *ec*topo3 using AutoDockTool and AutoDock4.2[7] (Supplementary Note). The hydrated state of a magnesium ion is represented with a magnesium ion (green ball) surrounded by 6 water molecules (blue shading) forming a first hydration cell. The closely interacting atoms of the residues are depicted as wire spheres. The estimated free energy of binding to the noncanonical divalent metal binding site is -6.8 kcal/mol (K_D=10.3 μM). **C.** The magnesium ion docking was also tested for the TOPRIM site with an estimated free energy of binding of -7.51 kcal/mol (K_D = 3.13 μM).

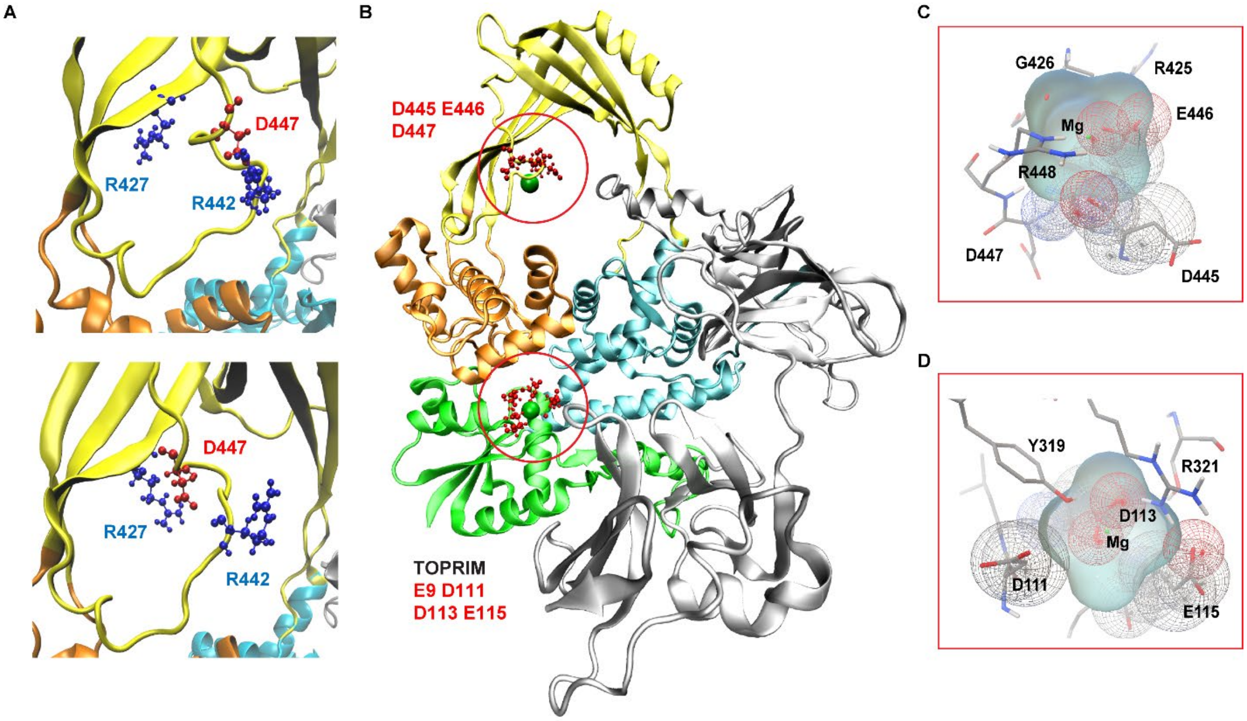

Supplementary Fig. 8. *ec*topo1 and *ec*topo3 share a similar divalent metal binding site that overlaps competing inter- and intra-domain salt bridges. A. The structure of *ec*topo1 was obtained via homology modelling based on PDB 4RUL[13,14]. The simulations were done similarly to those for ectopo3 (methods). The CA of the catalytic tyrosine, Y319 was chosen as SMD atoms. Harmonic constraints (50 kcal/mol), to counteract the applied force, were added to CA atoms of protein residues proposed to stabilize bound DNA (111, 113, and 168). In *ec*topo1, the acidic residue D447 can form a salt-bridge with R442 (top) or with R427 (bottom) that can influence domain III movement in an analogous manner as in *ec*topo3. **B.** The acidic triad including residue D447 was predicted as a potential Mg binding site by BioMetAll[11]. The conserved magnesium binding domain, TOPRIM (E9, D111, D113, and E115) was also predicted as a potential metal binding site for *ec*topo1. Bound metal ions are indicated as green spheres and the residues forming the acidic triads are represented in red ball and stick configurations. Despite the overall similarities in the divalent metal binding and competing salt bridge topology between *ec*topo1 and *ec*topo3, the residues and the structural arrangement in *ec*topo1 differ slightly from those in *ec*topo3. In contrast to the interdomain salt-bridge (domain II-III) that likely hinders full opening of the protein-gate in *ec*topo3, *ec*topo1 residue D447, part of the Mg binding acidic triad, interacts with R442, promoting bending of the β sheet linked to domain III, possibly leading to a partial opening of the protein-gate. Divalent metal binding at the acidic triad (445-447) could promote switching of the D447 salt bridge from R442 to R427 in a similar manner as proposed for *ec*topo3. **C.** Hydrated magnesium ion as a ligand was used for docking to the noncanonical divalent metal binding site of *ec*topo1 using AutoDockTool and AutoDock4.2[7] (Supplementary Note). The hydrated state of a magnesium ion is represented with a magnesium ion (green ball) surrounded by 6 water molecules (blue shading) forming a first hydration cell. The closely interacting atoms of the residues are depicted as wire spheres. The estimated free energy of binding to the noncanonical divalent metal binding site is -5.26 kcal/mol (K_D=138.7 µM). **D.** The magnesium ion docking was also tested for the TOPRIM site returning an estimated free energy of binding of -6.04 kcal/mol (K_D = 37.2 µM). The presence of bound DNA would likely enhance the magnesium binding affinity at the TOPRIM site, as both the DNA phosphate backbone and the acidic residues can coordinate magnesium as shown in the crystal structure of mycobacterial topo1[15].

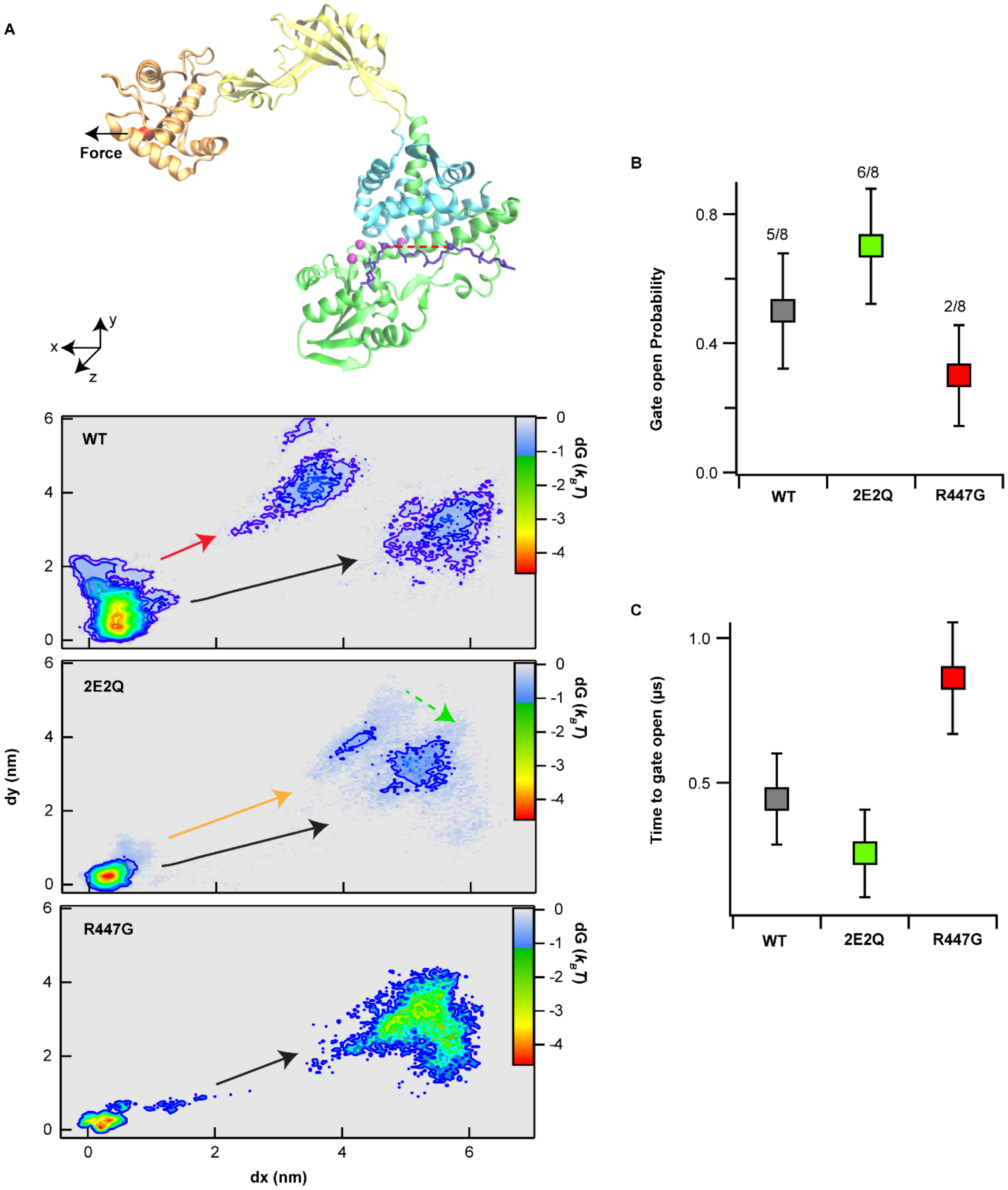

Supplementary Fig. 9. Constant force SMD with 2E2Q and R447G salt-bridge variants compared to WT *ec*topo3. A. 2-dimensional average energy landscapes of gate opening transitions for the three *ec*topo3 variants (obtained from 8 trajectories for each variant). Despite the use of 50 pN in SMD simulations, the extents of gate opening observed in MD simulations were similar among the replicates and also comparable to the experimentally measured values under similar, though lower forces. The *x*- and *y*- axes correspond to the center of mass motion of the catalytic tyrosine in domain III parallel and orthogonal to the direction of applied force, respectively. The SMD atom (Cα of catalytic tyrosine 328) is depicted as a red sphere, while the constraint atoms (Cα of residues 103, 105, and 165) are shown as magenta spheres. The vector between the two phosphates at positions 3 and 6 of the bound single-stranded DNA backbone (violet) is indicated by a red dashed line. This vector was used as a reference to

rotate the structure, aligning the bound DNA along the x-axis (inset). WT showed two distinctive transition pathways as discussed in Fig. 2A. The black arrow indicates a transition pathway similar to ″Sim1″, typified by rapid gate opening without intermediates. The red arrow corresponds to a transition pathway similar to ″Sim2″, which includes intermediate states without full gate opening. The 2E2Q trajectories did not exhibit a stable intermediate state preceding opening in contrast to WT, but some trajectories exhibited a slight orthogonal component leading to two different open states (black and orange arrows). The open state indicated by the orange arrow, which exhibited slightly less parallel and more orthogonal displacement, eventually converged to the other open state (green arrow). The R447G gate opening trajectories show the least orthogonal motion indicating that transient interactions between R447 and the acidic triad (454, 457, and 458) are likely responsible for orthogonal movement of domain III. The change in free energy (dG) was calculated from the position probability density, p(x,y) using $dG = -ln(p(x,y)) - G_{max}$. **B.** Gate opening transition probabilities for the three *ec*topo3 variants for 300 ns simulations (*N*= 8 trajectories for each enzyme). The average probabilities (WT: gray solid square; 2E2Q: green solid square; R447G red solid square) and the standard deviations were calculated based on Bayesian inference of the finite-duration simulation trajectories (Supplementary Note). **C.** Expected waiting times before gate opening. The waiting times (WT: gray solid square; 2E2Q: green solid square; R447G red solid square) were estimated based on the probability estimates from B given the 300 ns simulation time (Supplementary Note). The error bars correspond to the SEM.

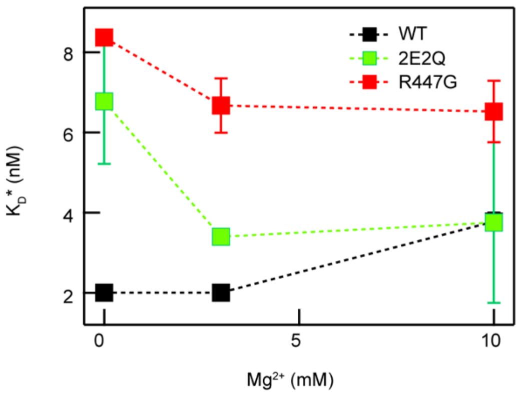

Supplementary Fig. 10. Single-stranded DNA binding affinity of WT, 2E2Q, and R447G as a function of Mg^{2+} concentration. Apparent K_d (K_d*) as a function of Mg concentration for the three *ec*topo3 variants determined from fluorescence anisotropy measurements (WT: black solid square; 2E2Q: green solid square; R447G red solid square). Error bars correspond to the standard deviation (SD). N = 3 independent measurements.

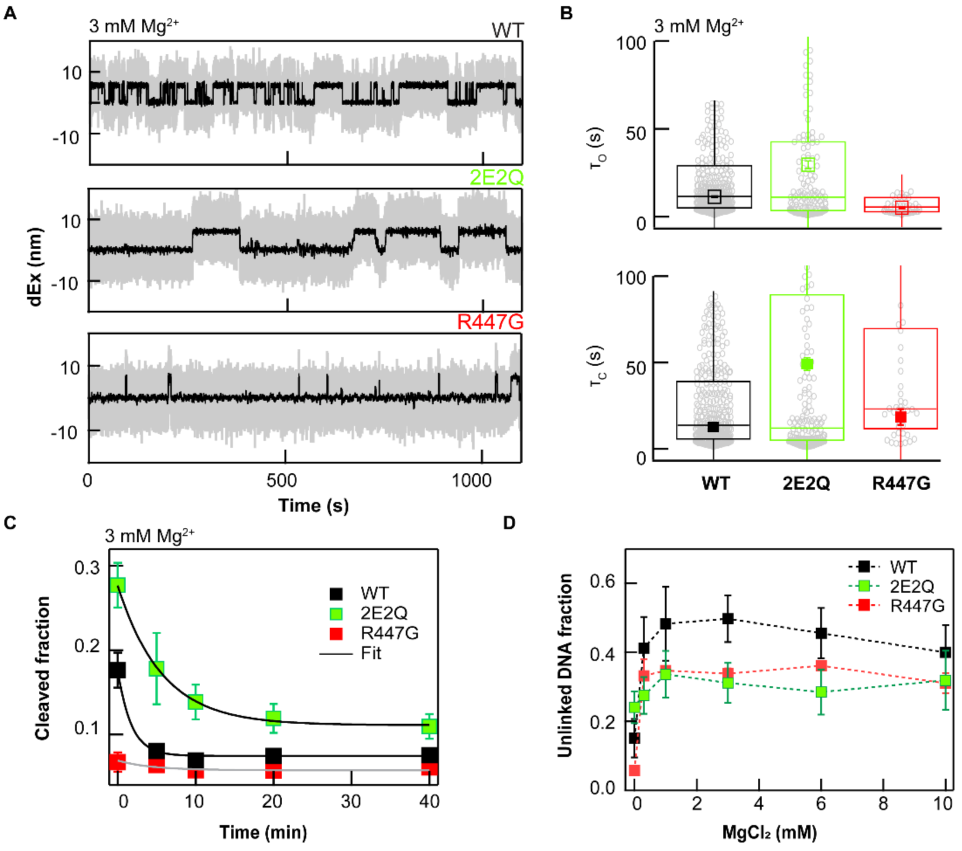

Supplementary Fig. 11. Salt-bridge mutations adversely affect Mg^{2+}-dependent topo3 catalytic activities. A. Gate dynamics trajectories of three *ec*topo3 variants at 3 mM Mg^{2+}. All three enzymes show full gate opening. However, 2E2Q gate opening exhibited a mixture of fast (comparable to WT) and slow dynamics whereas R447G displayed overall slower gate dynamics than WT. **B.** Box plots of open and closed state durations (τ_O and τ_C respectively) for the three variants. The horizontal lines within the boxes (black: WT; green: 2E2Q; red: R447G) indicate the median, whereas the overlayed rectangles (open for τ_O and close for τ_C; black: WT; green: 2E2Q; red (R447G) indicate the average value from single exponential fits with standard deviations as errors and the whiskers correspond to the minimum and maximum values. 2E2Q gate open and closed durations are broadly distributed, reflecting the mixture of fast and slow gate dynamics shown in **A**. Number of events for open (391) and closed (395) for WT; open (168) and closed (211) for 2E2Q; open (50) and closed (44) for R447G. Number of biological replicates: 8 (WT); 5 (2E2Q); 5 (R447G). **C.** Cleaved DNA fraction as a function of time for three *ec*topo3 variants fit with a cleavage-religation equilibrium function (Supplementary Note). R447G data was omitted from the analysis due to insufficient cleaved DNA. The rate of reduction in the cleaved fraction reflects religation and cleavage rates. Number of biological replicate: 6 (WT); 6 (2E2Q); 4 (R447G). Error bars indicate the standard error of the mean. **D.** Decatenation activities of the three variants as a function of Mg concentration. Deregulation of gate dynamics in 2E2Q adversely affects enzyme catalytic activity by reducing cleavage and religation rates resulting in inefficient decatenation. Furthermore, lower decatenation activity of R447G relative to WT indicates that Mg^{2+} alone was insufficient to fully compensate for the role of R447 in destabilizing the closed state. Number of biological replicates: 5 (WT); 6 (2E2Q); 3 (R447G). Error bars indicate the standard error of the mean.

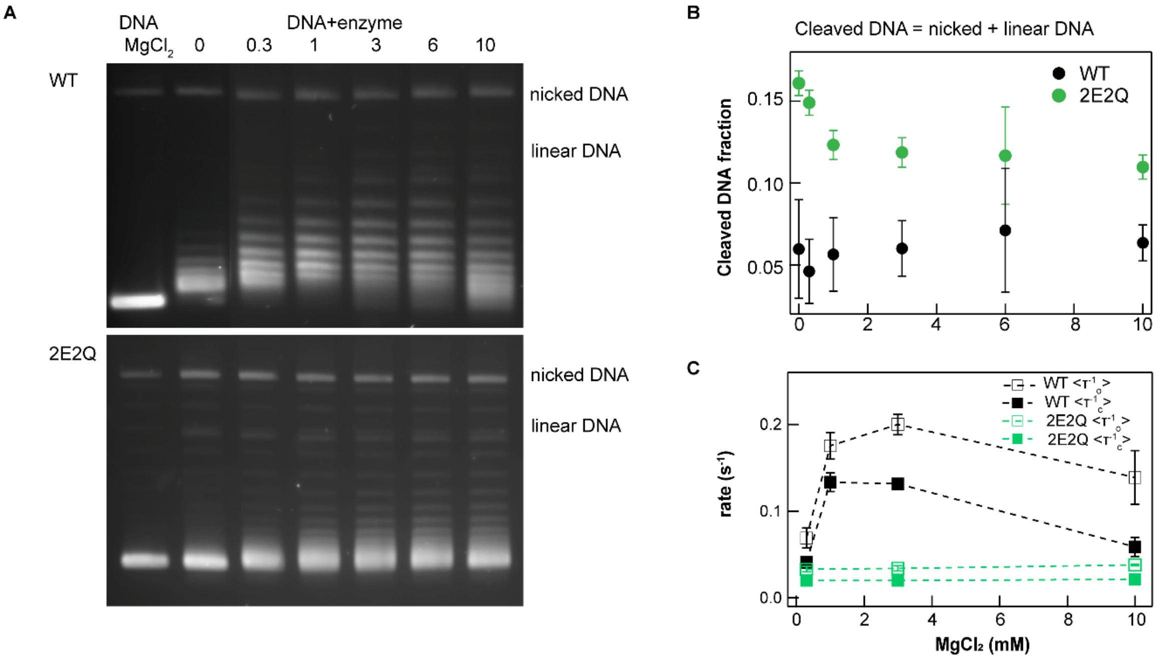

Supplementary Fig. 12. 2E2Q exhibits higher level of DNA cleavage compared to WT. A. DNA supercoil relaxation measurements of WT and 2E2Q visualized by agarose gel electrophoresis. 2E2Q showed a higher propensity for cleavage as evidenced by the higher fraction of nicked and linear bands in comparison to WT *ec*topo3. **B.** Cleaved DNA fraction (black solid circle: WT; green solid circle: 2E2Q) as a function of Mg concentration obtained from analysis of the gels in panel **A**. Number of biological replicates: 1 Error bars correspond to fitting errors. **C.** Comparison of the gate opening- and closing- kinetics between 2E2Q and WT as a function of Mg concentration. The rates were obtained from the analysis of the single molecule gate dynamic measurements (Fig. 2 and Supplementary Fig. 11). Despite the higher cleavage, the average rates for the closed as well as open states ($<\tau_O^{-1}>$ and $<\tau_C^{-1}>$) of 2E2Q were lower than WT and largely insensitive to Mg^{2+} concentration indicating that the two acidic residues are critical for Mg^{2+}-dependent gate dynamics. Black open and close squares represent WT $<\tau_O^{-1}>$ and $<\tau_C^{-1}>$. Green open and close squares represent 2E2Q $<\tau_O^{-1}>$ and $<\tau_C^{-1}>$. All error bars correspond to fitting errors.

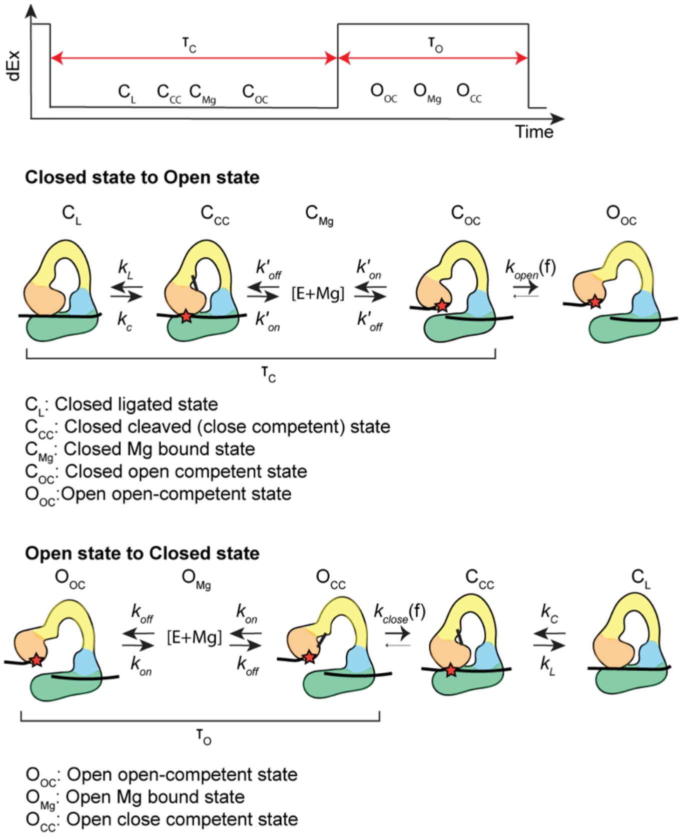

Supplementary Fig. 13. Mg-dependent salt-bridge switch kinetic model. The experimentally observed gate motion was modeled as a two-state telegraphic signal in which the lower signal represents the closed state, and the higher signal represents the open state. The open and closed state lifetimes are each comprised of several sequential rates. Expressions for the open and closed state lifetimes based on this kinetic scheme are described above.

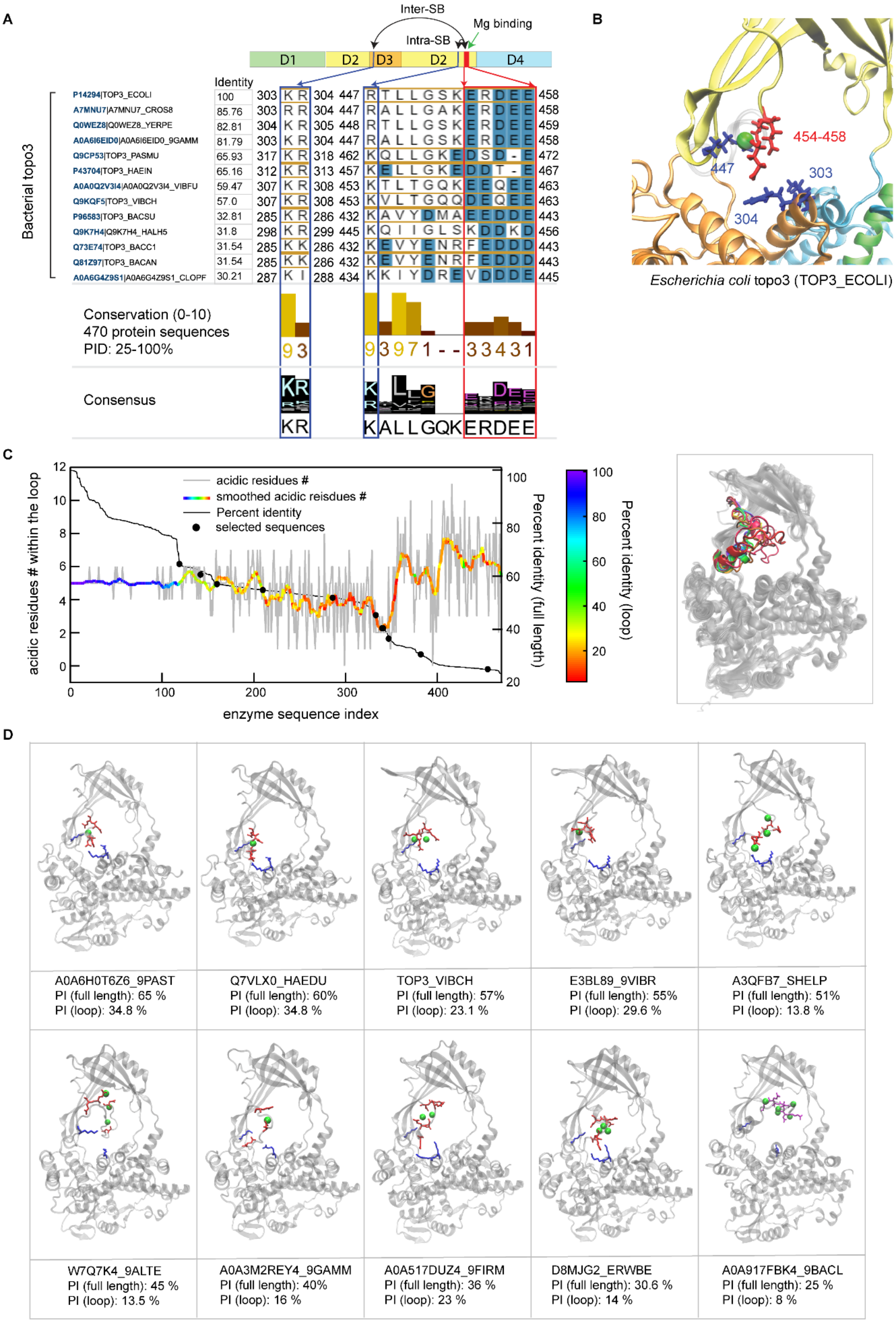
A
Inter-SB
Intra-SB
Mg binding
D1
D2
D3
D2
D4
Identity
Bacterial topo3
Conservation (0-10)
470 protein sequences
PID: 25-100%
Consensus
KR
KALLGQKERDEE
B
454-458
447
303
304
Escherichia coli topo3 (TOP3_ECOLI)
C
acidic residues #
smoothed acidic reisdues #
Percent identity
selected sequences
acidic residues # within the loop
Percent identity (full length)
Percent identity (loop)
enzyme sequence index
D
A0A6H0T6Z6_9PAST
PI (full length): 65 %
PI (loop): 34.8 %
Q7VLX0_HAEDU
PI (full length): 60%
PI (loop): 34.8 %
TOP3_VIBCH
PI (full length): 57%
PI (loop): 23.1 %
E3BL89_9VIBR
PI (full length): 55%
PI (loop): 29.6 %
A3QFB7_SHELP
PI (full length): 51%
PI (loop): 13.8 %
W7Q7K4_9ALTE
PI (full length): 45 %
PI (loop): 13.5 %
A0A3M2REY4_9GAMM
PI (full length): 40%
PI (loop): 16 %
A0A517DUZ4_9FIRM
PI (full length): 36 %
PI (loop): 23 %
D8MJG2_ERWBE
PI (full length): 30.6 %
PI (loop): 14 %
A0A917FBK4_9BACL
PI (full length): 25 %
PI (loop): 8 %

Supplementary Fig. 14. Sequence conservation of overlapping salt-bridge and magnesium binding site architecture among bacterial topoisomerase 3 enzymes. **A.** Sequence alignment of bacterial topo3 revealed that the Mg^{2+} binding-site residues (red lines and outlines) and inter- and intra-domain salt-bridge forming residues (blue lines and outlines) are highly conserved, suggesting that they share the similar Mg-dependent protein gate regulation. Representative examples of bacterial topo3 sequence alignments are shown on top. The conservation level and consensus sequence, from the analysis of 470 sequences using the Muscle tool in Jalview[9,10], corresponding to the salt-bridge forming and Mg^{2+} binding-site resides is shown below. **B.** Representative structure of E. *coli* topo3 indicates the arrangement of the two basic residues and multiple acidic residues involved in inter- and intra-domain salt-bridges and divalent metal binding. Green sphere indicates a metal ion. The structure of *ec*topo3 was obtained via homology modelling based on PDB 2O19. [12,13] **C.** The numbers of acidic residues within each loop (13-54 amino acids) of 470 different bacterial topoisomerase 3 sequences (left axis) were overlayed with the percent identity to *ec*topo3 (right). The smoothed number of acidic residues (10 point average) was colored coded based on the percent identity of the loop sequence referenced to *ec*topo3. *Inset*: The varying lengths and structures of the loops from different bacterial topoisomerase 3 were predicted by Alphafold[16]. **D.** The structures of 10 selected sequences (indicated in C) were tested with Biometall for divalent metal binding within each loop[11]. The acidic residues that are aligned to those of *ec*topo3 are represented with red sticks while those that are not aligned are represented with magenta sticks. The inter- and intra-domain salt-bridge forming basic residues are represented with blue sticks. Green sphere represents a divalent metal ion.

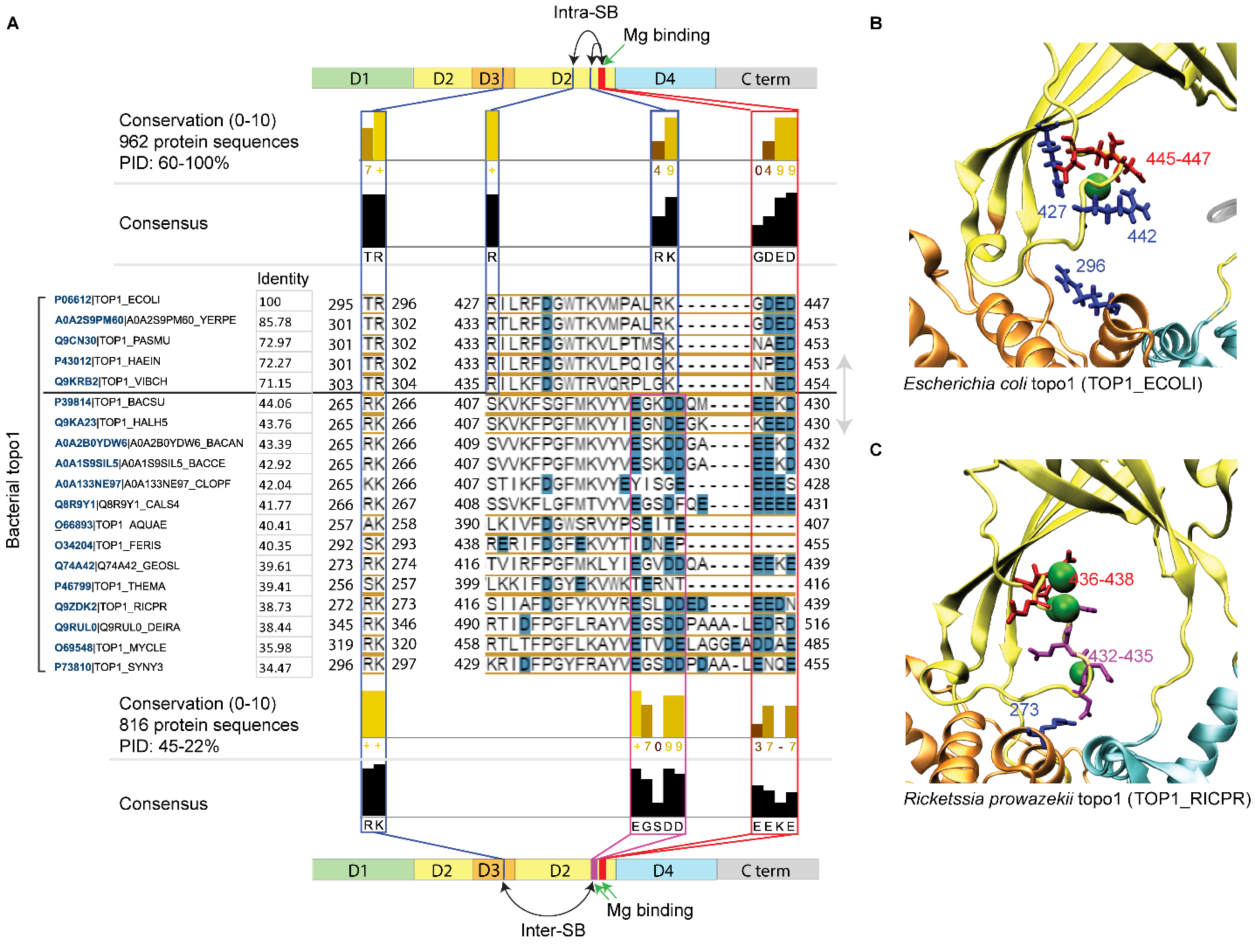

Supplementary Fig. 15. Sequence and putative functional conservation of salt-bridge and Mg binding site architecture among bacterial topoisomerase 1 enzymes. A. Sequence alignment of bacterial topo1 showed that enzymes closely related to *E. coli* topo1 (percent identity: PID >60%) have a similar arrangement of the salt-bridge forming (blue lines and outlines) and Mg binding residues (red lines and outlines) in domain II, whereas those with lower PID (22-45%) possess an inter-domain salt-bridge arrangement similar to *ec*topo3 (Supplementary Fig. 14) with an additional acidic residue patch (magenta) in domain II. The multiple acidic regions (magenta and red) possibly accommodate multiple magnesium ions. Representative bacterial topo1 sequence alignments (middle). The conservation level and consensus sequence corresponding to the salt-bridge forming and divalent metal binding-site residues for PID > 60% (962 sequences) are shown on the top and for lower PID (816 sequences) on the bottom[9,10]. **B.** Representative structure of *E. coli* topo1 indicates the arrangement of the two basic residues and multiple acidic residues involved in the intra-domain salt-bridge and Mg binding. Green sphere indicates a divalent metal ion. The structure of *ec*topo1 was obtained via homology modelling based on PDB 4RUL [13,14]. **C.** Representative structure of *Ricketssia prowazekii* topo1 from the AlphaFold protein structure database[16] as an example of the alternative topo1 sequence and architecture. Green sphere indicates a divalent metal ion.

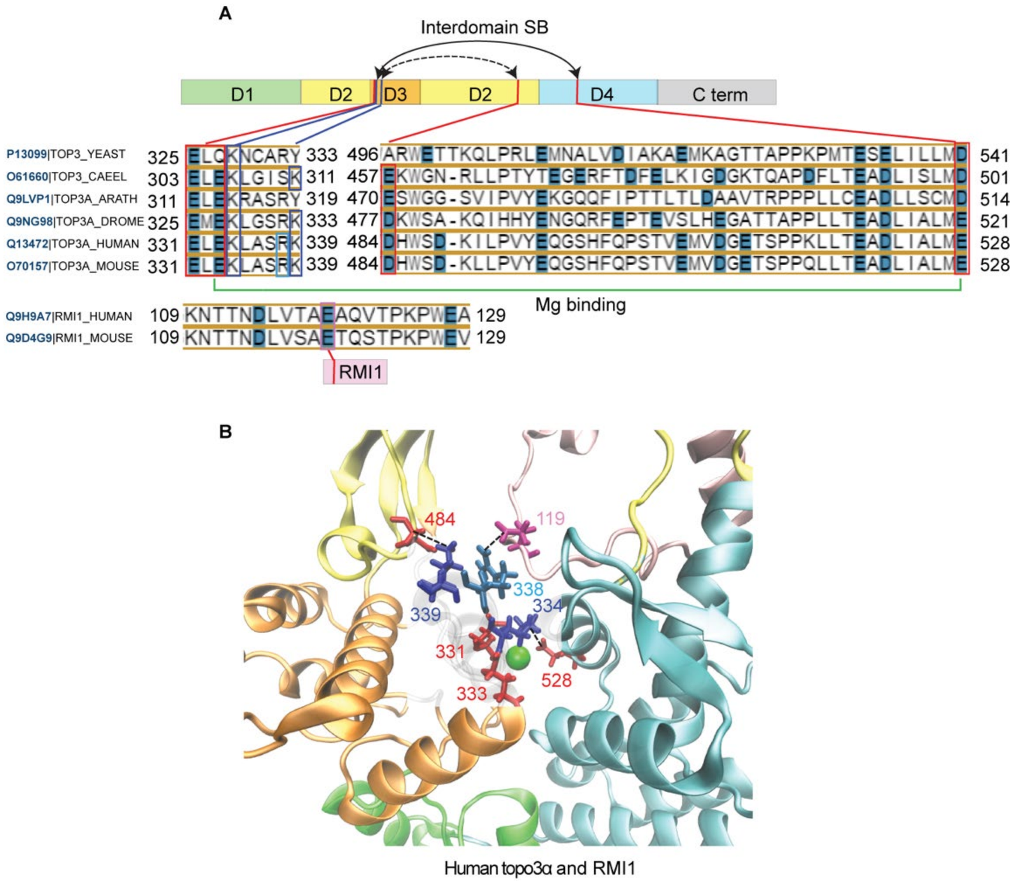

Supplementary Fig. 16. Functional conservation of Salt-bridge and Mg binding site architecture in human topoisomerase 3α-RMI1 complex. A. Sequence alignment of multiple eukaryotic topo3 revealed residues that could functionally replicate the Mg-dependent protein gate regulation though alterations in salt-bridge topology. **B.** The conserved residues contributing to the putative Mg-dependent salt-bridge architecture in **A** and a divalent metal (green sphere) are indicated in the expanded view of human topoisomerase 3α (htopo3α) and RMI1 complex structure (based on PDB 4CGY). The salt-bridge interaction between K334 (Domain 3) and E528 (Domain 4) hinders gate-opening that can be disrupted by Mg binding coordinated by (E331, E333, and E528). One of the conserved basic residues in domain III of htopo3α (K339) maintains an inter-domain salt-bridge with D484, while the other residue (R338) interacts with an acidic residue (E119) in the RMI1 insertion loop [17].

References

1 Schindelin, J. *et al.* Fiji: an open-source platform for biological-image analysis. *Nature Methods* **9**, 676–682 (2012). https://doi.org/10.1038/nmeth.2019

2 Donovan, T. & Mickey, R. M. *Bayesian Statistics for Beginners: a step-by-step approach*. (Oxford University Press, 2019).

3 Seol, Y., Zhang, H., Pommier, Y. & Neuman, K. C. A kinetic clutch governs religation by type IB topoisomerases and determines camptothecin sensitivity. *Proceedings of the National Academy of Sciences of the United States of America* **109**, 16125–16130 (2012). https://doi.org/10.1073/pnas.1206480109

4 Shaevitz, J. W., Block, S. M. & Schnitzer, M. J. Statistical Kinetics of Macromolecular Dynamics. *Biophysical Journal* **89**, 2277–2285 (2005). https://doi.org/https://doi.org/10.1529/biophysj.105.064295

5 Persson, I. Structure and size of complete hydration shells of metal ions and inorganic anions in aqueous solution. *Dalton Transactions* **53**, 15517–15538 (2024). https://doi.org/10.1039/D4DT01449A

6 Forli, S. & Olson, A. J. A Force Field with Discrete Displaceable Waters and Desolvation Entropy for Hydrated Ligand Docking. *Journal of Medicinal Chemistry* **55**, 623–638 (2012). https://doi.org/10.1021/jm2005145

7 Morris, G. M. *et al.* AutoDock4 and AutoDockTools4: Automated docking with selective receptor flexibility. *Journal of Computational Chemistry* **30**, 2785–2791 (2009). https://doi.org/https://doi.org/10.1002/jcc.21256

8 Consortium, T. U. UniProt: the Universal Protein Knowledgebase in 2023. *Nucleic Acids Research* **51**, D523–D531 (2022). https://doi.org/10.1093/nar/gkac1052

9 Edgar, R. C. Muscle5: High-accuracy alignment ensembles enable unbiased assessments of sequence homology and phylogeny. *Nature Communications* **13**, 6968 (2022). https://doi.org/10.1038/s41467-022-34630-w

10 Waterhouse, A. M., Procter, J. B., Martin, D. M. A., Clamp, M. & Barton, G. J. Jalview Version 2—a multiple sequence alignment editor and analysis workbench. *Bioinformatics* **25**, 1189–1191 (2009). https://doi.org/10.1093/bioinformatics/btp033

11 Sánchez-Aparicio, J.-E. *et al.* BioMetAll: Identifying Metal-Binding Sites in Proteins from Backbone Preorganization. *Journal of Chemical Information and Modeling* **61**, 311–323 (2021). https://doi.org/10.1021/acs.jcim.0c00827

12 Changela, A., DiGate, R. J. & Mondragón, A. Structural Studies of E. coli Topoisomerase III-DNA Complexes Reveal A Novel Type IA Topoisomerase-DNA Conformational Intermediate. *Journal of molecular biology* **368**, 105–118 (2007). https://doi.org/10.1016/j.jmb.2007.01.065

13 Waterhouse, A. *et al.* SWISS-MODEL: homology modelling of protein structures and complexes. *Nucleic Acids Res* **46**, W296–w303 (2018). https://doi.org/10.1093/nar/gky427

14 Tan, K. *et al.* Structural basis for suppression of hypernegative DNA supercoiling by E. coli topoisomerase I. *Nucleic Acids Research* **43**, 11031–11046 (2015). https://doi.org/10.1093/nar/gkv1073

15 Cao, N., Tan, K., Annamalai, T., Joachimiak, A. & Tse-Dinh, Y.-C. Investigating mycobacterial topoisomerase I mechanism from the analysis of metal and DNA substrate interactions at the active site. *Nucleic Acids Research* **46**, 7296–7308 (2018). https://doi.org/10.1093/nar/gky492

16 Varadi, M. *et al.* AlphaFold Protein Structure Database in 2024: providing structure coverage for over 214 million protein sequences. *Nucleic Acids Research* **52**, D368–D375 (2023). https://doi.org/10.1093/nar/gkad1011

17 Bocquet, N. *et al.* Structural and mechanistic insight into Holliday-junction dissolution by Topoisomerase IIIα and RMI1. *Nature Structural & Molecular Biology* **21**, 261–268 (2014). https://doi.org/10.1038/nsmb.2775

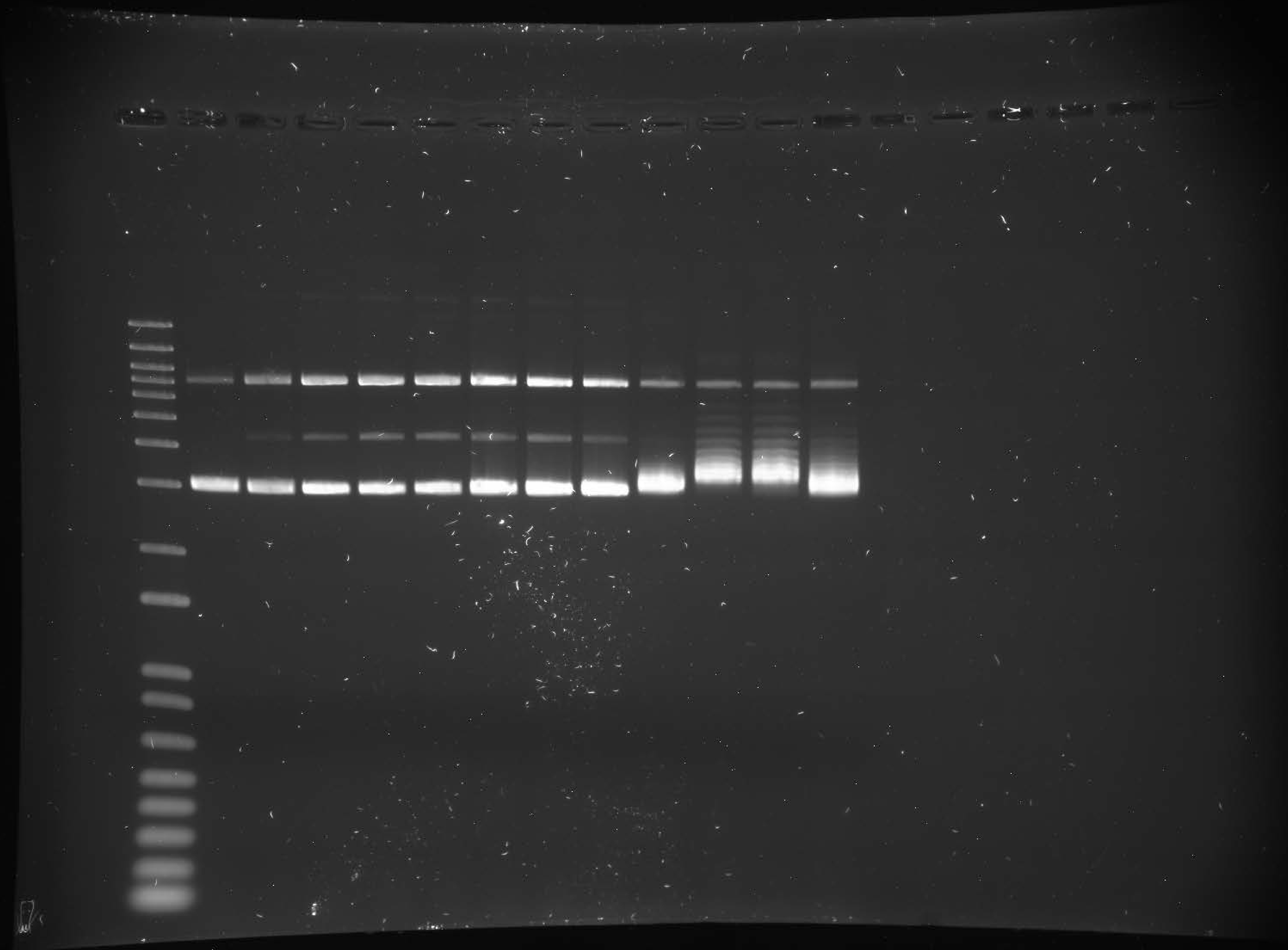

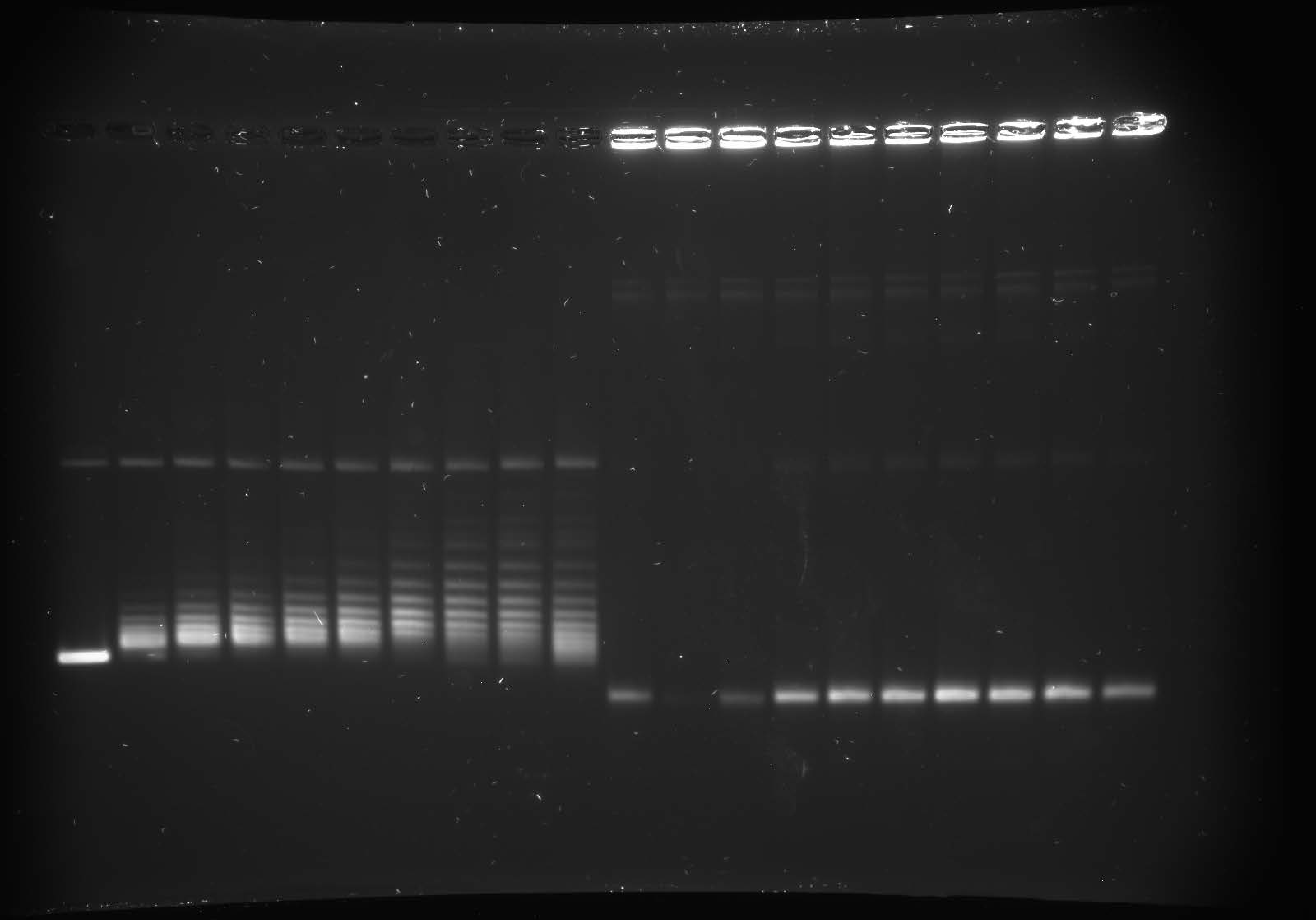

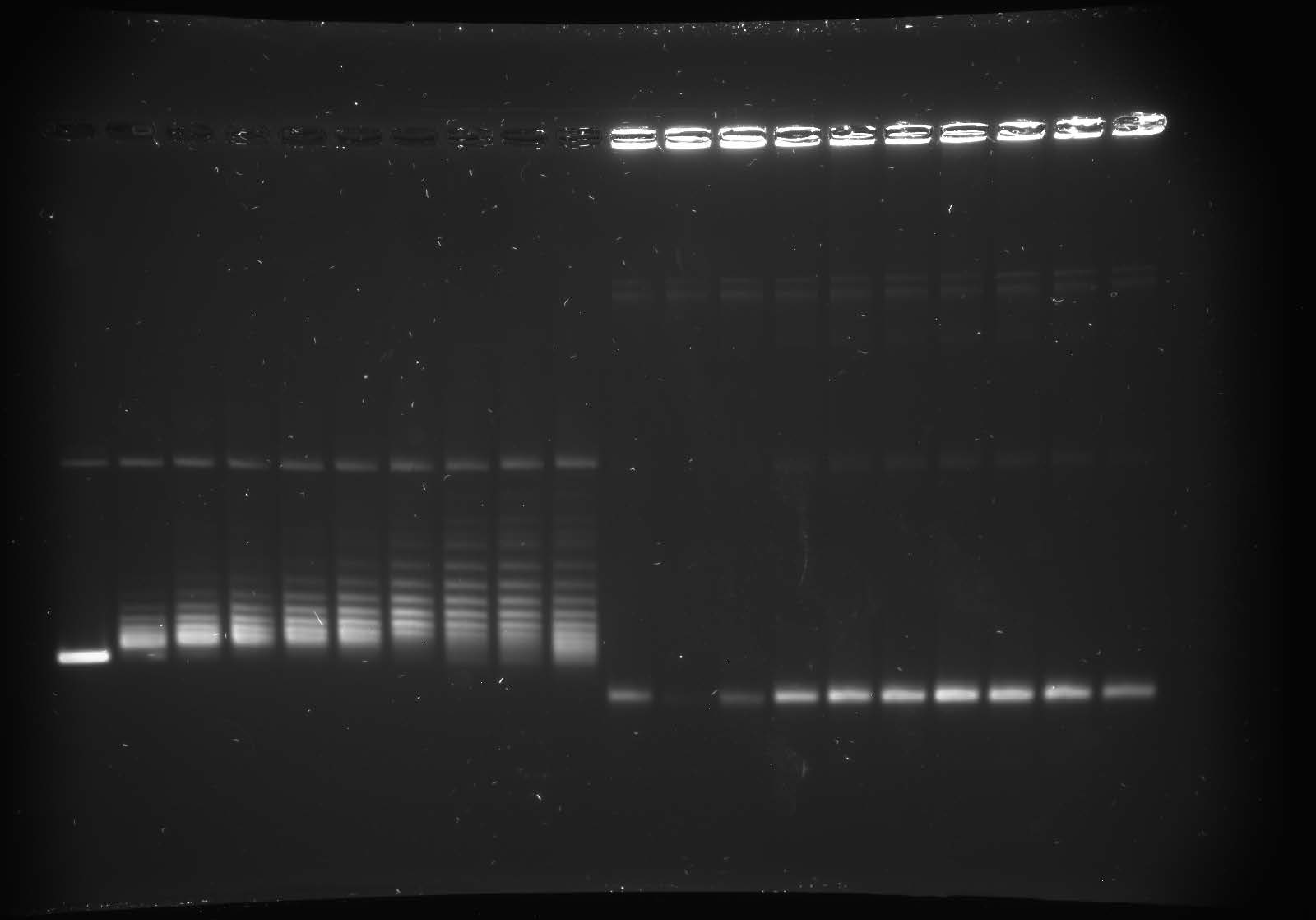

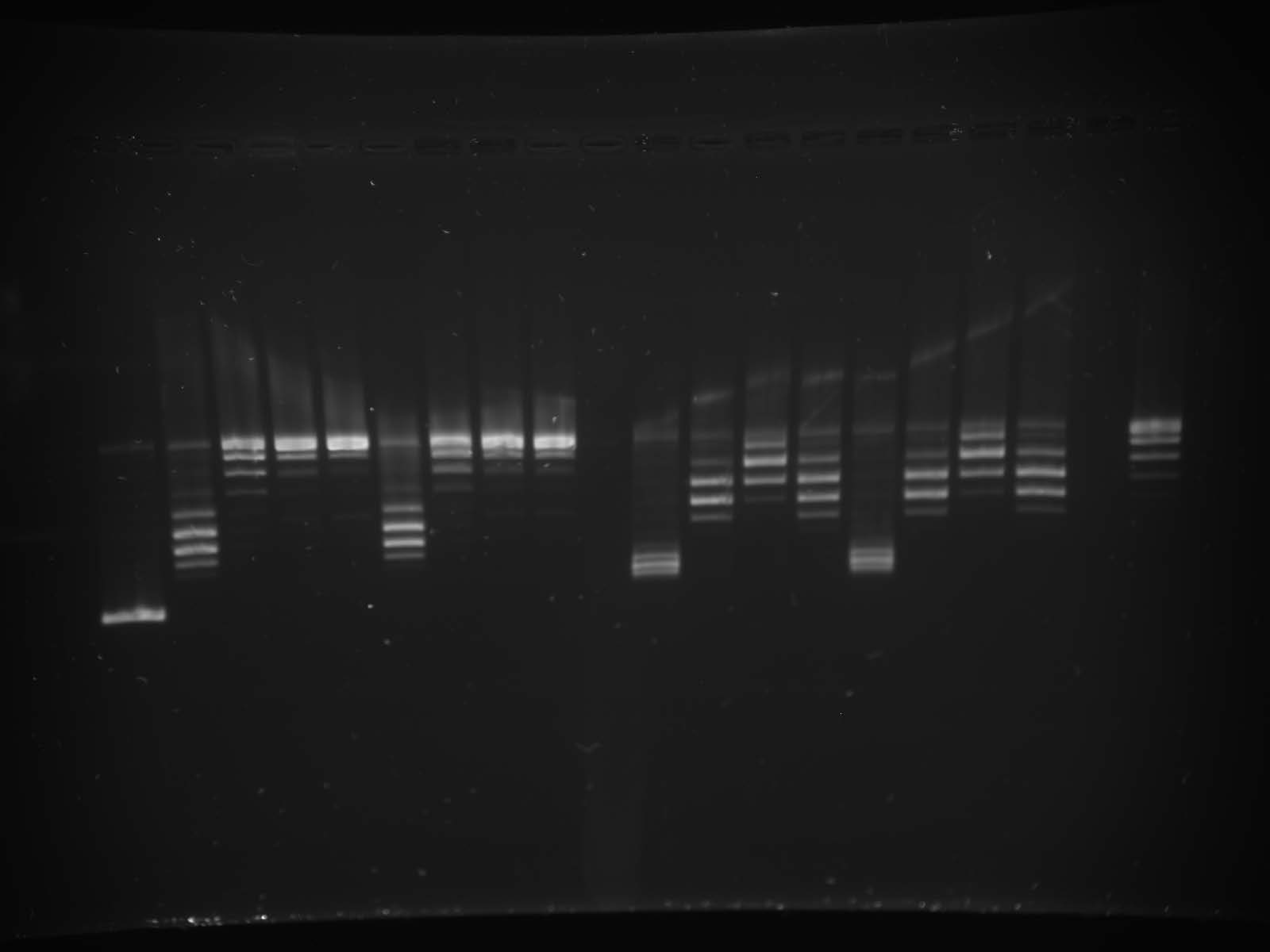

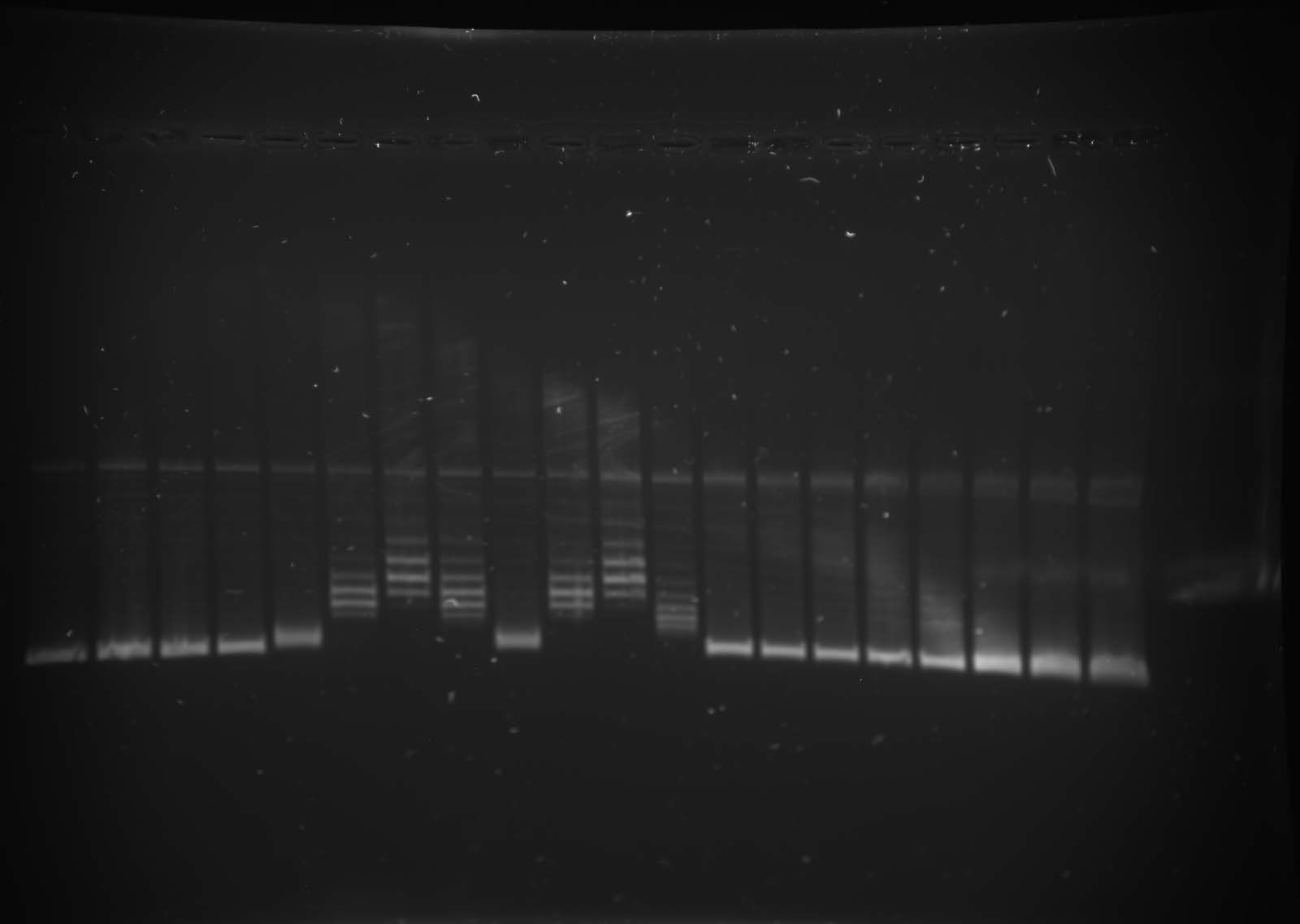

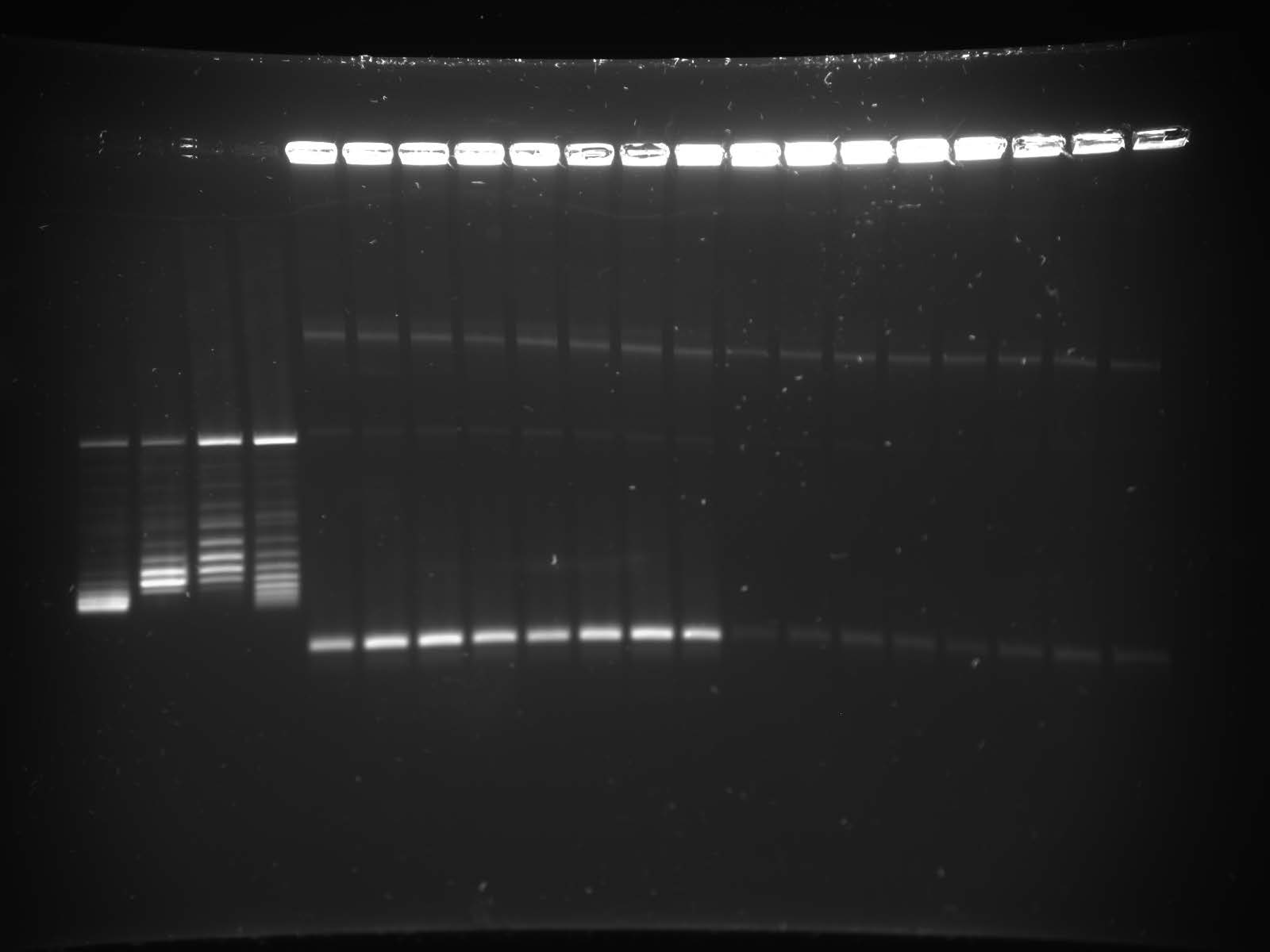

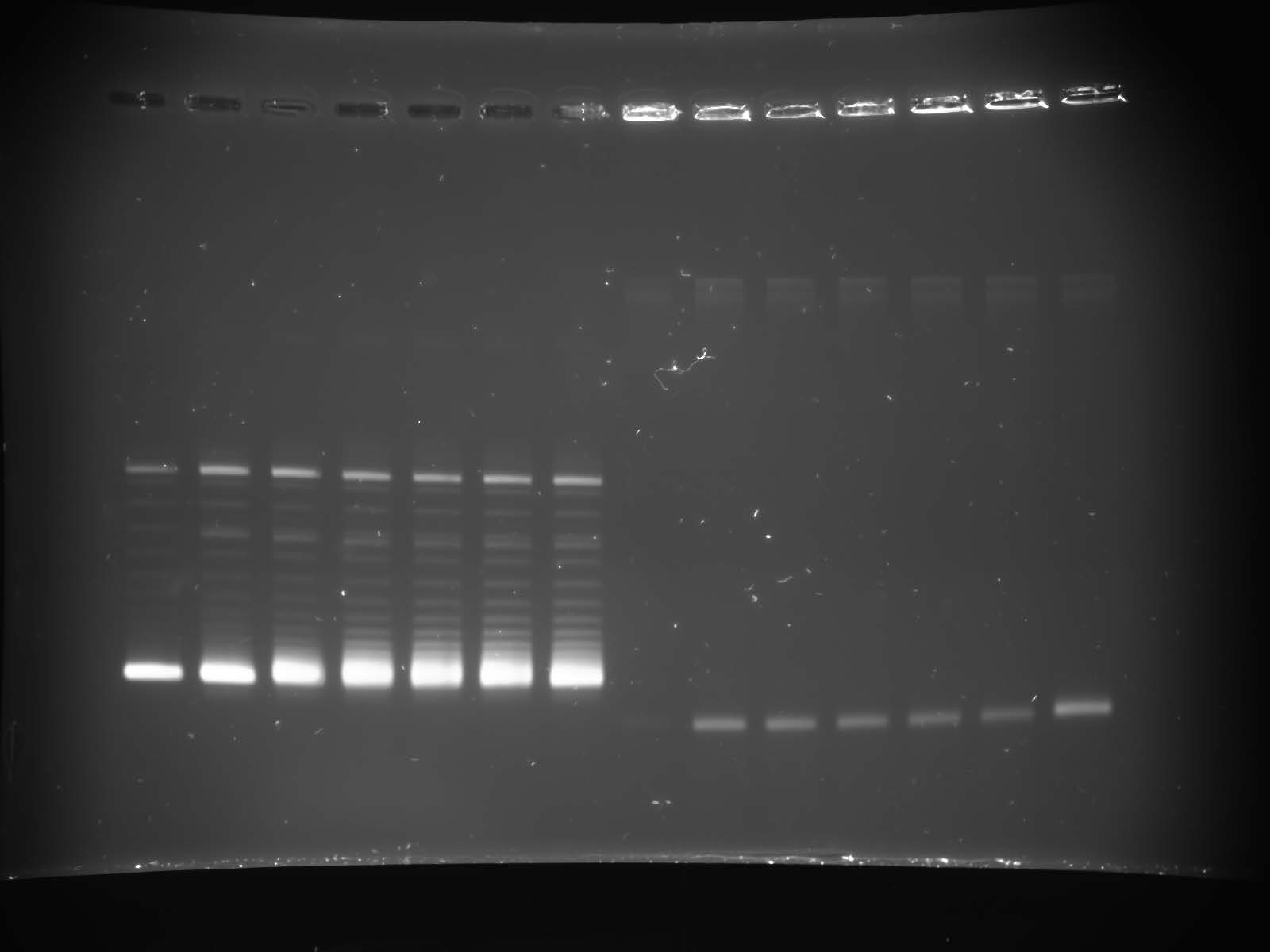

Supplement: Supplementary file 1 — Supplementary Information [file 41467_2026_72556_MOESM1_ESM.pdf]
