# Supplementary material for: Networked salt-bridges mediate magnesium-dependent conformational dynamics and functional regulation in type IA topoisomerases
Source: Nat Commun. 2026 Apr 30;17:5907. doi: 10.1038/s41467-026-72556-9 (PMC13338365; doi:10.1038/s41467-026-72556-9)
Supplement: Supplementary file 2 — Reporting Summary [file 41467_2026_72556_MOESM2_ESM.pdf]

nature portfolio

Corresponding author(s): Keir Neuman

Last updated by author(s): 01/26/2026

Reporting Summary

Nature Portfolio wishes to improve the reproducibility of the work that we publish. This form provides structure for consistency and transparency in reporting. For further information on Nature Portfolio policies, see our Editorial Policies and the Editorial Policy Checklist.

Statistics

For all statistical analyses, confirm that the following items are present in the figure legend, table legend, main text, or Methods section.

n/a	Confirmed	
☐	☒	The exact sample size (*n*) for each experimental group/condition, given as a discrete number and unit of measurement
☐	☒	A statement on whether measurements were taken from distinct samples or whether the same sample was measured repeatedly
☒	☐	The statistical test(s) used AND whether they are one- or two-sided<br>*Only common tests should be described solely by name; describe more complex techniques in the Methods section.*
☒	☐	A description of all covariates tested
☒	☐	A description of any assumptions or corrections, such as tests of normality and adjustment for multiple comparisons
☐	☒	A full description of the statistical parameters including central tendency (e.g. means) or other basic estimates (e.g. regression coefficient) AND variation (e.g. standard deviation) or associated estimates of uncertainty (e.g. confidence intervals)
☒	☐	For null hypothesis testing, the test statistic (e.g. *F*, *t*, *r*) with confidence intervals, effect sizes, degrees of freedom and *P* value noted<br>*Give P values as exact values whenever suitable.*
☐	☒	For Bayesian analysis, information on the choice of priors and Markov chain Monte Carlo settings
☒	☐	For hierarchical and complex designs, identification of the appropriate level for tests and full reporting of outcomes
☒	☐	Estimates of effect sizes (e.g. Cohen's *d*, Pearson's *r*), indicating how they were calculated

Our web collection on statistics for biologists contains articles on many of the points above.

Software and code

Policy information about availability of computer code

Data collection	We used commercially available software LabVIEW2018 (single molecule data collection), NAMD2.0 (molecular dynamic simulations), BioMetAll (metal binding prediction with protein), AutoDock4.27 (magnesium binding to protein) for data collection.
Data analysis	We used either commercially available or open source softwares for data analysis: Igor 9(single molecule data analysis), VMD 1.9.3 and ProDy (molecular dynamic data analysis), Fiji (gel image analysis), ICON (single molecule gate motion analysis), AutoDockTool (AutoDock4.27 docking result viewer), and JalView (sequence alignment tool).

For manuscripts utilizing custom algorithms or software that are central to the research but not yet described in published literature, software must be made available to editors and reviewers. We strongly encourage code deposition in a community repository (e.g. GitHub). See the Nature Portfolio guidelines for submitting code & software for further information.

Data

Policy information about availability of data

All manuscripts must include a data availability statement. This statement should provide the following information, where applicable:

- Accession codes, unique identifiers, or web links for publicly available datasets
- A description of any restrictions on data availability
- For clinical datasets or third party data, please ensure that the statement adheres to our policy

All data that support the findings of this study presented in the manuscript and supplementary information file are available in Figshare at 10.25444/nhlbi.31127188.

Research involving human participants, their data, or biological material

Policy information about studies with human participants or human data. See also policy information about sex, gender (identity/presentation), and sexual orientation and race, ethnicity and racism.

Reporting on sex and gender	N/A
Reporting on race, ethnicity, or other socially relevant groupings	N/A
Population characteristics	N/A
Recruitment	N/A
Ethics oversight	N/A

Note that full information on the approval of the study protocol must also be provided in the manuscript.

Field-specific reporting

Please select the one below that is the best fit for your research. If you are not sure, read the appropriate sections before making your selection.

[x] Life sciences [ ] Behavioural & social sciences [ ] Ecological, evolutionary & environmental sciences

For a reference copy of the document with all sections, see nature.com/documents/nr-reporting-summary-flat.pdf

Life sciences study design

All studies must disclose on these points even when the disclosure is negative.

Sample size	No statistical method was used to predetermine sample sizes. No sample size calculation was done as 3-5 biological replicates were sufficient to obtain statistical average, variance, and uncertainty that provided sufficient statistical support for the observed differences.
Data exclusions	No data were excluded from the analysis.
Replication	Number of replicates are indicated in the legends. All single molecule experiments contain at least three biological replicates from different sample preparations. All replicates were independent from each other and all attemps at replication were successful.
Randomization	Randomization was not relevant to the study. There was no bias in selection or allocations of samples between experiments. Data acquisition was automated and analysis was software controlled.
Blinding	Blinding was not relevant to the study as there were no subjective measures. The same approaches were applied uniformly to the data from different conditions. Additionally, appropriate negative controls were included in all experiments. Data acquisition was automated and analysis was software controlled.

Reporting for specific materials, systems and methods

We require information from authors about some types of materials, experimental systems and methods used in many studies. Here, indicate whether each material, system or method listed is relevant to your study. If you are not sure if a list item applies to your research, read the appropriate section before selecting a response.

Materials & experimental systems

n/a	Involved in the study	
[ ]	[x]	Antibodies
[x]	[ ]	Eukaryotic cell lines
[x]	[ ]	Palaeontology and archaeology
[x]	[ ]	Animals and other organisms
[x]	[ ]	Clinical data
[x]	[ ]	Dual use research of concern
[x]	[ ]	Plants

Methods

n/a	Involved in the study	
[x]	[ ]	ChIP-seq
[x]	[ ]	Flow cytometry
[x]	[ ]	MRI-based neuroimaging

Antibodies

Antibodies used	Anti-digoxigenin (Roche, 11333089001) used for attaching DNA to glass surface described in the Method.
Validation	Validation statement for the antibody can be found through a following link:

https://www.sigmaaldrich.com/deepweb/assets/sigmaaldrich/product/documents/241/188/11333089001.pdf

Plants

Seed stocks	N/A
Novel plant genotypes	N/A
Authentication	N/A
