# Supplementary figures and images for: Networked salt-bridges mediate magnesium-dependent conformational dynamics and functional regulation in type IA topoisomerases
Source: Nat Commun. 2026 Apr 30;17:5907. doi: 10.1038/s41467-026-72556-9 (PMC13338365; doi:10.1038/s41467-026-72556-9)

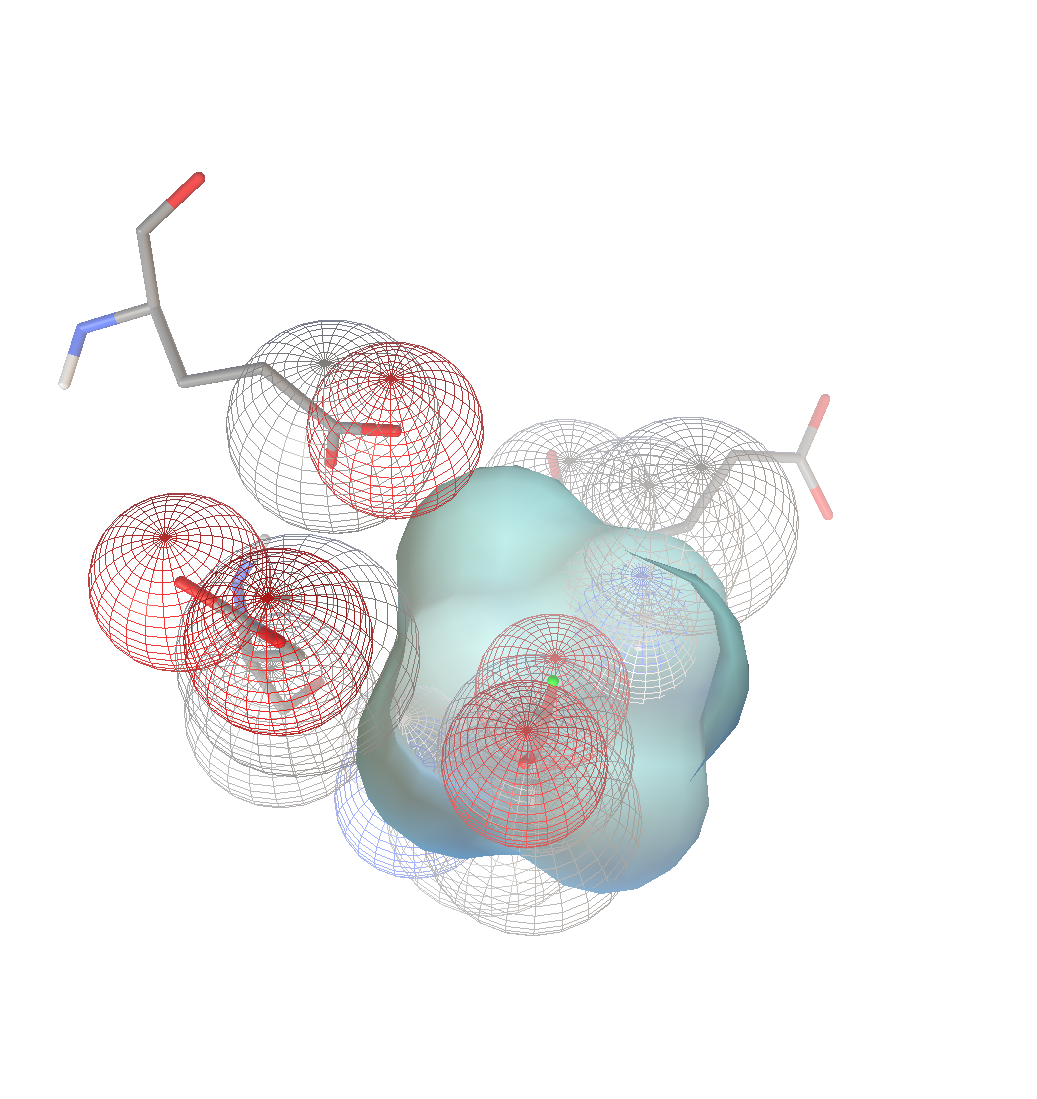

Supplement: Supplementary file 6 — Source Data 3 [file 41467_2026_72556_MOESM6_ESM.zip › source data supplementary fig 7BC/t3_TOPRIM/mg2_t4/IDNZ_mg_t3_t4_m4.tif]

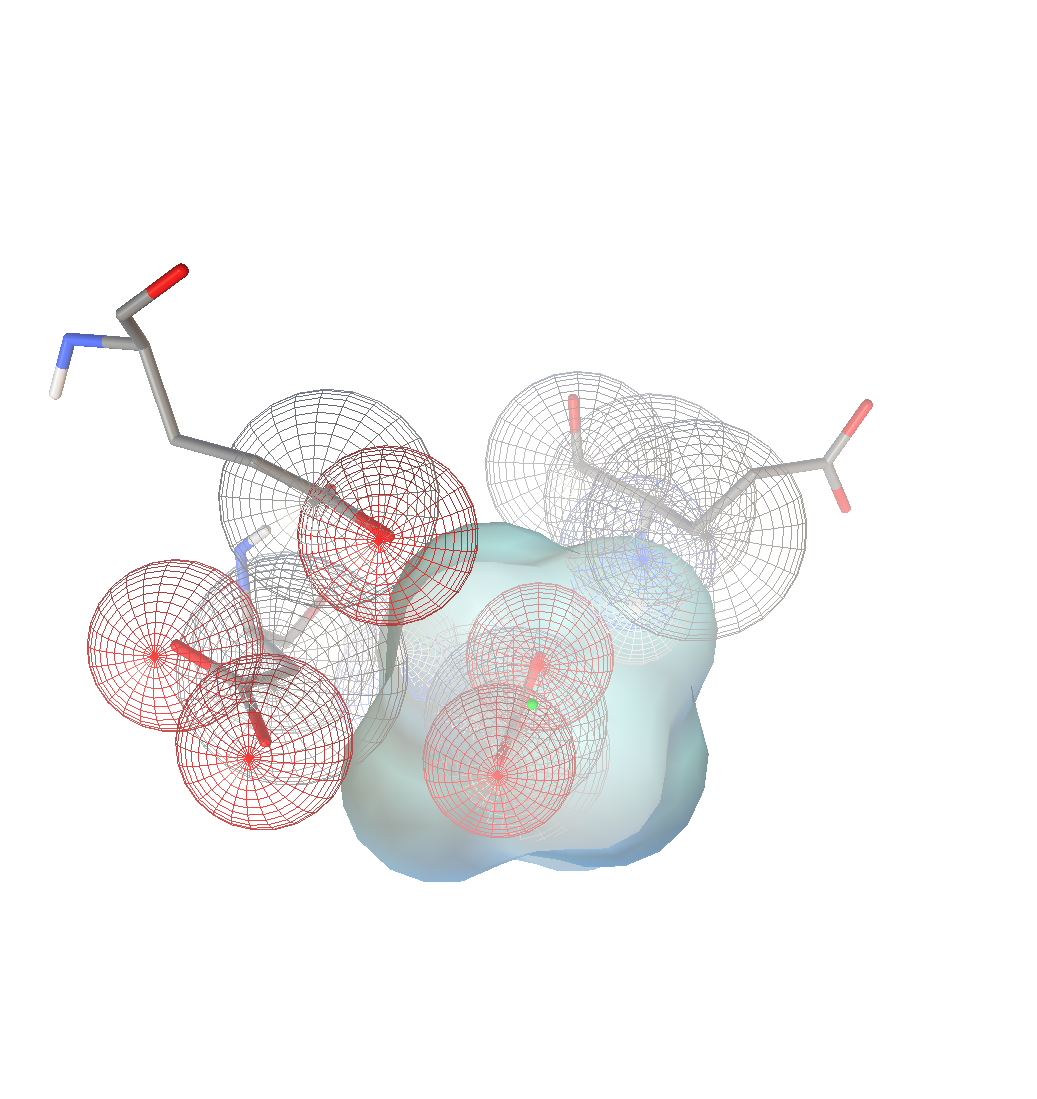

Supplement: Supplementary file 6 — Source Data 3 [file 41467_2026_72556_MOESM6_ESM.zip › source data supplementary fig 7BC/t3_TOPRIM/mg2_t4/IDNZ_mg_t3_t4_m4_2.tif]

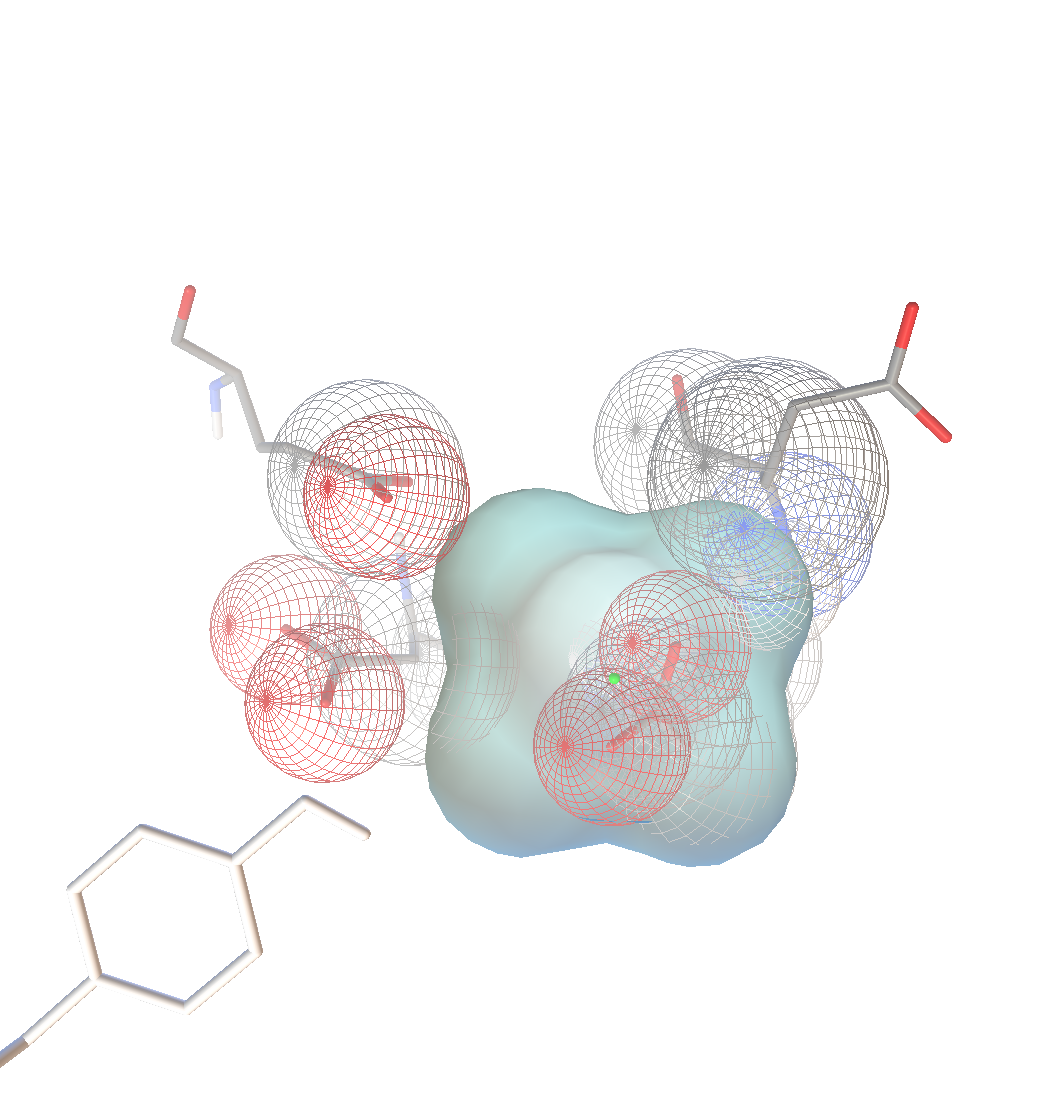

Supplement: Supplementary file 6 — Source Data 3 [file 41467_2026_72556_MOESM6_ESM.zip › source data supplementary fig 7BC/t3_TOPRIM/mg2_t4/IDNZ_mg_t3_t4_m4_3.tif]

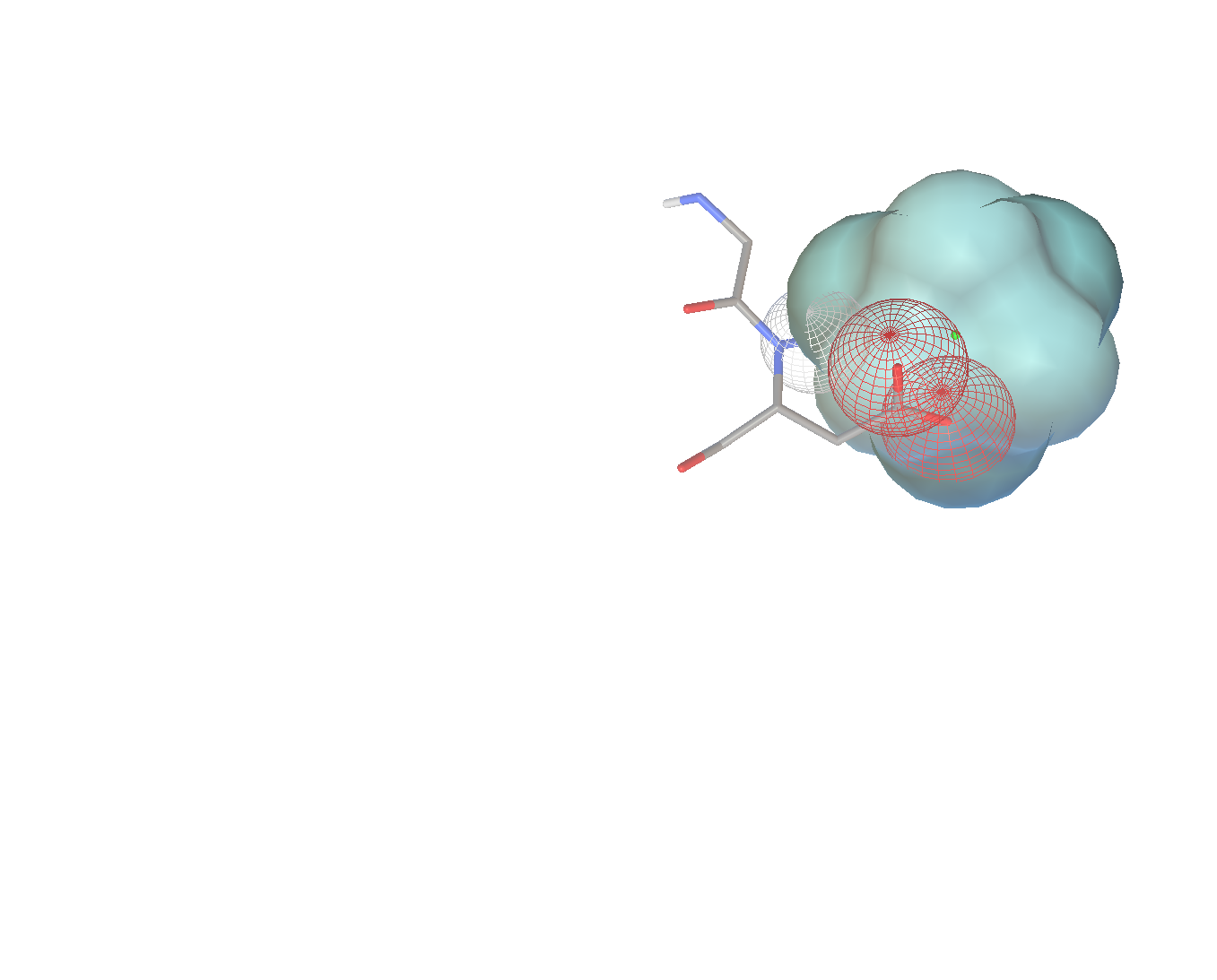

Supplement: Supplementary file 8 — Source Data 5 [file 41467_2026_72556_MOESM8_ESM.zip › source data supplementary fig 8BC/t1_DDE/top1dc_m1_1.tif]

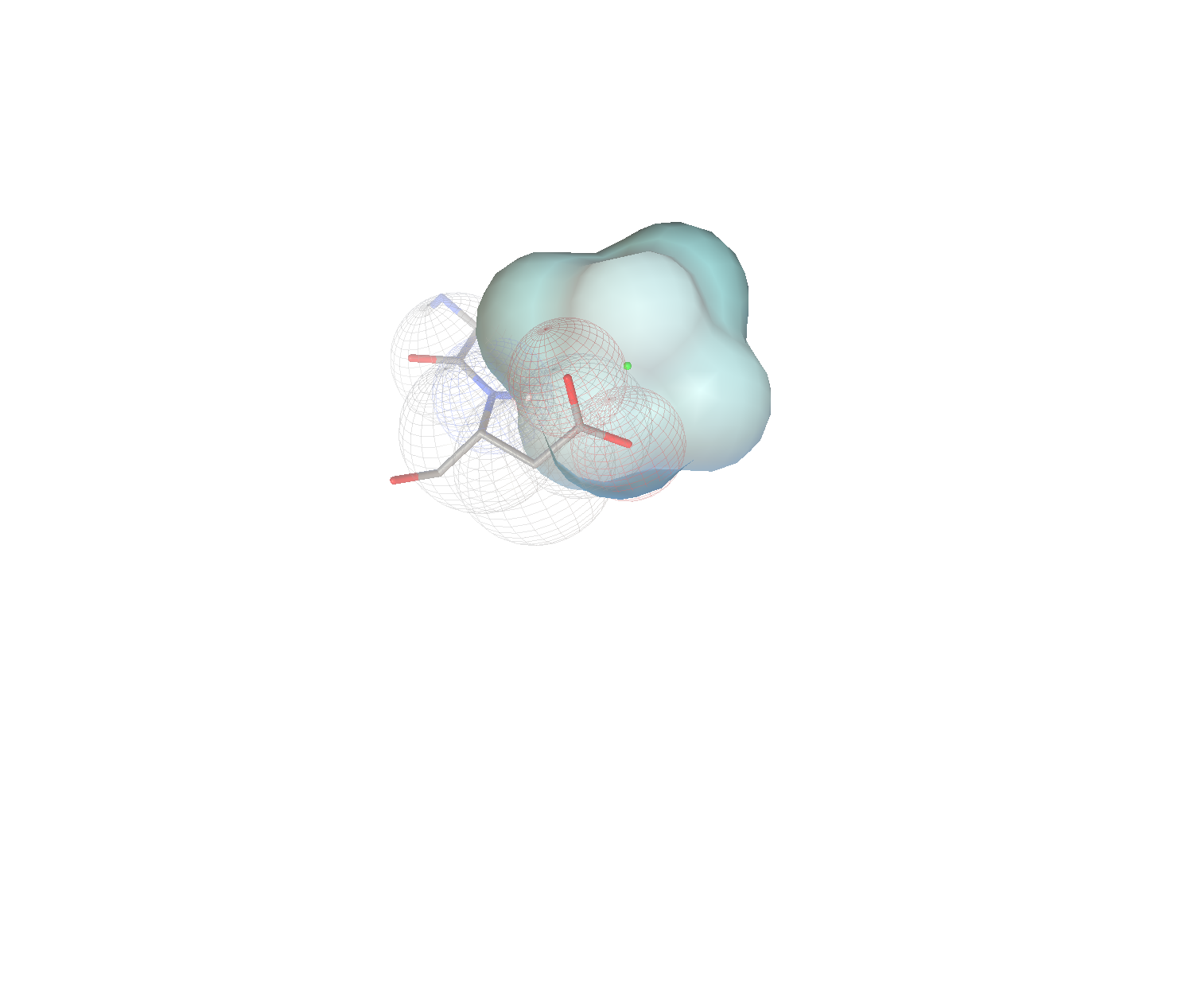

Supplement: Supplementary file 8 — Source Data 5 [file 41467_2026_72556_MOESM8_ESM.zip › source data supplementary fig 8BC/t1_DDE/top1dc_m6_1.tif]

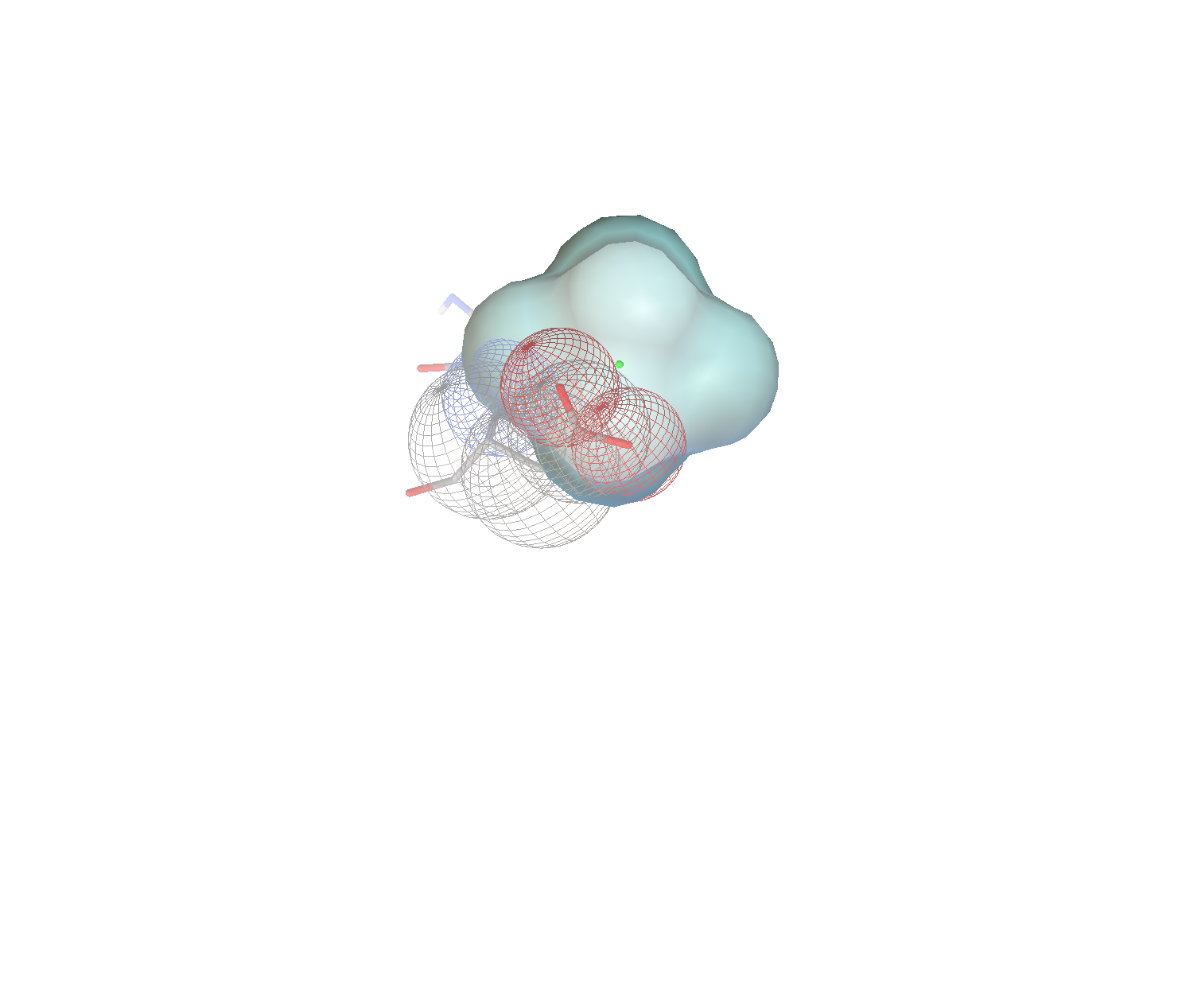

Supplement: Supplementary file 8 — Source Data 5 [file 41467_2026_72556_MOESM8_ESM.zip › source data supplementary fig 8BC/t1_DDE/top1dc_m7_1.tif]

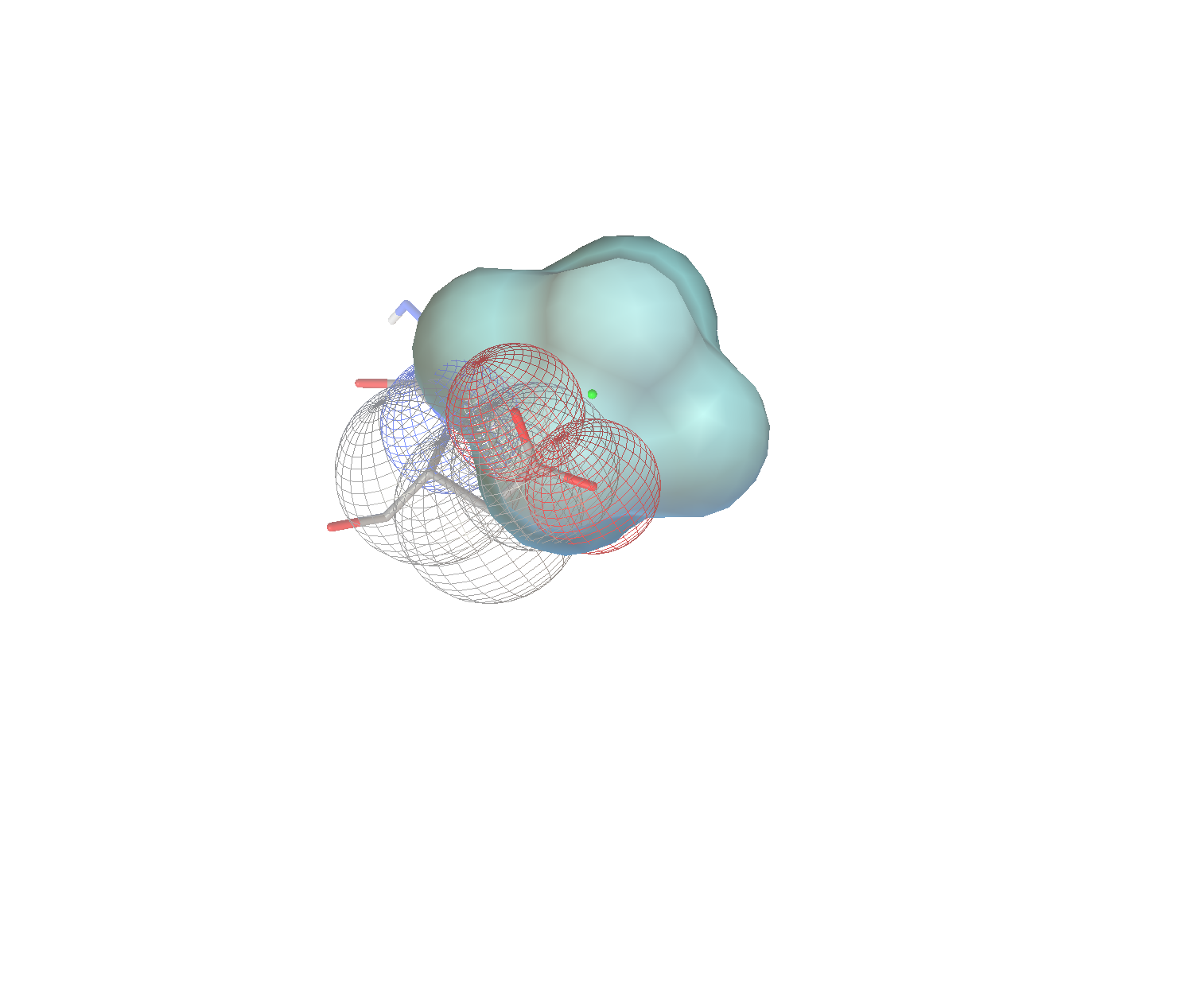

Supplement: Supplementary file 8 — Source Data 5 [file 41467_2026_72556_MOESM8_ESM.zip › source data supplementary fig 8BC/t1_DDE/top1dc_m8_1.tif]

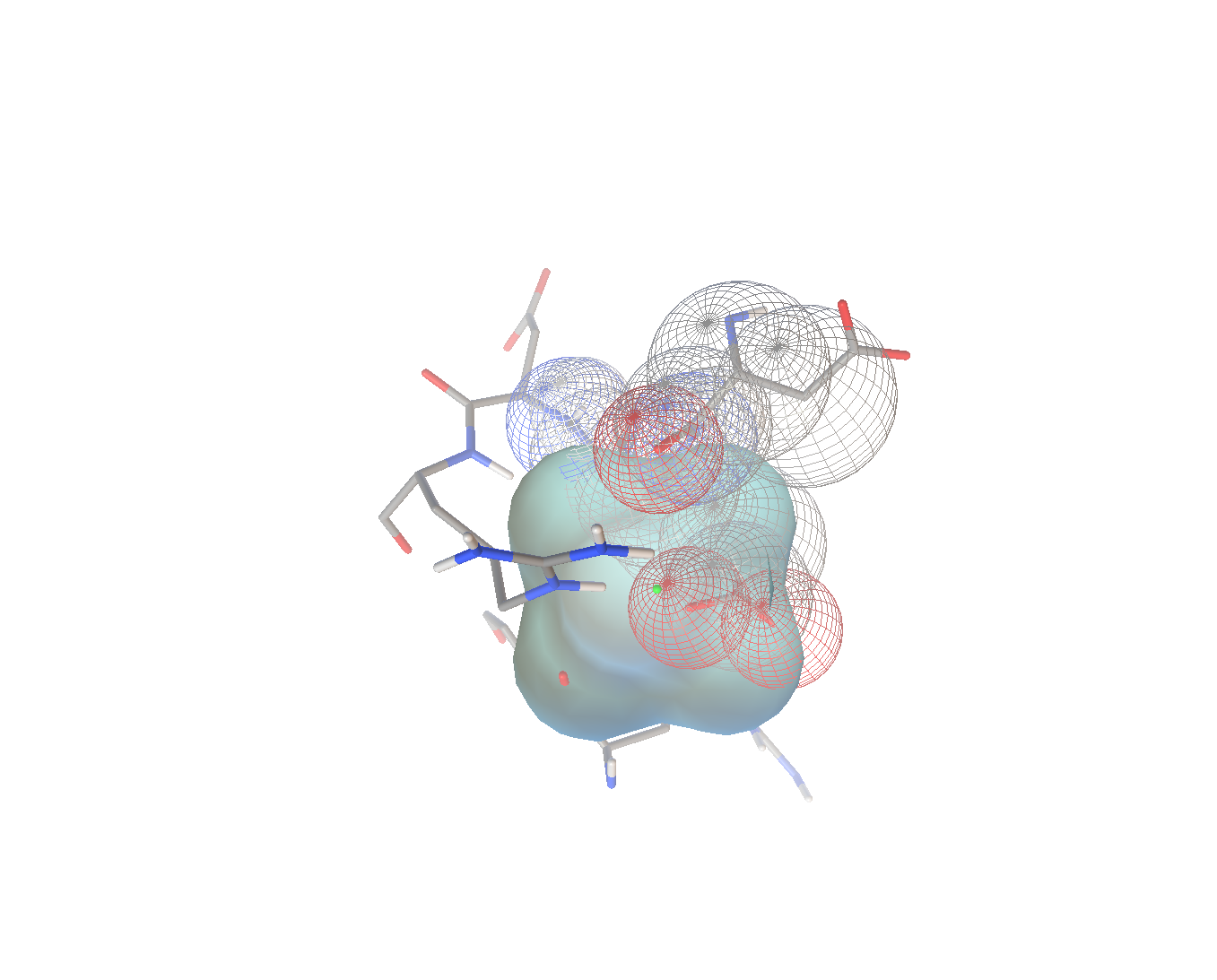

Supplement: Supplementary file 8 — Source Data 5 [file 41467_2026_72556_MOESM8_ESM.zip › source data supplementary fig 8BC/t1_DDE/top1dc_m9_1.tif]

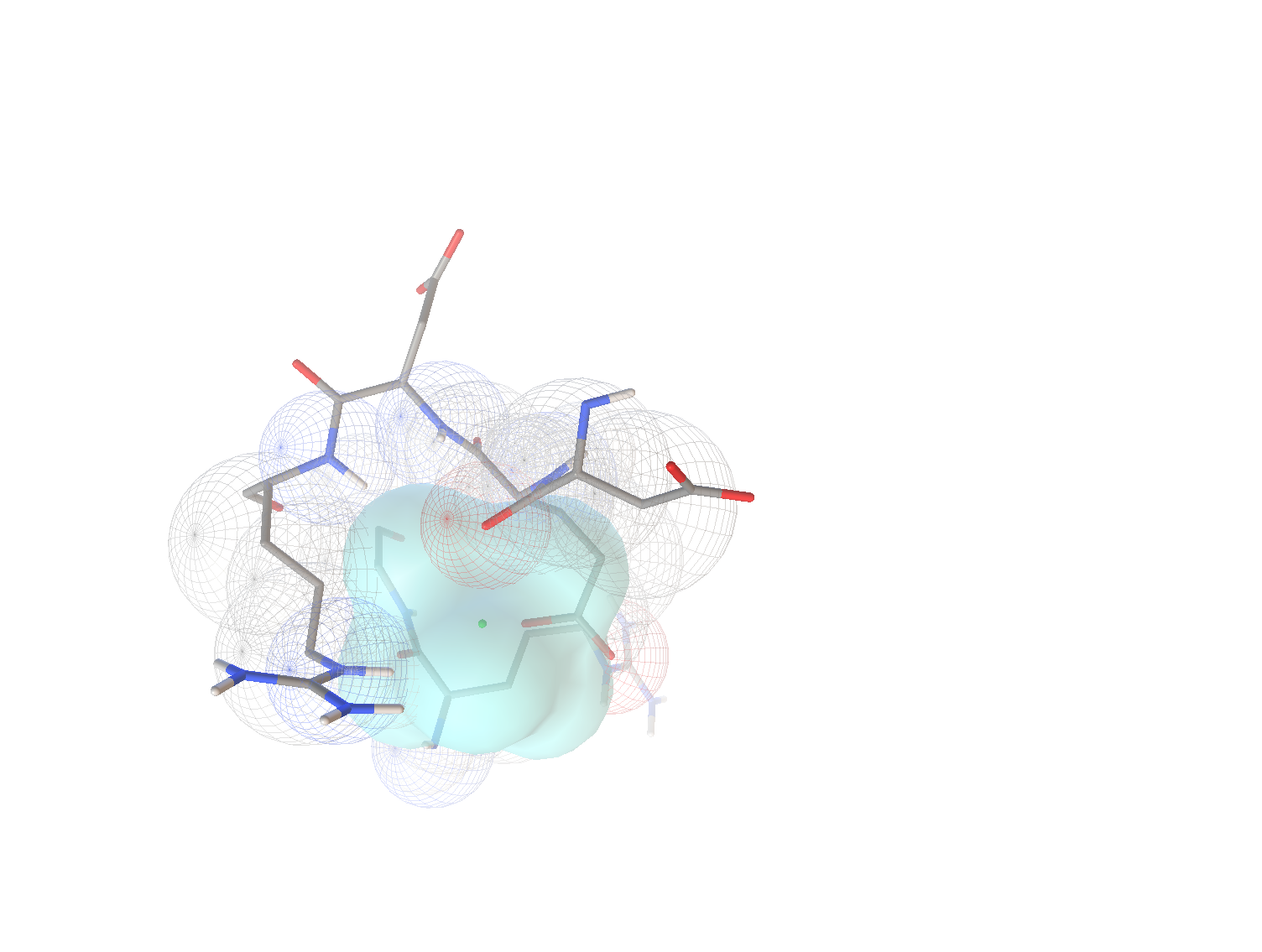

Supplement: Supplementary file 8 — Source Data 5 [file 41467_2026_72556_MOESM8_ESM.zip › source data supplementary fig 8BC/t1_DDE/top1dc_m9_2.tif]

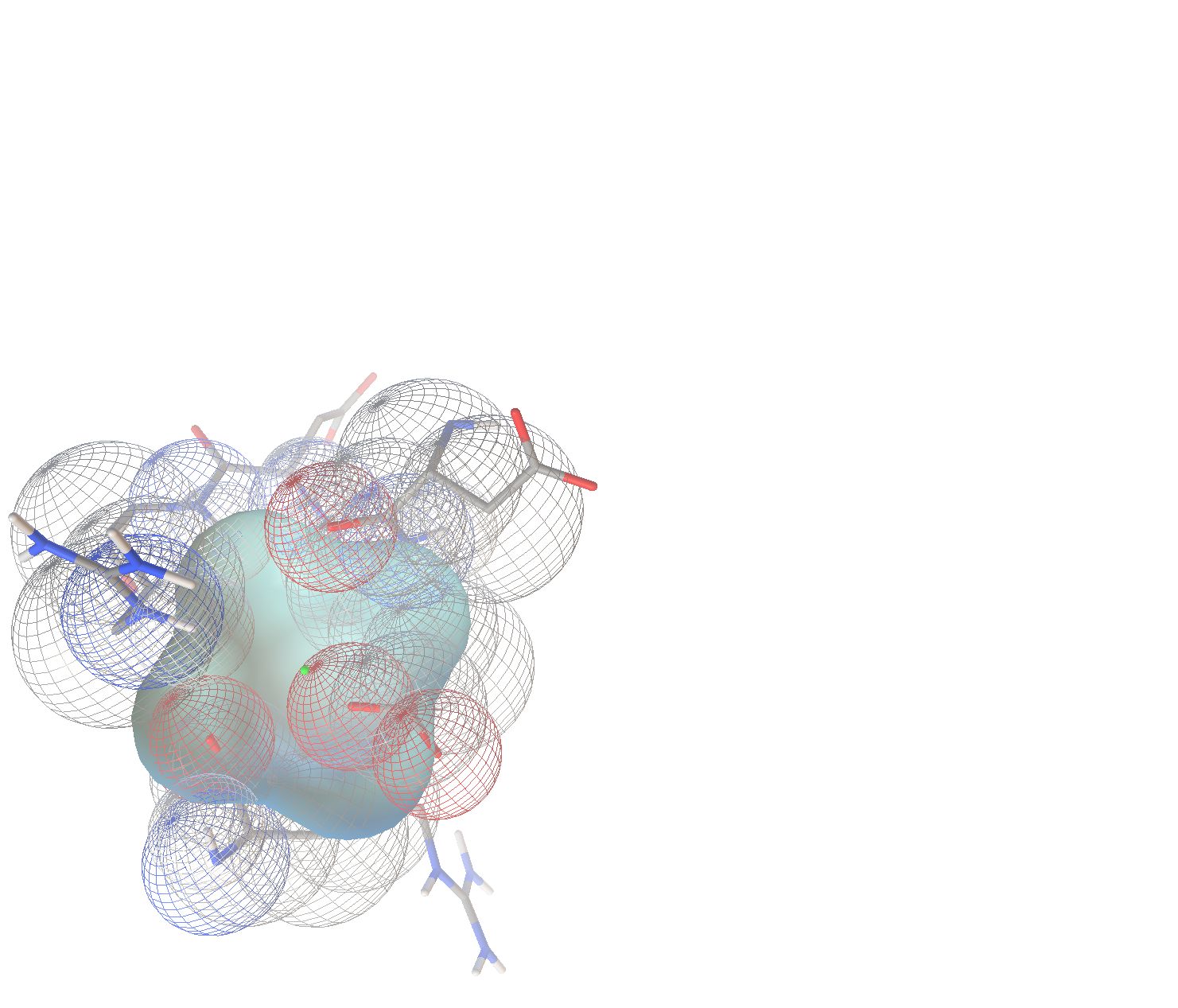

Supplement: Supplementary file 8 — Source Data 5 [file 41467_2026_72556_MOESM8_ESM.zip › source data supplementary fig 8BC/t1_DDE/top1dc_m9_3.tif]

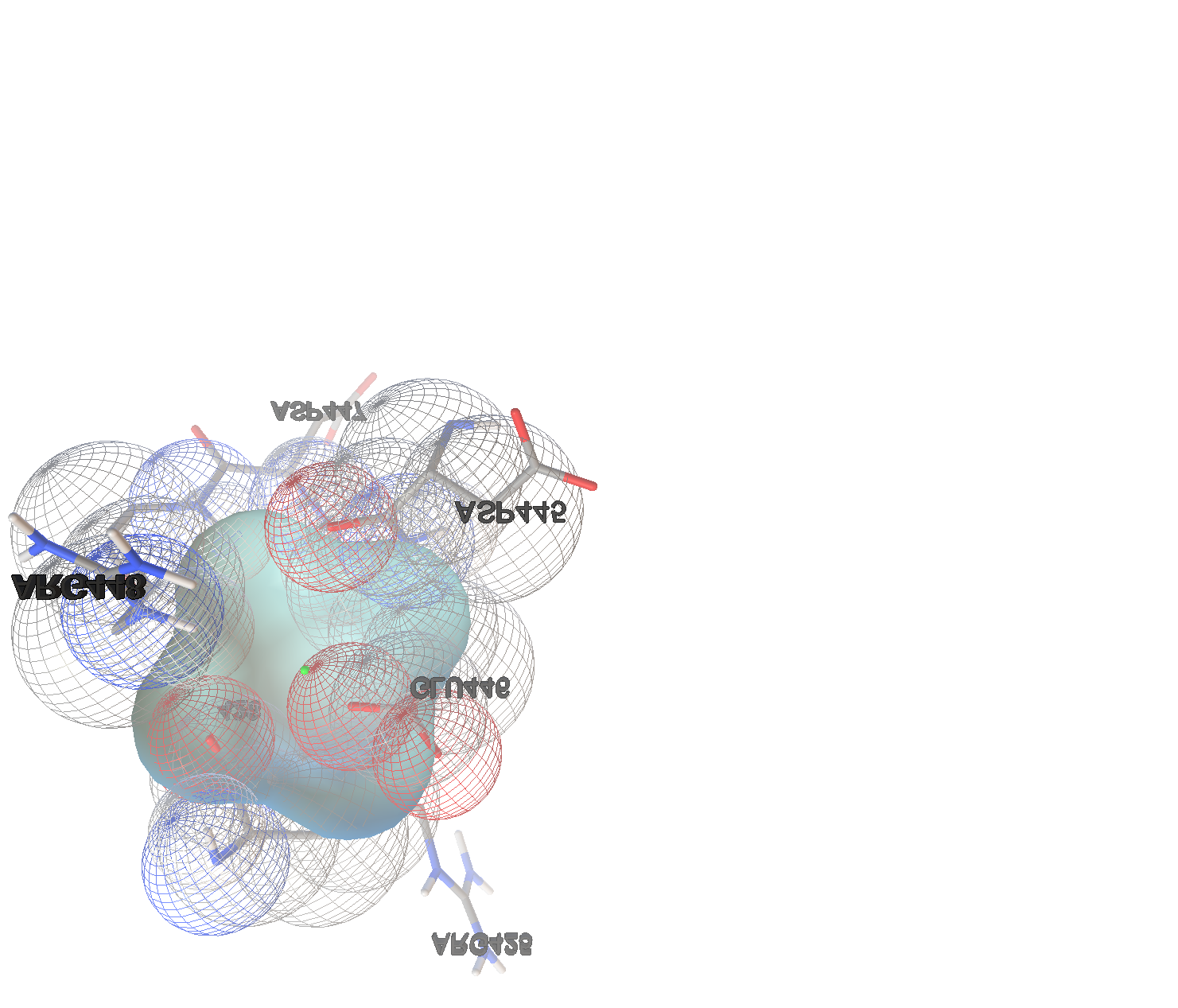

Supplement: Supplementary file 8 — Source Data 5 [file 41467_2026_72556_MOESM8_ESM.zip › source data supplementary fig 8BC/t1_DDE/top1dc_m9_3_lb.tif]

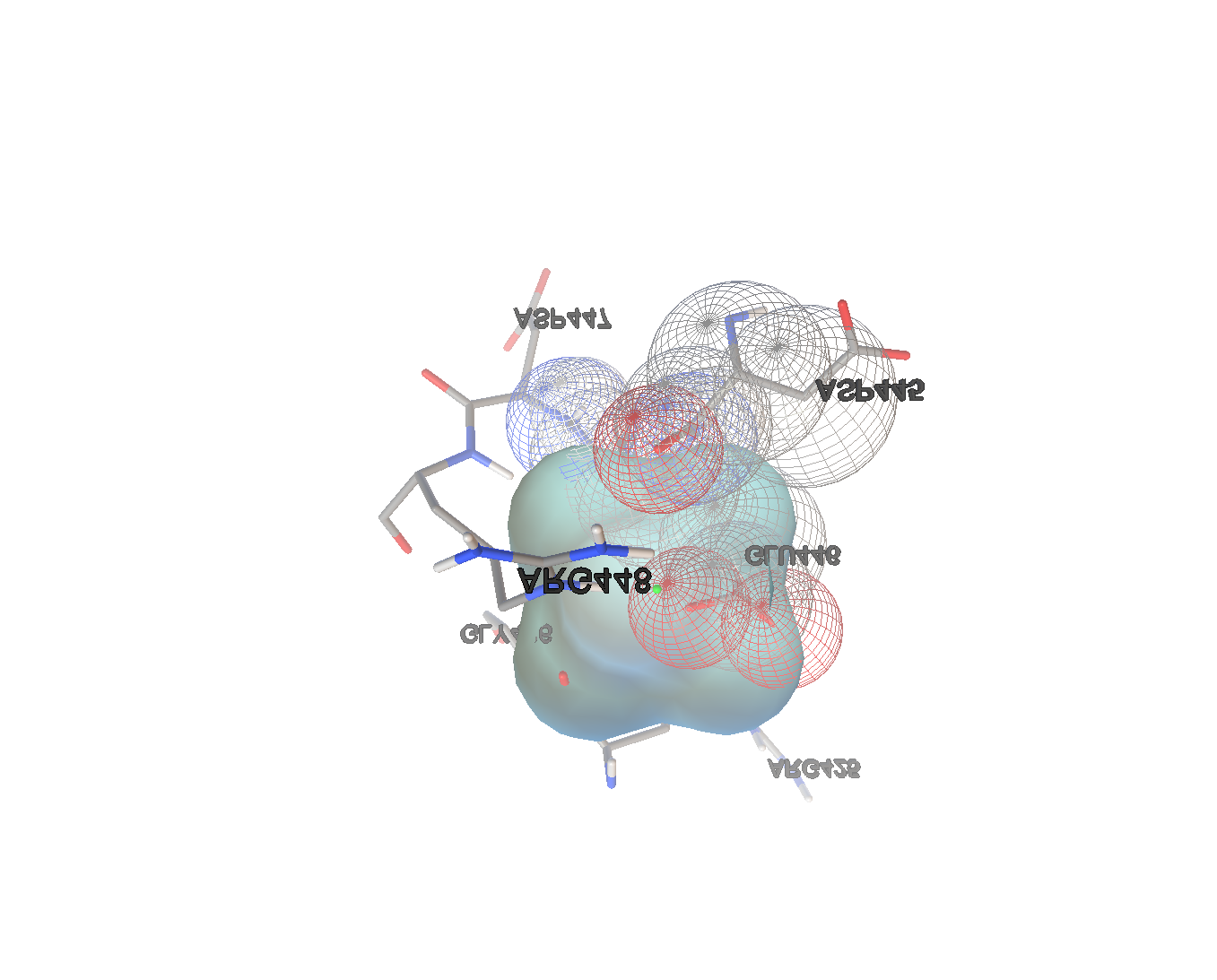

Supplement: Supplementary file 8 — Source Data 5 [file 41467_2026_72556_MOESM8_ESM.zip › source data supplementary fig 8BC/t1_DDE/top1dc_m9_lb_1.tif]

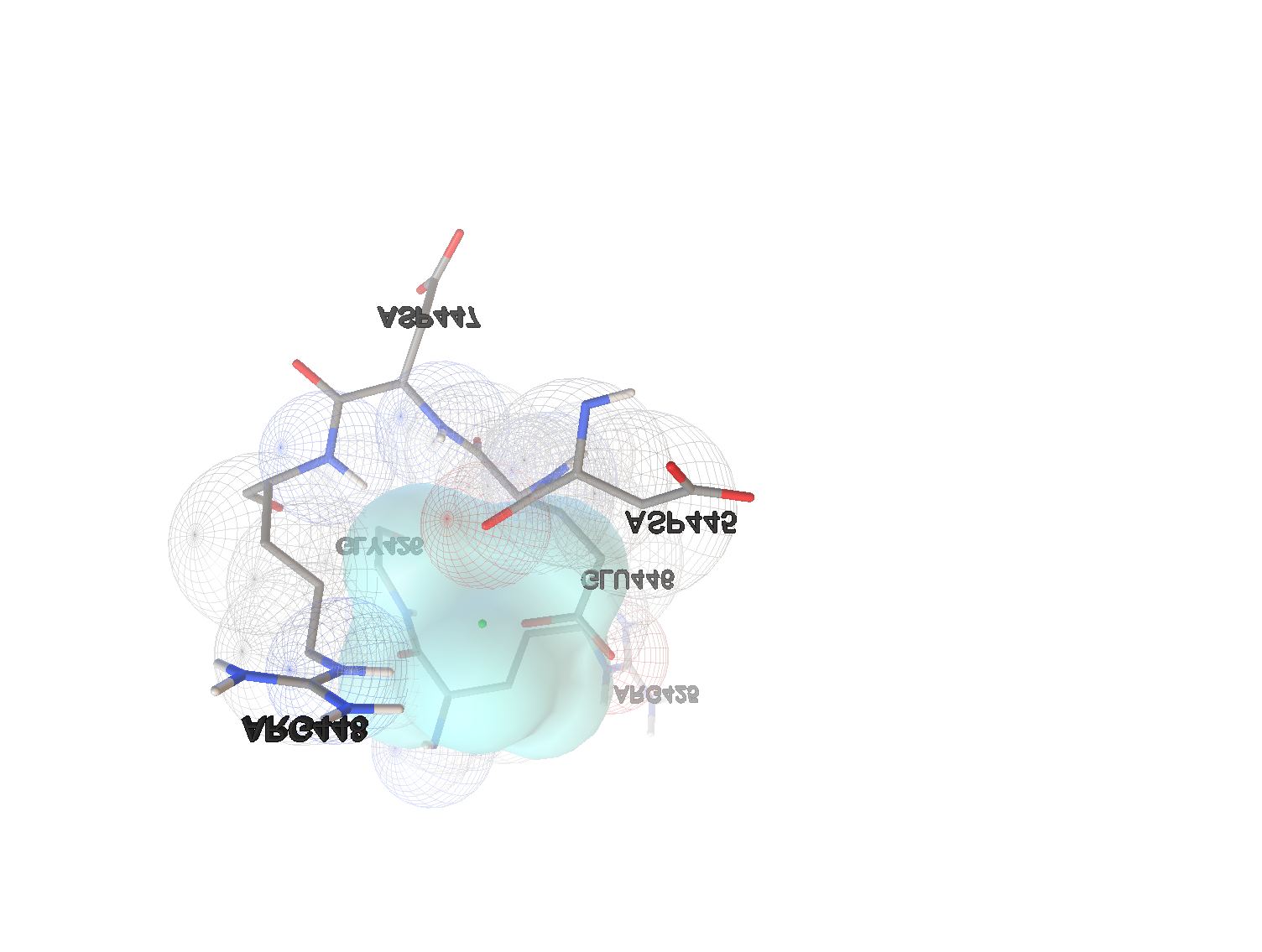

Supplement: Supplementary file 8 — Source Data 5 [file 41467_2026_72556_MOESM8_ESM.zip › source data supplementary fig 8BC/t1_DDE/top1dc_m9_lb_2.tif]

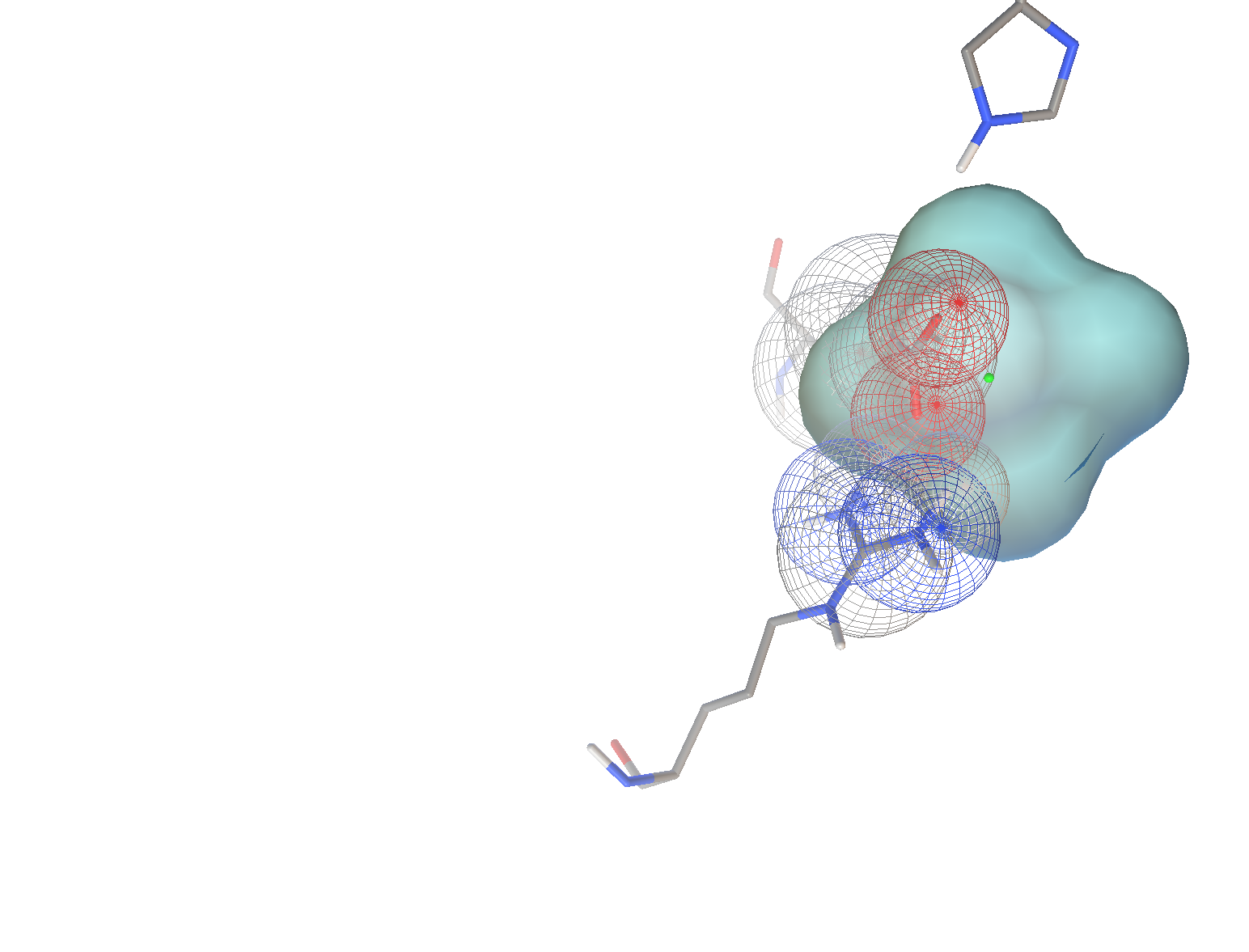

Supplement: Supplementary file 8 — Source Data 5 [file 41467_2026_72556_MOESM8_ESM.zip › source data supplementary fig 8BC/t1_TOPRIM/top1_dc_dxd_t4_mg2_m1 1.tif]

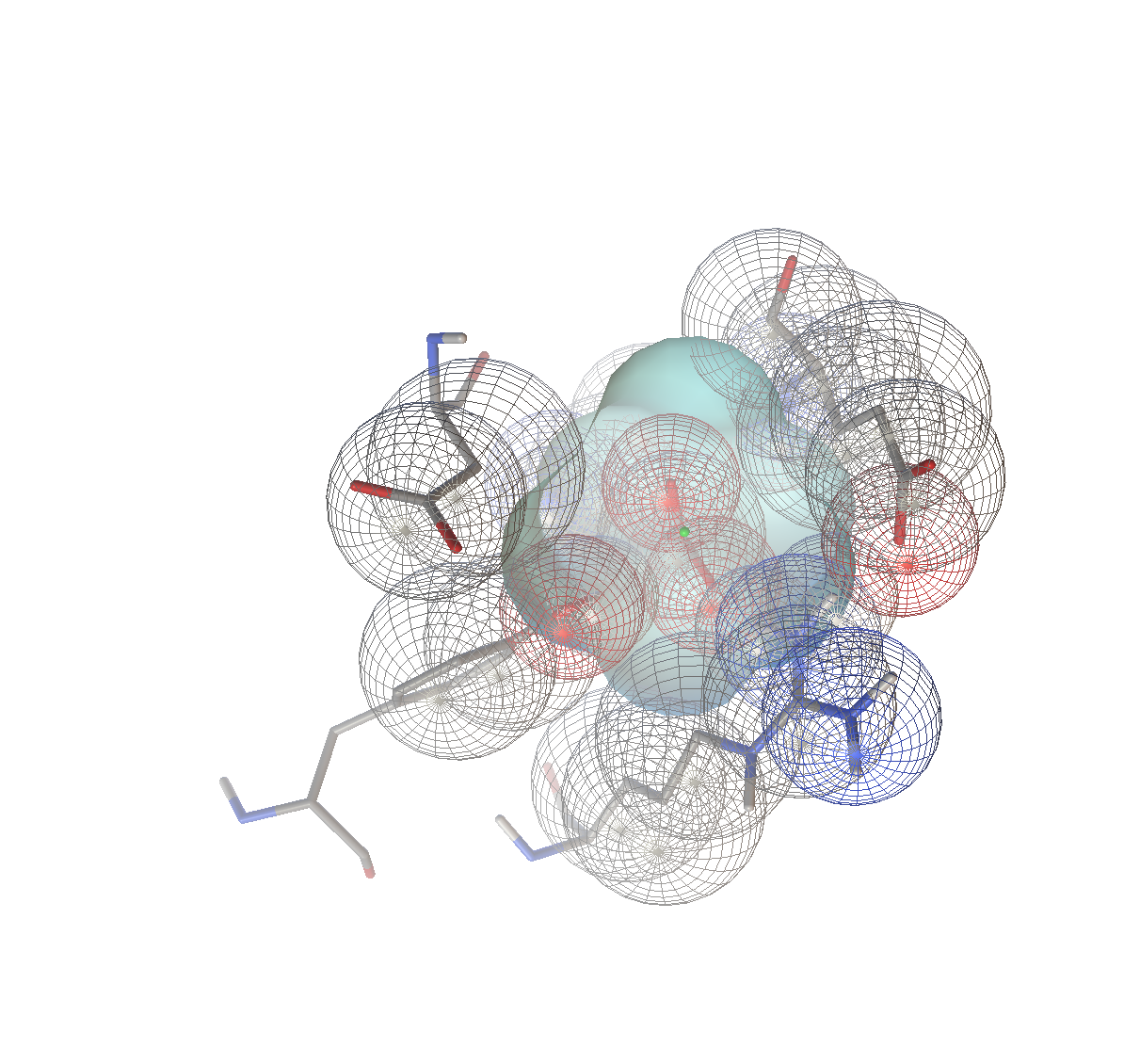

Supplement: Supplementary file 8 — Source Data 5 [file 41467_2026_72556_MOESM8_ESM.zip › source data supplementary fig 8BC/t1_TOPRIM/top1_dc_dxd_t4_mg2_m7 1.tif]

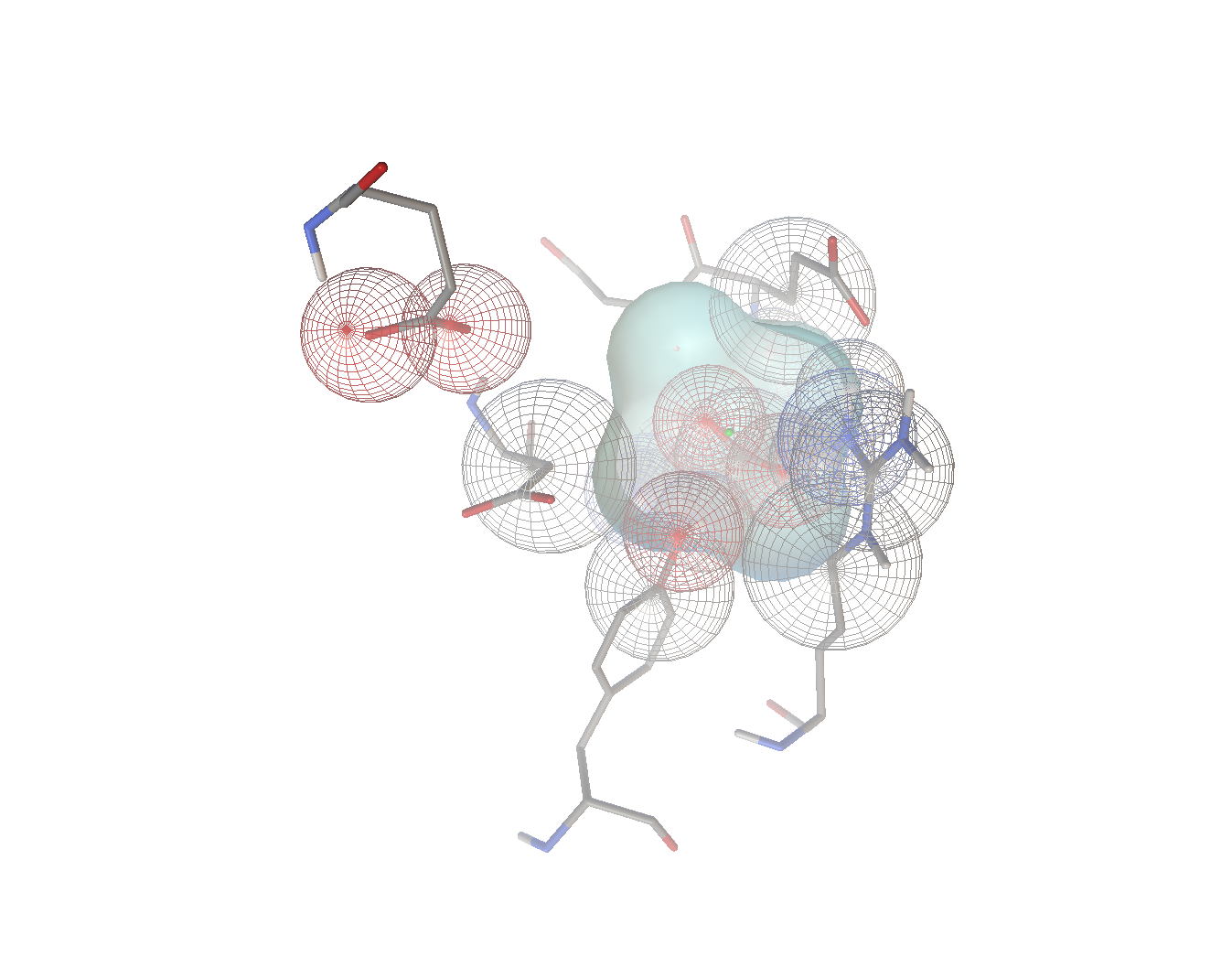

Supplement: Supplementary file 8 — Source Data 5 [file 41467_2026_72556_MOESM8_ESM.zip › source data supplementary fig 8BC/t1_TOPRIM/top1_dc_dxd_t4_mg2_m7.tif]

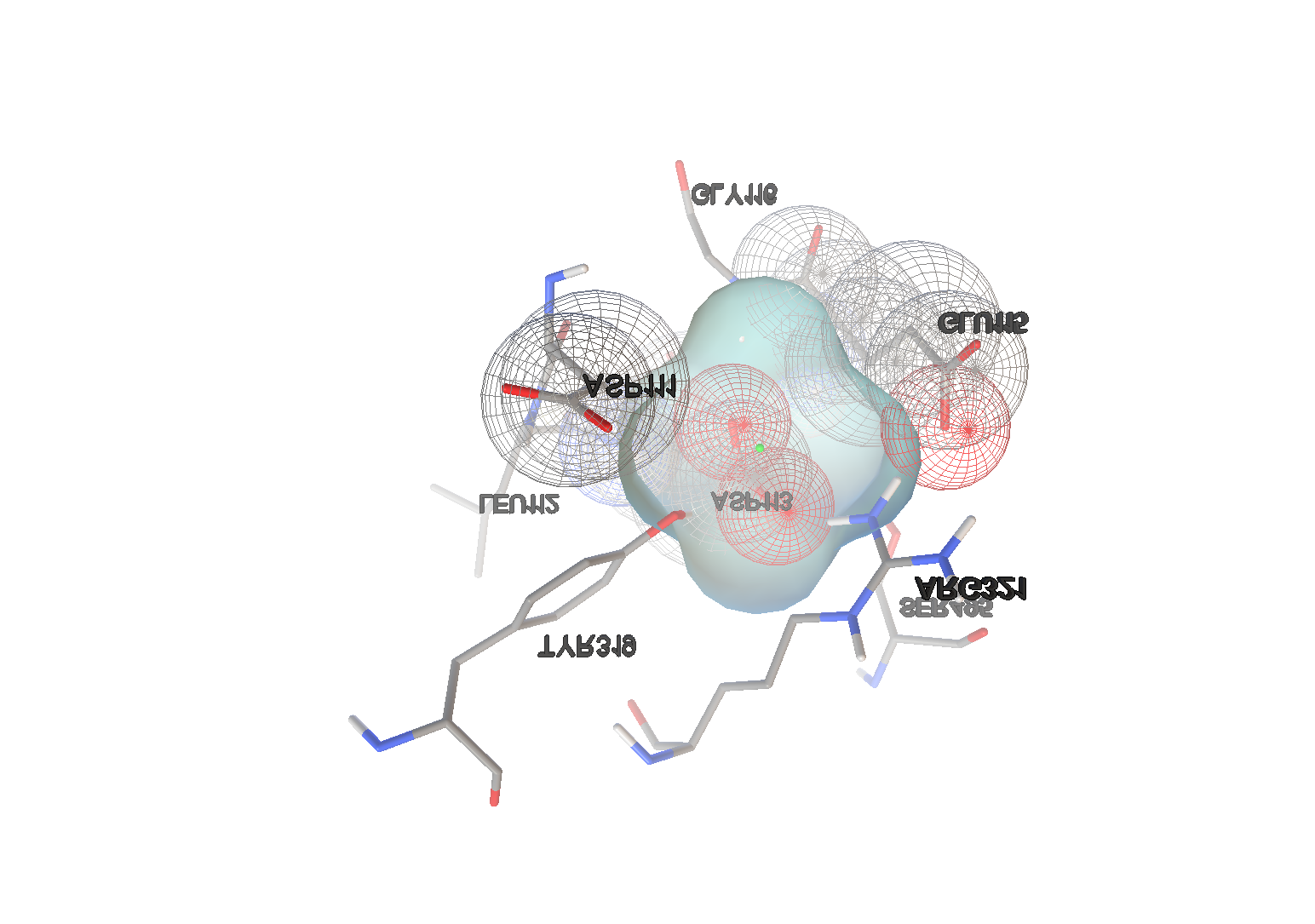

Supplement: Supplementary file 8 — Source Data 5 [file 41467_2026_72556_MOESM8_ESM.zip › source data supplementary fig 8BC/t1_TOPRIM/top1_dc_dxd_t4_mg2_m7_1.tif]

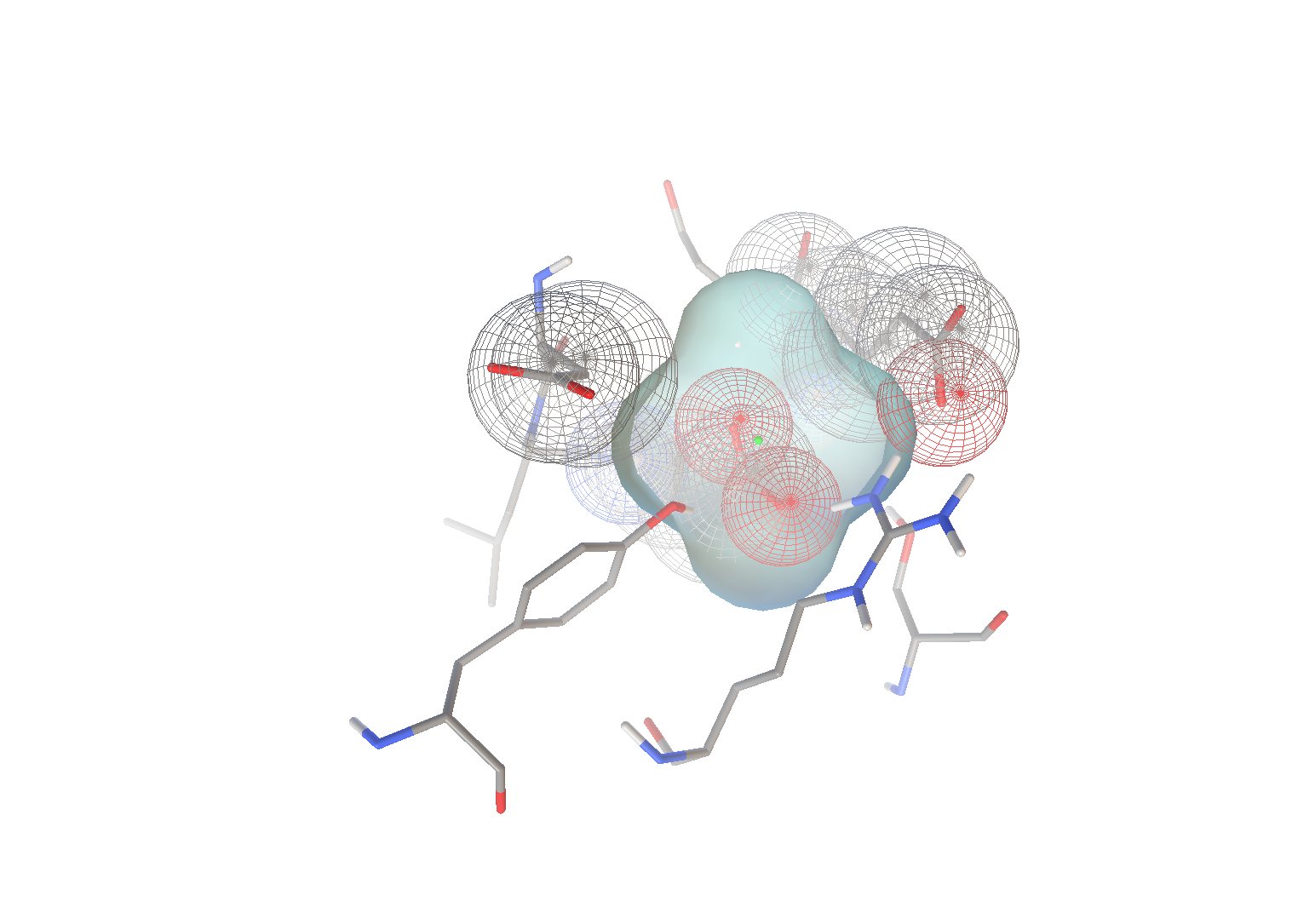

Supplement: Supplementary file 8 — Source Data 5 [file 41467_2026_72556_MOESM8_ESM.zip › source data supplementary fig 8BC/t1_TOPRIM/top1_dc_dxd_t4_mg2_m7_2.tif]

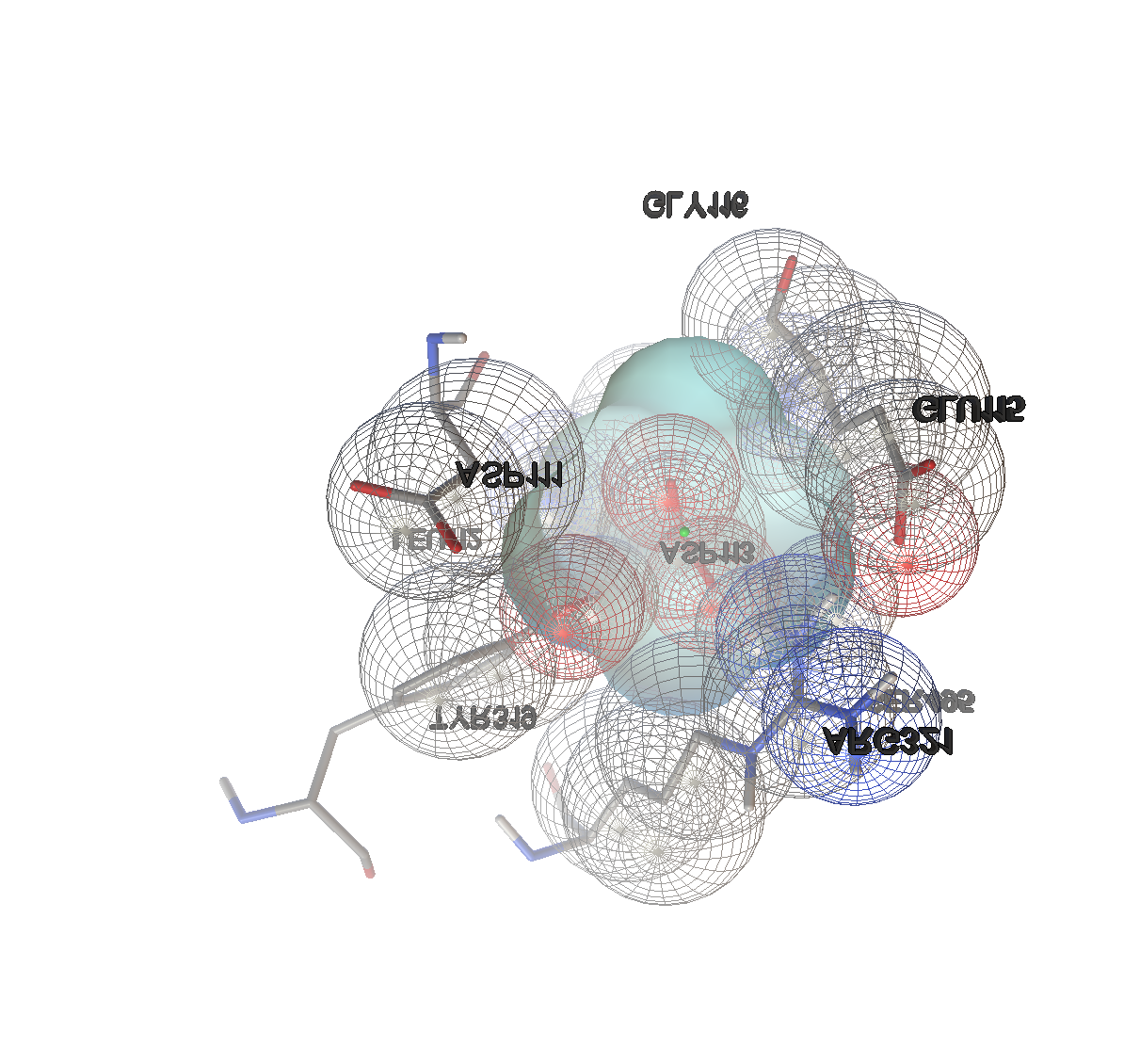

Supplement: Supplementary file 8 — Source Data 5 [file 41467_2026_72556_MOESM8_ESM.zip › source data supplementary fig 8BC/t1_TOPRIM/top1_dc_dxd_t4_mg2_m7_lb 1.tif]

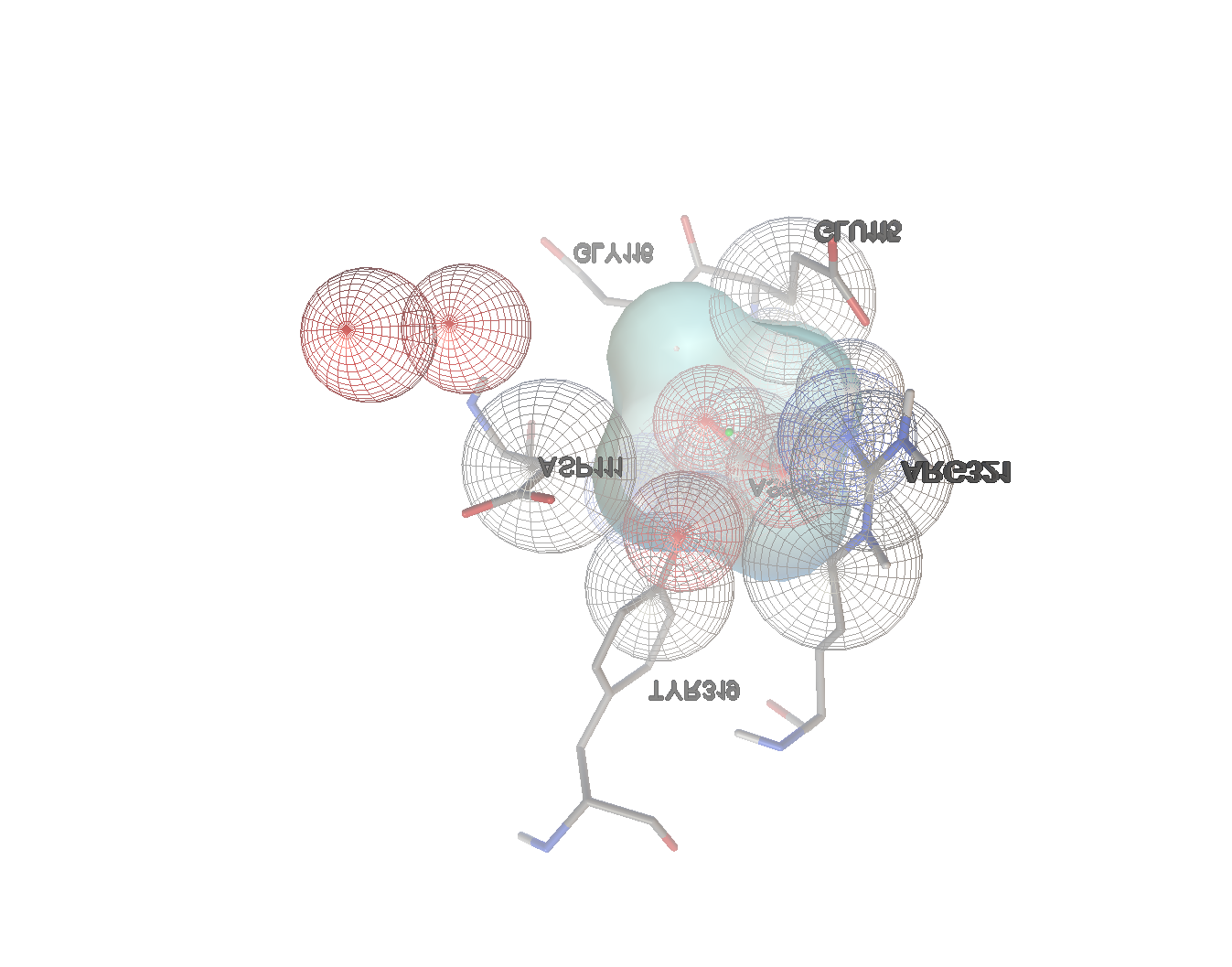

Supplement: Supplementary file 8 — Source Data 5 [file 41467_2026_72556_MOESM8_ESM.zip › source data supplementary fig 8BC/t1_TOPRIM/top1_dc_dxd_t4_mg2_m7_lb.tif]
