# Supplementary material for: Networked salt-bridges mediate magnesium-dependent conformational dynamics and functional regulation in type IA topoisomerases
Source: Nat Commun. 2026 Apr 30;17:5907. doi: 10.1038/s41467-026-72556-9 (PMC13338365; doi:10.1038/s41467-026-72556-9)
Supplement: Supplementary file 3 — Peer Review file [file 41467_2026_72556_MOESM3_ESM.pdf]

nature portfolio

Peer Review File

Networked Salt-Bridges Mediate Magnesium-Dependent Conformational Dynamics and Functional Regulation in Type IA Topoisomerases

Corresponding Author: Dr Keir Neuman

This file contains all reviewer reports in order by version, followed by all author rebuttals in order by version.

Version 0:

Reviewer comments:

Reviewer #1

(Remarks to the Author)
In this work, Seol et al. investigated how Mg2+-mediated salt bridges regulate the opening and closing kinetics of the DNA gate in type IA topoisomerases. Using single-molecule magnetic tweezers methods and bulk studies, they show that the decatenation and supercoil relaxation activities of E. coli topoisomerase 3 (ectopo3) exhibit a non-monotonic dependence on Mg^{2+} concentration, with an optimal concentration around 3 mM, decreasing below or above this concentration. To determine the rate-limiting Mg2+-dependent steps in the catalytic cycle, they applied a magnetic tweezers method that they had previously developed to study type IA topoisomerases and showed that ectopo3 requires Mg2+ for gate opening. Both the gate opening and closing rates displayed a similar non-monotonic dependence on Mg2+ concentration. They also performed steered molecular dynamics simulations that reveal two competing salt-bridge conformations, suggesting that Mg^{2+} binding to these sites catalyzes the switch between two salt-bridge configurations. This hypothesis was tested using two ectopo3 mutants bearing targeted point mutations designed to disrupt Mg 2+ binding at the predicted noncanonical sites. Based on the findings, the authors proposed a model in which transient Mg^{2+} binding catalyzes the switch between two salt-bridge configurations, thereby regulating the opening and closing of the gate.

This study provides valuable insights into the role of Mg2+ in the DNA-gate kinetics of type IA topoisomerases using a combination of different approaches. The work is well-motivated, the main text is well-written, and the studies seem to be rigorous. However, some of the single-molecule results are shown in figures without any explanation. Many of the plots are not presented in a clear and accessible format. This work is potentially suitable for publication in Nature Communications if the authors can thoroughly address the comments below.

1. The right panel of Figure 2A, the right panel of Figure 2B, and the right panels of Figure 4E show that after the force is raised to 16 pN in the presence of 3 mM Mg2+, the extension rises slowly, over a time course of 90 s. This reflects a very slow process that cannot be explained by the more rapid equilibration of the opening and closing of the gate. Note that Figure 2F shows that the equilibration rate (on rate + off rate) corresponds to an equilibration timescale of a few seconds. What gives rise to this gradual change in extension? If this extension increase is attributed to gate-opening, the authors should explain this timescale discrepancy.

2. Figure 2A shows that the hairpin was reannealed and ectopo3 was washed out of the sample chamber before the final measurement. How does ectopo3 remain bound to the DNA? Does it become associated with dsDNA? If so, this needs to be stated.

3. Figure 2C also shows an extension increase of about 400 nm. How many bound ectopo3 molecules are required to give this rise in extension? Please provide an estimate in the main text. Is this number consistent with the footprint of ectopo3 and gate opening size?

4. The right bottom panel of Figure 4E (R447G mutant) is very puzzling as the extension is below that of naked DNA, suggesting DNA compaction by this mutant. This feature needs to be explained in the main text.

5. Figure 1D shows a sawtooth pattern of the measured extension, presumably because supercoiling was repetitively

introduced into the DNA substrate after ectopo3 relaxed sufficient supercoils to remove the plectoneme. However, this is not explained and should be stated in the figure caption. How many turns were introduced at each blue line?

6. Figures 2a,b,d and Figure 4e all appear to show double-axis plots of extension and force. When the left y-axis shows extension, it is very confusing to see a force curve on the same plot. Sometimes, the extension and force curves are shown using the same color on the same plot (e.g., Figure 2b left panel). At other times, the force axis is missing (Figure 2D left panel). In most cases, it is not indicated which curve represents the extension and which represents the force. For clarity, extension and force should be shown as separate plots.

7. The data shown in Figure 2D bottom plot (3 mM Mg3+) suggest that the force biases the gate towards opening, which is expected. If so, the applied force will influence the kinetic parameters shown in Figure 2F. What was the force used in Figure 2F? Ideally, the opening and closing kinetic parameters should be obtained at zero force. Can the authors find a way to extrapolate their data to zero force?

8. The three example plots in Figure 2E show that the gate opening size depends on Mg2+ concentration: increasing from ~ 4 nm at 0.3 mM Mg2+ to ~ 8 nm at 10 mM Mg2+. What is the explanation for this increase? Do the simulations also predict this trend?

9. The number of traces for each single-molecule measurement is small (2-5 DNA molecules). What limits these data sizes? Are there technical difficulties in obtaining more traces? An explanation should be provided in the main text.

Reviewer #2

(Remarks to the Author)
I co-reviewed this manuscript with one of the reviewers who provided the listed reports. This is part of the Nature Communications initiative to facilitate training in peer review and to provide appropriate recognition for Early Career Researchers who co-review manuscripts.

Reviewer #3

(Remarks to the Author)
The manuscript describes an insightful new model of the gate opening of Topo1A that points to the key role of Mg2+ in mediating switching. The mechanistic importance of Mg2+ appears reasonable because of the high charge density on this ion.

Please can the authors clarify in the introduction which Topo1A enzymes are the focus of the paper, and why. For non-topoisomerase experts the different types of enzymes are very confusing, so it will help if it is clearer which experiments/simulations have been done on ectopo1 and which on ectopo3 and the reasons for this.

Please can the authors explain how the reaction coordinate for the steered MD was chosen, and implemented in the simulations. It might be worthwhile adding an additional figure to the supplementary information to explain this, showing the positions of the atoms where the restraints were applied, and how the results for sim1/2 were obtained. Is there Mg2+ in these simulations, and if so please can this be shown in Fig 3? Did the authors perform MD simulations with the Mg2+ located in the noncanonical binding sites identified by BioMetAll to see if they remain stable? In Figure 3, it might be helpful to indicate to the reader the position of the detail shown in 3C and 3E (e.g. with boxes or with arrows in 3A and 3D). Would the authors also consider running MD simulations of the mutated structures to show that their results are still consistent with the experimental data?

In the methods section, the authors discuss homology models of both ectopo1 and ectopo3, but in Fig 3 it appears that only ectopo3 data is presented, leaving me thinking I have missed something important. How was statistical convergence of the MD simulations assessed? The authors present more detailed data on the simulations in Extended Data Fig 8, including a calculation of the times to gate opening. The authors might consider discussing some of this interesting data in more detail either in the main paper, or expand the supplementary information to explain to the reader what conclusions can be made from these additional results. I found it confusing that the statistical analysis of the SMD appears at the beginning of the supplementary information away from Figure ED8 where it is presented. Will this be different in the final version of the paper, if it is accepted for publication?

A minor point - there is a typo on pg8 line 19 - "trough" should read "through".

Reviewer #4

(Remarks to the Author)
The manuscript of Seol and co-workers presents data to show that magnesium regulates conformational changes in type IA topoisomerases. The observation that magnesium is involved in conformational changes Is not new, it has been reported before (see below). The identification of a cluster of amino acids that are involved in conformational changes is interesting, but direct binding of magnesium to some of the amino acids is not shown, weakening the argument that they represent a secondary magnesium binding site. Molecular Dynamics (MD) simulations are used to support the role of magnesium and try to provide an atomistic explanation, but the use of a large force (50 pN) to guide the movements casts doubts on the

relevance of the calculations. Overall, the combined single molecule experiments and biochemical assays serve to confirm what had already been observed, that magnesium affects domain movements in type IA topoisomerases. The putative identification of a secondary magnesium binding site is indirect and not completely convincing. Given this, it is not clear that the work adds significantly new information to our understanding of the mechanism of type IA topoisomerases. It represents incremental and derivative work to what was known before.
Some specific comments.
1. Gunn et al. (Nat Struct Mol Biol. 2017 24(5):484-490. 2017) showed before that domain movement or gate dynamics in type IA topoisomerases is affected by magnesium. In the absence of magnesium, no domain movement was observed (see Figure 4). The authors at the very least should refer to this work and acknowledge that there was previous evidence that magnesium affects conformational changes. Whether magnesium affects directly the protein conformation or indirectly by affecting the interactions with DNA cannot be ascertained from either set of experiments.
2. The gate dynamics observed are also similar to what Gunn et al reported, with the difference that experiments were done at very different forces. Gunn et al used ~1 pN forces, while Seol et al use forces above 5 pN, which is likely to affect the time the gate remains open. The gate dynamics reported are again similar to what was already reported.
3. The identification of a second magnesium binding site is based on MD calculations and sequence analysis. Given the number of sequences available nowadays, it would be more compelling to see a larger sequence alignment with some quantitation of the presence of charged amino acids in the region. Not just a few sequences that agree with their hypothesis, but a much larger analysis that helps understand how widespread these charged regions are.
4. Binding of magnesium to the acidic residues in the putative secondary site is implied, not demonstrated. The proposed interaction is basically a charge interaction that will be affected by the presence of cations and may not necessitate direct binding. The biochemical and mutagenesis experiments support a role for the identified amino acids, but not a direct role in binding magnesium. This should be made much clearer in the manuscript. Also, it is not clear that magnesium Is the only divalent cation that would support the proposed mechanism, which could be the case if it is simply an electrostatic effect. Experiments with other divalent cations may help in this regard.
5. I am skeptical about the MD simulations as they are done at very high forces. Given the geometry selected for the simulations and the forces used, they are almost certain to show a large opening in a pre-determined direction. Some of the single molecule experiments to show gate opening are also done at very high forces (16 pN or larger), which again will bias the opening. At the very least, the implications of doing experiments and simulations at forces that clearly overcome thermal fluctuations (the likely source of domain movement in type IA topoisomerases) should be discussed.
6. The free magnesium concentration in E. coli is in the very low mM range. It is thus not surprising that the enzymes are optimal at around 3 mM. This should be mentioned.

Version 1:

Reviewer comments:

Reviewer #1

(Remarks to the Author)
All comments have been adequately addressed. The revised manuscript substantially clarifies the single-molecule methods and interpretation. This work represents a valuable contribution to understanding magnesium's role in type IA topoisomerases.

Reviewer #2

(Remarks to the Author)
I co-reviewed this manuscript with one of the reviewers who provided the listed reports. This is part of the Nature Communications initiative to facilitate training in peer review and to provide appropriate recognition for Early Career Researchers who co-review manuscripts.

Reviewer #3

(Remarks to the Author)
The authors have made substantial efforts to address the referees' comments, and those raised by me have been addressed satisfactorily.

Reviewer #4

(Remarks to the Author)
The revised manuscript of Seol et al. addresses adequately the concerns of the reviewers. The addition of data in the presence of other divalent cations does help strengthen the argument in favor of magnesium specificity.
I think that the manuscript would benefit from another round of careful editing as there are still some statements that are not clear or misleading. For example:
Page 6, line 25 and subsequent. The authors first state that “ Although simulations at such high forces are not ideal for obtaining experimentally relevant kinetics or an accurate transition path …” In the next sentence they state “The SMD results revealed that gate opening proceeds via two distinct transition pathways under …” If the simulations are not adequate to obtain a transition pathway, why mention next that a transition pathway was obtained. It seems contradictory or misleading. Clarification is needed.

Overall, the revised manuscript is stronger and the authors make a much better case for the role of magnesium in conformational changes.

Open Access This Peer Review File is licensed under a Creative Commons Attribution 4.0 International License, which permits use, sharing, adaptation, distribution and reproduction in any medium or format, as long as you give appropriate credit to the original author(s) and the source, provide a link to the Creative Commons license, and indicate if changes were made.
In cases where reviewers are anonymous, credit should be given to 'Anonymous Referee' and the source.
The images or other third party material in this Peer Review File are included in the article's Creative Commons license, unless indicated otherwise in a credit line to the material. If material is not included in the article's Creative Commons license and your intended use is not permitted by statutory regulation or exceeds the permitted use, you will need to obtain permission directly from the copyright holder.
To view a copy of this license, visit https://creativecommons.org/licenses/by/4.0/

We thank the referees for their careful reading of the manuscript and the time they took to provide constructive comments, suggestions, and criticisms. We have addressed the reviewers' comments and revised the manuscript accordingly. The revised manuscript is significantly improved thanks to the insights and suggestions from the reviewers.

To address the reviewers' suggestions and concerns we have included new data and analyses. The main additions are as follows:

1. We tested high monovalent salt conditions (300 mM) to demonstrate that the magnesium-dependent gate conformational change is not due to non-specific electrostatic effects (Extended Data Fig. 3C–F).

2. We measured the effects of Ca^{2+} and Mn^{2+} on gate dynamics, gate opening, and catalytic activity of topoisomerase enzymes, showing that the effects of magnesium on gate conformational dynamics are unique and markedly different from those of Ca^{2+} or Mn^{2+} (Extended Data Fig. 6; new figure).

3. We performed AutoDock computational docking of a hydrated magnesium ion to the putative binding site to confirm the BioMetall results (Extended Data Fig. 7B and 8B).

4. We include magnesium-insensitive gate dynamics data for 2E2Q to highlight the involvement of the two acidic residues in magnesium binding, which is critical for magnesium-dependent gate dynamics (Extended Data Fig. 12C).

5. We expanded the sequence alignment of ecTopo1A and included several additional AlphaFold predicted structures of bacterial topo3 along with BioMetall predictions of divalent metal binding.

We address the reviewer's comments point-by-point below. The revised texts in the rebuttal and in the manuscript are highlighted in yellow.

Reviewer #1 (Remarks to the Author):

In this work, Seol et al. investigated how Mg2+-mediated salt bridges regulate the opening and closing kinetics of the DNA gate in type IA topoisomerases. Using single-molecule magnetic tweezers methods and bulk studies, they show that the decatenation and supercoil relaxation activities of E. coli topoisomerase 3 (ectopo3) exhibit a non-monotonic dependence on Mg^{2+} concentration, with an optimal concentration around 3 mM, decreasing below or above this concentration. To determine the rate-limiting Mg2+-dependent steps in the catalytic cycle, they applied a magnetic tweezers method that they had previously developed to study type IA topoisomerases and showed that ectopo3 requires Mg2+ for gate opening. Both the gate opening and closing rates displayed a similar non-monotonic dependence on Mg2+ concentration. They also performed steered molecular dynamics simulations that reveal two competing salt-bridge conformations, suggesting that Mg^{2+} binding to these sites catalyzes the switch between two salt-bridge configurations. This hypothesis was tested using two ectopo3 mutants bearing targeted

point mutations designed to disrupt Mg 2+ binding at the predicted noncanonical sites. Based on the findings, the authors proposed a model in which transient Mg^{2+} binding catalyzes the switch between two salt-bridge configurations, thereby regulating the opening and closing of the gate.

This study provides valuable insights into the role of Mg2+ in the DNA-gate kinetics of type IA topoisomerases using a combination of different approaches. The work is well-motivated, the main text is well-written, and the studies seem to be rigorous. However, some of the single-molecule results are shown in figures without any explanation. Many of the plots are not presented in a clear and accessible format. This work is potentially suitable for publication in Nature Communications if the authors can thoroughly address the comments below.

1. The right panel of Figure 2A, the right panel of Figure 2B, and the right panels of Figure 4E show that after the force is raised to 16 pN in the presence of 3 mM Mg2+, the extension rises slowly, over a time course of 90 s. This reflects a very slow process that cannot be explained by the more rapid equilibration of the opening and closing of the gate. Note that Figure 2F shows that the equilibration rate (on rate + off rate) corresponds to an equilibration timescale of a few seconds. What gives rise to this gradual change in extension? If this extension increase is attributed to gate-opening, the authors should explain this timescale discrepancy.

We thank the referee for pointing out the apparent inconsistency in the gate kinetics between the two experimental measurements (fig. 2A and fig.2F). As the referee correctly points out, the experiment shown in Fig 2F captured the gate kinetics of one prebound enzyme on a single-stranded gapped substrate at equilibrium. On the other hand, the experiment in Fig 2A corresponds the monitoring of multiple (up to ~57) individual enzyme binding, DNA cleavage, and gate opening events occurring over ~90 s. The resulting gradual increase in extension of the extended ssDNA is expected given the heterogenous and unsynchronized nature of individual enzyme binding, which limits the observed increase in extension associated with subsequent cleavage, and gate-opening processes.

Additionally, and related to question 2 below, we realized that the cartoon describing the enzymes bound to the single-stranded DNA after buffer exchange in Fig. 2A might create further confusion as it could give the impression that all enzymes were bound to the single-stranded DNA immediately after the DNA hairpin was opened, rather than gradually binding.

In the revised manuscript, we have modified the cartoon in Fig. 2A to indicate that enzymes bind to the newly exposed single-stranded DNA upon hairpin opening.

2. Figure 2A shows that the hairpin was reannealed and ectopo3 was washed out of the sample chamber before the final measurement. How does ectopo3 remain bound to the DNA? Does it become associated with dsDNA? If so, this needs to be stated.

We thank the referee for raising this point. As the referee suggested, the topo IA enzymes likely remain associated with duplex DNA in the refolded hairpin. However, it is also possible that they weakly and reversibly bind to the surface or to the beads. As our intention was to exchange the

buffer from 1mM EDTA to 3 mM $MgCl_2$ without flushing out all the enzymes from the sample chamber, we applied a slow flow rate (~2 mm/s: corresponding to a maximum force of ~ 0.04 pN on 1 nm sized particles at the viscosity of 10^-3 in MKS unit), which would remove unattached enzymes, but not weakly bound enzymes. We stress that the important result is that gate opening as revealed by increase in extension of the ssDNA only occurs after the buffer exchange with magnesium. The fact that we observe gate opening with reduced enzyme concentration after the buffer exchange, strengthens the findings as no extension increase was observed for presumably higher enzyme concentrations under magnesium depletion conditions.

To improve clarity, we have changed the text "wash out unbound enzymes" to "buffer exchange" to more accurately reflect the experiments. Additionally, we have added the following text in the manuscript as suggested.

Change made to the main text:

Original text:

"Enzyme binding and catalytic competence were confirmed by robust gate opening upon exchange to 3 mM Mg^{2+} protein free buffer (Fig. 2A)."

Revised text (page 4_line31):

" DNA binding and catalytic competence of TopIA enzymes were confirmed by the robust gate opening of the remaining enzymes after buffer-exchange with 3 mM Mg^{2+} protein free buffer "

Addition to the main text (page5_line1):

"We note that the buffer exchange reduced the free enzyme concentration in the sample chamber. The fact that robust ssDNA extension increase was observed only after buffer exchange despite the associated reduction in enzyme concentration, strengthens the finding that Mg^{2+} is required for gate opening as no gate opening was observed at higher concentrations of enzyme in the absence of Mg^{2+}.

3. Figure 2C also shows an extension increase of about 400 nm. How many bound ectopo3 molecules are required to give this rise in extension? Please provide an estimate in the main text. Is this number consistent with the footprint of ectopo3 and gate opening size?

We thank the referee for raising these important questions. Based on the gate-opening size (~7 nm) estimated from the previous study with a similar DNA hairpin substrate and force[1], the observed ~400 nm increase in extension corresponds to ~57 bound enzymes. Using the previously determined footprint of ectopo3 (14 nt)[2] and the 1158 nt of ssDNA, the maximum number of ectopo3 that can bind would be 83. Thus, ~57 bound enzymes is possible. We have included this information in the main text.

Change made to the main text:

Original text:

“which exposes 1158 nucleotides of single-stranded DNA for TopIA to bind”

Revised Text (page4_line26):

“which exposes 1158 nucleotides of single-stranded DNA for multiple TopIA to bind (up to ~83 based on the reported 14 nt footprint of ectopo3 on ssDNA)”

Addition to the main text (page4_line33):

“The ~400 nm extension increase over 90 s reflects DNA binding and gate opening of ~57 enzymes based on the previously estimated 7 nm gate opening measured under similar conditions (Fig. 2A-C). ”

Figure caption 2B:

“the extension increased gradually (green line), indicating unsynchronized DNA binding, cleavage, and gate-opening of multiple enzymes”

4. The right bottom panel of Figure 4E (R447G mutant) is very puzzling as the extension is below that of naked DNA, suggesting DNA compaction by this mutant. This feature needs to be explained in the main text.

We thank the referee for pointing out this subtle but confusing point. We apologize for not presenting a clearer explanation and have clarified this point in the revised manuscript.

The dashed line corresponds to the DNA extension when the hairpin is fully opened without enzyme as described in fig. 2A (top left). The measured extension below the dashed line in the presence of enzyme indicates that some R447G enzymes prevent the DNA hairpin from fully opening, presumably by binding to both strands of the hairpin duplex, while others bound to the exposed single-stranded DNA followed by gate opening result in the observed increase in extension. The abrupt and relatively large increases in extension predominantly observed in the presence of the R447G mutant, likely represent release of one strand in the hairpin, lending support to this interpretation of the reduced extension of the unzipped hairpin DNA in comparison with the naked unzipped hairpin.

Change made to the main text

Original text:

“Furthermore, R447G resisted gate opening even in the presence of Mg^{2+} as (Fig. 4E-F)”

Revised text (page8_line6):

"Furthermore, R447G not only resisted gate opening, but also prevented the DNA hairpin from fully opening, indicated by the negative average extension change (dEx), likely by binding to both strands and stabilizing the DNA hairpin (Fig. 4E-F)…".

Addition to the fig. 4E caption:

"The reduced extension of the hairpin relative to the fully opened hairpin (dashed line) in the presence of R447G with 3 mM Mg^{2+} likely results from enzyme binding to the duplex DNA preventing full hairpin opening (negative dEx)."

5. Figure 1D shows a sawtooth pattern of the measured extension, presumably because supercoiling was repetitively introduced into the DNA substrate after ectopo3 relaxed sufficient supercoils to remove the plectoneme. However, this is not explained and should be stated in the figure caption. How many turns were introduced at each blue line?

We thank the referee for pointing out the incomplete description of the supercoiling data presented in figure 1D. Although we provided the information in the methods, we agree with the reviewer and have included the relevant information in the revised figure 1D and accompanying caption.

Addition made to the fig.1D caption:

"Once DNA supercoils were relaxed by the enzyme, the indicated number of magnet rotations (15 or 20) (right axis and label) were applied to re-supercoil the DNA."

6. Figures 2a,b,d and Figure 4e all appear to show double-axis plots of extension and force. When the left y-axis shows extension, it is very confusing to see a force curve on the same plot. Sometimes, the extension and force curves are shown using the same color on the same plot (e.g., Figure 2b left panel). At other times, the force axis is missing (Figure 2D left panel). In most cases, it is not indicated which curve represents the extension and which represents the force. For clarity, extension and force should be shown as separate plots.

We thank the referee for pointing out the lack of clarity of the plots. Due to limited figure space, we overlaid the force as a guideline but omitted information deemed unessential from the figure. We understand that it could be confusing and difficult to interpret the graphs with two similar colored lines representing force and the data trace at 1 mM EDTA. We have revised the figures (Figs. 2 and 4) to address this issue by changing the color of the force trace to light orange and moved it behind the data traces to minimize obstructing the extension data. Additionally, we have added the force axis and force information to the graphs for clarity.

Addition made to the fig. 2F caption:

"at an equilibrium force ~5 pN"

7. The data shown in Figure 2D bottom plot (3 mM Mg3+) suggest that the force biases the gate towards opening, which is expected. If so, the applied force will influence the kinetic parameters

shown in Figure 2F. What was the force used in Figure 2F? Ideally, the opening and closing kinetic parameters should be obtained at zero force. Can the authors find a way to extrapolate their data to zero force?

We thank the referee for commenting on the zero force measurement. First, the dynamics shown in Fig.2F were obtained from analysis of the single molecule measurements at 5 pN (Example traces shown in Fig. 2E). We agree with the referee that it would be ideal to obtain kinetics at zero force. However, it is not feasible to estimate the zero force rates by extrapolation as it would require kinetic rate measurements at an additional 3 to 4 forces around the equilibrium force for all four magnesium conditions, which is beyond the scope of the current work. As the main point of the current study was to investigate magnesium-dependent gate conformational dynamics, we chose an intermediate force of 5 pN at which the opening and closing rates were comparable and slow enough to be reliably measured. These measurements are sufficient to demonstrate the effect of magnesium on the opening and closing rates, and test the proposed model.

8. The three example plots in Figure 2E show that the gate opening size depends on Mg2+ concentration: increasing from ~ 4 nm at 0.3 mM Mg2+ to ~ 8 nm at 10 mM Mg2+. What is the explanation for this increase? Do the simulations also predict this trend?

We thank the referee for pointing this subtle issue related to apparent increases in the gate size with increasing magnesium concentration. Although there does appear to be a slight increase in the gate-opening size at higher Mg concentrations in the example traces (Fig. 2E), the average gate size differences among the four magnesium concentrations are not statistically different based on the Tukey test. This possible effect, though it is interesting as it could imply that higher magnesium concentrations may allow an additional gate conformational change leading to a larger gate opening similar to that at very high force, is beyond the scope of the current work.

9. The number of traces for each single-molecule measurement is small (2-5 DNA molecules). What limits these data sizes? Are there technical difficulties in obtaining more traces? An explanation should be provided in the main text.

We thank the reviewer for this question. Since many events were obtained from each tether, we considered that 2-5 independent measurements would be sufficient to confirm reproducibility. For clarity, we have changed “number of DNA molecules” to “number of independent measurements”, as each DNA tether was measured in a different sample chamber on a different day. Additionally, we have collected new data so that all measurements were included a minimum of 3 independent measurements.

In response to the reviewer’s question about the technical difficulties in obtaining the single-molecule traces, the short answer is that the gate kinetics measurements were challenging and very low throughput (<10% success rate for each tether). To ensure that observed extension changes reflected enzyme activity we performed rigorous testing of each tether and hairpin

construct in the absence of enzyme to verify they behaved as expected and there were no spurious fluctuations. The testing and subsequent measurements required DNA tethers to survive under relatively high forces for hours. Many DNA tethers failed during initial testing or before meaningful data could be collected. As a result the overall throughput was relatively low and data collection was challenging, but the quality and reproducibility of the data was improved.

Reviewer #2 (Remarks to the Author):

I co-reviewed this manuscript with one of the reviewers who provided the listed reports. This is part of the Nature Communications initiative to facilitate training in peer review and to provide appropriate recognition for Early Career Researchers who co-review manuscripts.

Reviewer #3 (Remarks to the Author):

The manuscript describes an insightful new model of the gate opening of Topo1A that points to the key role of Mg2+ in mediating switching. The mechanistic importance of Mg2+ appears reasonable because of the high charge density on this ion.

Please can the authors clarify in the introduction which Topo1A enzymes are the focus of the paper, and why. For non-topoisomerase experts the different types of enzymes are very confusing, so it will help if it is clearer which experiments/simulations have been done on ectopo1 and which on ectopo3 and the reasons for this.

We thank the reviewer for the suggestion and apologize for the confusion. Our current work, was mainly focused on ectopo3 as it is a minimal type IA topoisomerase that lacks the extra domains found in ectopo1 and does not require other interacting protein partners for its stability and function as shown for eukaryotic topo3. We have revised the introduction to clarify this choice of ectopo3 as the main focus of the work. We included confirmatory measurements and simulations of ectopo1 (mainly in the supplementary information) to confirm magnesium-dependent gate dynamics and identify a similar, though non-identical, magnesium binding site and salt-bridge network in ectopo1. However, we have not investigated ectopo1 via simulation and mutagenesis experiments to the same extent as ectopo3.

To streamline and clarify the text, we have removed ectopo1 information from the Steered molecular dynamics (SMD) methods section and added it to extended data fig.8A caption. Additionally, we have added a sentence to address the referee suggestion as below.

Addition made to the main text (page3_line10):

" Whereas corroborating measurements and simulations were performed with *ectopo1* to extend the results obtained with *ectopo3* and for the sake of comparison with previous studies [12,16], our

current work is focused on *ectopo3* as it is a minimal topo IA consisting of a toroidal core without additional extended domains or required a complex formation with other proteins. "

Please can the authors explain how the reaction coordinate for the steered MD was chosen, and implemented in the simulations. It might be worthwhile adding an additional figure to the supplementary information to explain this, showing the positions of the atoms where the restraints were applied, and how the results for sim1/2 were obtained.

We thank the referee for the question and suggestion. We modified the Extended data fig. 9 (previously extended data fig. 8) and caption to address the referee's comment. The reaction coordinate and constrained residues for the simulations mimic the experimental reaction coordinate that is set by the tension on the DNA bound to the enzyme in domain I on one side of the gate and the tension on the DNA covalently attached to the catalytic tyrosine on the other side of the gate. Specifically for the simulations, the reaction coordinate is aligned along the backbone of the DNA bound to domain I. The constraint residues were selected based on their known interactions with bound DNA based on the crystal structures[3,4].

The results for all simulations including sim1 and 2 shown in Fig. 3 and extended data fig. 9 were obtained from the analysis of the trajectories of all atoms in DCD format that were recorded every 10 ps during the simulations. Taking the reviewer's suggestion, we have expanded the text in the method to provide the more detailed description of how the results shown in fig. 3 and extended data fig. 9 in the revised manuscript.

<u>Change made to the main text</u>

Original text:

"Salt-bridge detection in the simulation trajectories was performed using VMD Salt Bridges plugin [5]. The CA atom trajectories of the catalytic tyrosine were obtained with the Python script based on ProDy [6], the generation of plots and statistical analysis were done in Igor Pro 9 (Wavemetrics)."

Revised text (page14_line18):

"Salt-bridge detection in the simulation trajectories was performed using the VMD Salt Bridges plugin [5], which identified all salt-bridge forming sidechain pairs and provided the center-of-mass distances (COM) between these pairs at each time step of the trajectories. The Cα atom trajectories of the catalytic tyrosine for generating gate conformational changes in x and y were obtained with a Python script based on ProDy [6], which calculated the COM of all atoms within 5Å of the catalytic tyrosine Cα atom. The x and y position probability density histograms, plot generation, and statistical analyses were performed in Igor Pro 9 (Wavemetrics).

Addition to the extended fig. 9A caption:

"The SMD atom (Cα of catalytic tyrosine 328) is depicted as a red sphere, while the constraint atoms (Cα of residues 103, 105, and 165) are shown as magenta spheres. The vector between the

two phosphates at positions 3 and 6 of the bound single-stranded DNA backbone (violet) is indicated by a red dashed line. This vector was used as a reference to rotate the structure, aligning the bound DNA along the x-axis (inset)."

Is there Mg2+ in these simulations, and if so please can this be shown in Fig 3? Did the authors perform MD simulations with the Mg2+ located in the noncanonical binding sites identified by BioMetAll to see if they remain stable? In Figure 3, it might be helpful to indicate to the reader the position of the detail shown in 3C and 3E (e.g. with boxes or with arrows in 3A and 3D). Would the authors also consider running MD simulations of the mutated structures to show that their results are still consistent with the experimental data?

We apologize for any confusion regarding magnesium (Mg^{2+}) ions in the MD simulations. Although Mg^{2+} ions were included at a concentration corresponding to 3 mM, the SMD simulations were not designed to emulate the physical characteristics of magnesium ions or to capture specific interactions between acidic residues and Mg^{2+}. Our current simulations focused solely on residue interactions that influence gate conformations, not on the interactions of magnesium with specific binding residues. Simulations including interactions between Mg^{2+} and the acidic residues are not feasible and beyond our expertise. To perform MD simulations with magnesium bound at a predicted site, a protein structure with Mg^{2+} or another divalent metal ion captured in the binding site is required as an initial configuration. This structure should ideally show which acidic residues form direct bonds with Mg^{2+} and which interact indirectly via water molecules. Unfortunately, such structures are not currently available for topo IA enzymes. Furthermore, simulating the process of hydrated Mg^{2+} interacting with acidic residues, alongside the concurrent conformational changes of the gate domain, would be computationally prohibitive. Our longer-term goal is to obtain magnesium-bound structures of topo IA enzymes, which would lay the groundwork for MD simulations including magnesium bound at the acidic triad we identified in this work. However, such simulations and the necessary structural data are beyond the scope of the current work.

To address current limitations and further support the BioMetall predictions, we used AutoDock to evaluate magnesium binding at both the newly identified site and the canonical site. Because magnesium ions are hydrated in aqueous solution, we docked a magnesium ion together with its first hydration shell rather than the bare ion. The results demonstrating a relatively low affinity (~ 10μM K_d) are presented in the revised manuscript. (Extended Data Figs. 7 and 8).

Additionally, we have added text to clarify that magnesium binding to the predicted residues was not detected during the simulations, to prevent any further confusion.

<u>Addition made to the main text (page7_line3):</u>

"We note that despite the presence of Mg^{2+} in the simulations, Mg^{2+} binding to the predicted residues was not observed as the SMD was not configured to model Mg^{2+}–protein-residue interactions."

The reviewer makes a great suggestion of indicating the region corresponding to the enlarged views shown in Figure 3C and E. We have taken this advice and have included a box on the structure in Figure 3A highlighting the enlarged region in Figure 3C and E.

Addition made to the main text in the fig. 3A caption:

"The boxed area indicates the region of interest enlarged in C and E."

We appreciate the suggestion of running simulations of the mutants to confirm the measured effects of the mutations on gate dynamics. The results of the simulations of the mutant enzymes, presented in extended data Fig. 9, are entirely consistent with the model predictions, and the experimental findings.

In the methods section, the authors discuss homology models of both ectopo1 and ectopo3, but in Fig 3 it appears that only ectopo3 data is presented, leaving me thinking I have missed something important. How was statistical convergence of the MD simulations assessed? The authors present more detailed data on the simulations in Extended Data Fig 8, including a calculation of the times to gate opening. The authors might consider discussing some of this interesting data in more detail either in the main paper, or expand the supplementary information to explain to the reader what conclusions can be made from these additional results. I found it confusing that the statistical analysis of the SMD appears at the beginning of the supplementary information away from Figure ED8 where it is presented. Will this be different in the final version of the paper, if it is accepted for publication?

We thank the reviewer for pointing out the potentially confusing discussion of homology modeling of both ectopo3 and ectopo1 while only results for ectopo3 were presented in Figure 3. We have corrected this potential confusing presentation of the simulation process by limiting the methods section to describing the process of simulating only ectopo3. We have moved the description of the complementary simulations of ectopo1 to the caption for extended data Fig. 9 that describes the simulation results for ectopo1.

The reviewer raises an interesting point regarding the statistical convergence of the molecular dynamics simulations. Since the simulations were steered molecular dynamics simulations, they are inherently out of equilibrium and thus we do not expect to achieve statistical convergence, but obtained the time averaged trajectories. To address related questions of the accuracy and reproducibility of the molecular dynamics simulations, we performed 8 independent simulations for each condition. Despite the inherent stochasticity and out of equilibrium nature of the simulation trajectories, we observed only two distinct gate-opening pathways. Importantly, the two gate opening pathways were associated with specific salt-bridge interactions as discussed in Fig 3A. Moreover, the gate opening free energy landscapes were dramatically altered in simulations of specific point-mutations that were predicted to alter the salt-bridge interactions. These results and the consistency between the simulation and experimental findings lend support to the validity of the MD simulations and the mechanistic model.

We appreciate the reviewer's suggestion to extend the analysis of the gate opening times and probabilities obtained from the simulations. The salient results from the simulations of the point mutants are briefly discussed in the main text and an expanded discussion of these results in the extended data.

Finally, we appreciate the potential confusion arising from separating the MD simulation results and the analysis approach used to extract the gate opening probabilities from the MD simulation results. This was dictated by our understanding of the formatting guidelines but we will enquire with the editor if we can make this formatting change to improve the clarity and flow of the manuscript.

A minor point - there is a typo on pg8 line 19 - "trough" should read "through".
We thank the referee for pointing this error and have corrected this in the revised manuscript.

Reviewer #4 (Remarks to the Author):

The manuscript of Seol and co-workers presents data to show that magnesium regulates conformational changes in type IA topoisomerases. The observation that magnesium is involved in conformational changes Is not new, it has been reported before (see below). The identification of a cluster of amino acids that are involved in conformational changes is interesting, but direct binding of magnesium to some of the amino acids is not shown, weakening the argument that they represent a secondary magnesium binding site. Molecular Dynamics (MD) simulations are used to support the role of magnesium and try to provide an atomistic explanation, but the use of a large force (50 pN) to guide the movements casts doubts on the relevance of the calculations. Overall, the combined single molecule experiments and biochemical assays serve to confirm what had already been observed, that magnesium affects domain movements in type IA topoisomerases. The putative identification of a secondary magnesium binding site is indirect and not completely convincing. Given this, it is not clear that the work adds significantly new information to our understanding of the mechanism of type IA topoisomerases. It represents incremental and derivative work to what was known before.

We first apologize for omitting the Gunn et al. study in the reference.

We agree with the referee that, as stated in the introduction, conformational changes induced by magnesium in topoisomerase IA have been suggested in multiple studies,[7-9] including the Gunn et al work. However, none of these prior studies showed that the conformational changes in the presence of magnesium directly and unambiguously involve gate domain opening or dynamics. Moreover, the lack of gate dynamics in the absence of magnesium is frequently ascribed to a lack of ssDNA cleavage.

We thank the referee for providing comments and constructive criticism, however we respectfully disagree with the characterization of the results by the reviewer, and the broad claims that the work are "incremental and derivative work to what was known before". Furthermore, the reviewer's assertion that " ...the combined single molecule experiments and biochemical assays serve to

confirm what had already been observed, that magnesium affects domain movements in type IA topoisomerases" misconstrues the findings of this work and previous findings.

The vast majority of the literature discussing the roles of magnesium in the catalytic cycle of type IA topoisomerases has focused on catalytic activities mediated by Mg as a cofactor bound to the canonical TOPRIM domain shared by type IA and type II topoisomerases. Although magnesium dependent conformational changes in *E. coli* topoisomerase I have been observed[9-11], no previous work has been able to unambiguously observe that gate opening requires magnesium and gate kinetics depend on magnesium concentration with a similar, and unusual, parabolic response exhibiting a clear maximum. These observations bring an entirely new level of clarity and mechanistic understanding to the magnesium-dependent conformational dynamics of type 1A topoisomerases. For example, the parabolic dependence of topo IA activity on magnesium concentration was previously observed[9,12], but the mechanistic underpinning of this unusual Mg-dependent activity has not previously been elucidated. The Mg-dependent gate kinetics data we obtained provides the mechanistic basis for this longstanding but poorly understood aspect of topo IA activity. No previous work has related the known magnesium-dependence of activity to the Mg-dependent conformational kinetics of the enzyme.

Although the reviewer is correct that we do not directly demonstrate magnesium binding to the acidic triad of residues identified as the putative novel Mg binding site, we present multiple lines of evidence supporting the hypothesis that Mg binding at this site gives rise to the Mg-dependent conformational dynamics: (1) The algorithm BioMetall assigned a high confidence score to the putative Mg-binding site. We have independently confirmed Mg binding to the acidic residues in the putative Mg-binding site with the molecular docking program AutoDock (data included in the revised manuscript). (2) Point mutants designed to specifically disrupt magnesium binding at this site (2E2Q), and designed to disrupt the intra-domain salt-bridge that competes with magnesium binding at this site (R447G), both result in changes in observed Mg-dependent gate dynamics and enzyme activity that strongly support the predictions of the model based on Mg binding at the acidic triad and the competition for the acidic residues by the basic residues associated with gate opening and closing. (3) We demonstrate similar Mg-dependent gate opening and closing in ectopo 1 and identify a functionally conserved acidic acid triad Mg-binding site and competing basic residues in ectopo1, supported by MD simulations. (4) The novel Mg binding site identified by the binding site prediction algorithm, and the competing inter- and intra- domain basic residues are highly conserved at the sequence level among bacterial topoisomerase III enzymes, functionally conserved among bacterial topoisomerase I enzymes, and plausibly functionally conserved in eukaryotic topoisomerase IIIA – RMI1-RMI2 complexes. Furthermore, in the revised manuscript we provide additional data demonstrating that (5) the gate conformational dynamics require a divalent metal cation rather than simply high ionic concentrations, and (6) that different divalent metal ions (Mn and Ca) show strikingly different effects on gate kinetics, indicating that gate conformational dynamics is differently tuned by the different divalent metal ions. Taken together the data provide extremely strong support for the identified acidic triad representing a novel divalent metal binding site that impacts gate dynamics and, importantly, enzymatic activity.

The reviewer is correct that the MD simulations were performed, by necessity, at higher forces than the experimental forces. However, as mentioned in the manuscript, the primary goal of these simulations was to serve as an intermediate step to gain insight into possible mechanistic explanations for the experimentally observed magnesium-dependent gate dynamics. The simulations were not intended to determine the energetics of gate opening or to define the precise transition path; rather, they provide hypotheses that should be further tested experimentally.

Accordingly, these MD results, in conjunction with the prediction of the Mg binding site, allowed us to hypothesize a mechanistic model for gate opening that we tested through mutagenesis and additional MD simulations. The experimental measurements of the gate kinetics of the mutant enzymes are in very good agreement with the predictions of the model. The self-consistency of the simulation results with the experimental results, in particular the results of the mutants that disrupt specific interactions identified from the MD simulations, suggests that the MD simulations have captured key aspects of the gate opening pathways, even if the forces in the simulations were higher than the experimental forces.

Some specific comments.

1. Gunn et al. (Nat Struct Mol Biol. 2017 24(5):484-490. 2017) showed before that domain movement or gate dynamics in type IA topoisomerases is affected by magnesium. In the absence of magnesium, no domain movement was observed (see Figure 4). The authors at the very least should refer to this work and acknowledge that there was previous evidence that magnesium affects conformational changes. Whether magnesium affects directly the protein conformation or indirectly by affecting the interactions with DNA cannot be ascertained from either set of experiments.

We thank the referee for pointing out the serious omission of the study by Gunn et al in the manuscript. This is seminal work in the topo IA field that was the first single-molecule study to directly observe *ec*topo1 conformational changes influenced by magnesium coupled to supercoil relaxation. We were astounded to find that it was not part of the reference list. We suspect that we inadvertently dropped the citation during multiple rounds of revisions. We apologize for omitting this important study and have corrected this oversight in the revised manuscript.

The study by Gunn et al is significant in advancing our understanding of topoisomerase IA by correlating conformational changes with enzyme function. The conformational change were assumed from the fluctuating PIFE (protein induced fluorescence enhancement) signal associated with changes in the local environment of the fluorophore incorporated into the DNA substrate. Although this innovative approach provided direct correlation between ectopo1 conformational change and enzyme activity, it could not unambiguously report on the identity of the dynamic domains, the extent of their motion, or which step or steps in the catalytic cycle were associated with the conformational changes. In particular, the PIFE signal indicated a decrease in the separation between the fluorophore and a protein domain, which resulted in a proposed model that

did not include gate opening, but rather an alternative domain rearrangement associated with catalytic activity (Gunn et al, Figure 5).

However, the reviewer is incorrect in asserting that our approach of directly measuring gate opening cannot distinguish whether magnesium directly affects the protein conformation or indirectly by affecting the interactions with DNA. In contrast to the Gunn et al study where the signal relied on changes in the interaction of the protein with a fluorophore attached to the DNA, the current study directly measures the gate opening dynamics that unambiguously indicates that the gate dynamics are influenced by magnesium. Furthermore, the point mutants significantly alter the Mg-dependence of the gate dynamics, which is inconsistent with the contention that the Mg-dependent gate dynamics we observe arise from Mg-dependent protein-DNA interactions.

Addition made to the main text (page3_line4):

"Magnesium has been implicated in domain conformational changes, including those of the gate domain in *E. coli* topoisomerase 1[9-11]; however, there has been neither a direct observation nor a mechanistic basis established for magnesium-dependent protein-gate motion and subsequent enzymatic activity."

2. The gate dynamics observed are also similar to what Gunn et al reported, with the difference that experiments were done at very different forces. Gunn et al used ~1 pN forces, while Seol et al use forces above 5 pN, which is likely to affect the time the gate remains open. The gate dynamics reported are again similar to what was already reported.

We respectfully disagree with the referee that the gate dynamics measurement in this study are comparable to those reported in the Gunn et al study. In this work, we directly measured the enzyme conformation changes, obtained the gate kinetics, both opening and closing rates, and how both rates depended on magnesium concentration. The fluctuation kinetics measured in the Gunn et al work corresponded to an average time between spikes in the PIFE signal measured at a single magnesium concentration. Despite the elegance of the approach and the important insights provided by the Gunn work, it is simply not possible to claim that the fluctuations reported in that work correspond to the gate dynamics that we observe. There is too much ambiguity and uncertainty in the origins of the fluctuations reported in the Gunn et al work to assign them unambiguously to the gate kinetics we measured. Moreover, even if the kinetics in both measurements are associated with gate dynamics, the current study has identified novel magnesium-dependent kinetics of both gate opening and closing, and we elucidate a novel mechanistic model, verified through mutagenesis and simulations, which reveals an unexpected but conserved magnesium binding site, distant from the active site, that controls gate kinetics.

3. The identification of a second magnesium binding site is based on MD calculations and sequence analysis. Given the number of sequences available nowadays, it would be more compelling to see a larger sequence alignment with some quantitation of the presence of charged

amino acids in the region. Not just a few sequences that agree with their hypothesis, but a much larger analysis that helps understand how widespread these charged regions are.

We agree with the reviewer that demonstrating the conservation of the putative magnesium binding site and the overlapping salt-bridge network provides important confirmation and generalization of the proposed model. It is important to point out that the motif we identified, and metal binding motifs more generally, tend to be more structurally conserved rather than strictly conserved at the sequence level [13]. Nonetheless, we demonstrate extremely high levels of sequence conservation for representative bacterial topoisomerase 3 sequences spanning a wide range of sequence conservation (85.76% to 30.21% identity). Furthermore, the putative Mg binding site that we identify was previously recognized as a highly conserved acidic patch, with an unknown function, in domains II and III among prokaryotic topoisomerase IA[14]. Taken together, these existing and new findings demonstrate the high degree of conservation among all topoisomerase 3 enzymes.

In light of the reviewer's comments, we have expanded the sequence alignments to 470 bacterial topo 3 enzymes, the sequency identities of which range from 23% to 99.8% compared to ectopo3.

For bacterial topoisomerase I, we have extended the alignments to over 1000 sequences in the revised extended data. Overall, we find remarkably high levels of sequence conservation across a wide range of bacterial topoisomerase IA enzymes. These findings lend support to the hypothesis that the magnesium binding site and overlapping salt-bridge network represent a highly conserved functional motif in bacterial topoisomerase IA enzymes.

4. Binding of magnesium to the acidic residues in the putative secondary site is implied, not demonstrated. The proposed interaction is basically a charge interaction that will be affected by the presence of cations and may not necessitate direct binding. The biochemical and mutagenesis experiments support a role for the identified amino acids, but not a direct role in binding magnesium. This should be made much clearer in the manuscript. Also, it is not clear that magnesium Is the only divalent cation that would support the proposed mechanism, which could be the case if it is simply an electrostatic effect. Experiments with other divalent cations may help in this regard.

We thank the reviewer for raising this point. The reviewer is right that the current study did not directly show magnesium binding to the putative binding site, which is beyond our expertise.

Nevertheless, we performed a series of tests of the proposal, all of which support the existence of a divalent metal binding site that overlaps with competing salt-bridges to alter gate kinetics in a magnesium-dependent manner. In particular, the 2E2Q mutation, which removes two of the three acidic residues comprising the putative divalent metal binding site, exhibits gate dynamics as well as gate opening even in the absence of magnesium – thus bypassing the magnesium requirement. This result confirms our hypothesis that the interdomain salt-bridge formation via these two acidic residues acts as a key barrier to the gate opening transition, and that magnesium binding is

necessary to disrupt this interdomain salt-bridge (Fig. 4E). Together the extensive measurements, modeling, and simulations support the existence of a divalent metal binding site. Nonetheless, we have taken the reviewer's suggestions to make it clear in the revised manuscript that we infer, but do not directly observe, magnesium binding to the putative magnesium binding site.

The reviewer raised two valid questions: first, if the effect of magnesium could be non-specific (electrostatic screening) and potentially reproduced by any cation and secondly, if it is specific, could other divalent metal ions play similar roles? To address the first question, we performed gate opening measurements of both *ec*topo1 and ectopo3 at high concentrations of monovalent cations (300 mM potassium) in the absence of magnesium. We found that this 3-fold higher concentration of monovalent cations did not support gate opening and inhibited ectopo IA enzymatic activity, without reducing DNA binding. These results indicate that a specific divalent cation interaction with the residues is required to support gate opening and subsequent activity of ectopo IA enzymes.

To address the second question, we demonstrated that $MnCl_2$ and $CaCl_2$ have different effects on gate conformational dynamics and supercoil relaxation compared to magnesium. These results highlight that though other divalent metal ions can functionally replace Mg as the catalytic cofactor, they cannot reproduce magnesium specific interactions with the amino acid residues and subsequent gate conformational dynamics.

Change made to the main text

Original text

"Collectively, these results demonstrate that Mg^{2+} facilitates TopIA gate opening."

Revised text (page5_line6):

"Gate opening was not observed with high concentrations of monovalent salts (300 mM potassium) in the absence of magnesium (Extended Data Fig. 3C-F), suggesting that TopIA gate opening is specifically facilitated by Mg^{2+} rather than through nonspecific electrostatic screening."

Original text

"Overall, these results establish that Mg^{2+} is indispensable not only for gate opening but also for optimal conformational dynamics of the gate."

Revised text (page5_line25):

"Overall, these results establish that Mg^{2+} is indispensable not only for gate opening but, more importantly, for optimal gate conformational dynamics. To probe the specificity of Mg^{2+} effects on gate dynamics, we replaced Mg^{2+} with divalent metal ions that can support enzymatic activity; manganese (Mn^{2+}) and Calcium (Ca^{2+})[15]. No gate dynamics was observed with *ec*topo3 in the presence of Ca^{2+}, despite robust ssDNA cleavage (Extended Data Fig. 6). Consistent with the absence of gate dynamics, *ec*topo3 supercoil relaxation activity was inefficient with Ca^{2+}, underscoring the coupling between gate dynamics and enzymatic activity (Extended Data Fig. 6). Whereas Mn^{2+} supported *ec*topo3 gate dynamics, the dynamics were strikingly different from those

with Mg^{2+} (Extended Data Fig. 6). Furthermore, the absence of gate dynamics at 75 µM; a 7.5-fold higher concentration of Mn^{2+} than estimated *in vivo* (~10 µM)[16] suggests that is unlikely that Mn^{2+} would replace Mg^{2+} *in vivo*."

5. I am skeptical about the MD simulations as they are done at very high forces. Given the geometry selected for the simulations and the forces used, they are almost certain to show a large opening in a pre-determined direction. Some of the single molecule experiments to show gate opening are also done at very high forces (16 pN or larger), which again will bias the opening. At the very least, the implications of doing experiments and simulations at forces that clearly overcome thermal fluctuations (the likely source of domain movement in type IA topoisomerases) should be discussed.

We appreciate and share the reviewer's concerns with the use of high forces along a predetermined direction. However, these conditions may not be substantially different from the physiological environment for topoisomerase IA, as the activities of polymerases and translocases can generate tens of piconewtons of force on DNA[17,18]. In line with this, we have previously demonstrated that ectopo 1 can relax negative supercoils at forces in excess of 12 pN[1]. With respect to the predetermined force direction, the simulations are specifically designed to mimic the experimental pulling conditions. The use of 50 pN in the MD simulations was necessary to observe gate opening transitions within a reasonable computational timeframe. We acknowledge that such high forces could bias the transition pathway and the energetics of gate opening. Therefore, we used MD simulations as an intermediate step to gain mechanistic insight into how magnesium might influence gate conformational dynamics, and to generate hypotheses for subsequent experimental testing, rather than to obtain precise gate kinetics. Importantly, despite the use of 50 pN in simulations, the extent of gate opening observed in MD was comparable to the measured values under similar, though lower forces. Moreover, the behavior of the mutants (2E2Q and R447G) was consistent with our hypothesis, confirming the relevance of the simulations to our experimental findings. Overall, the MD simulation results provided valuable molecular insights, which we subsequently verified through mutagenesis, additional modeling approaches, and further MD simulations of the specific mutants. Nonetheless, we took the reviewer's suggestion and expanded the discussion of this point the revised manuscript.

Although some of the gate-opening experiments were performed at forces greater than 16 pN, the gate kinetics were measured at ~5 pN. The higher force (above 16 pN) experiments were performed on the hairpin substrate, which requires at least 15 pN to open. The reviewer is correct that these high forces may increase the opening probability, and absolutely prevent closing of the gate. However, these experiments were specifically designed to test gate opening events, not the extent or kinetics of gate opening. The high forces in these experiments prevented closure of the gate, simplifying the analysis. For example, high force hairpin pulling experiments provided a stringent test to determine whether gate opening occurred in the absence of magnesium within the given time frame (30 min).

Addition made to the main text (page6_line26):

“ Although simulations at such high-forces are not ideal for obtaining experimentally relevant kinetics or an accurate transition path, they can provide valuable molecular insights into how the residues of interest influence the gate opening transition.”

6. The free magnesium concentration in E. coli is in the very low mM range. It is thus not surprising that the enzymes are optimal at around 3 mM. This should be mentioned.

We thank the referee for pointing this issue. In fact, the gate kinetics as well as ensemble enzymatic activity measurements showed that the optimal Mg concentration peaked at 1 mM, which is comparable to the free Mg^{2+} concentration (1-2 mM) in *E coli* and other cell types.[16,19]

1 Mills, M., Tse-Dinh, Y.-C. & Neuman, K. C. Direct observation of topoisomerase IA gate dynamics. *Nature structural & molecular biology* **25**, 1111-1118 (2018). https://doi.org/10.1038/s41594-018-0158-x

2 Zhang, H. L., Malpure, S. & DiGate, R. J. Escherichia coli DNA Topoisomerase III Is a Site-specific DNA Binding Protein That Binds Asymmetrically to Its Cleavage Site. *Journal of Biological Chemistry* **270**, 23700-23705 (1995). https://doi.org/10.1074/jbc.270.40.23700

3 Zhang, Z., Cheng, B. & Tse-Dinh, Y.-C. Crystal structure of a covalent intermediate in DNA cleavage and rejoining by Escherichia coli DNA topoisomerase I. *Proceedings of the National Academy of Sciences of the United States of America* **108**, 6939-6944 (2011). https://doi.org/10.1073/pnas.1100300108

4 Changela, A., DiGate, R. J. & Mondragón, A. Structural Studies of E. coli Topoisomerase III-DNA Complexes Reveal A Novel Type IA Topoisomerase-DNA Conformational Intermediate. *Journal of molecular biology* **368**, 105-118 (2007). https://doi.org/10.1016/j.jmb.2007.01.065

5 Humphrey, W., Dalke, A. & Schulten, K. VMD: Visual molecular dynamics. *Journal of Molecular Graphics* **14**, 33-38 (1996). https://doi.org/https://doi.org/10.1016/0263-7855(96)00018-5

6 Bakan, A., Meireles, L. M. & Bahar, I. ProDy: Protein Dynamics Inferred from Theory and Experiments. *Bioinformatics* **27**, 1575-1577 (2011). https://doi.org/10.1093/bioinformatics/btr168

7 Zhu, C.-X., Roche, C. J., Papanicolaou, N., DiPietrantonio, A. & Tse-Dinh, Y.-C. Site-directed Mutagenesis of Conserved Aspartates, Glutamates and Arginines in the Active Site Region of Escherichia coli DNA Topoisomerase I *. *Journal of Biological Chemistry* **273**, 8783-8789 (1998). https://doi.org/10.1074/jbc.273.15.8783

8 Zhu, C.-X., Roche, C. J. & Tse-Dinh, Y.-C. Effect of Mg(II) Binding on the Structure and Activity ofEscherichia coli DNA Topoisomerase I. *Journal of Biological Chemistry* **272**, 16206-16210 (1997). https://doi.org/10.1074/jbc.272.26.16206

9 Zhu, C.-X. & Tse-Dinh, Y.-C. The Acidic Triad Conserved in Type IA DNA Topoisomerases Is Required for Binding of Mg(II) and Subsequent Conformational Change. *Journal of Biological Chemistry* **275**, 5318-5322 (2000). https://doi.org/10.1074/jbc.275.8.5318

10 Gunn, K. H., Marko, J. F. & Mondragón, A. An orthogonal single-molecule experiment reveals multiple-attempt dynamics of type IA topoisomerases. *Nature Structural &Amp; Molecular Biology* **24**, 484 (2017). https://doi.org/10.1038/nsmb.3401

https://www.nature.com/articles/nsmb.3401#supplementary-information

11 Sissi, C. & Palumbo, M. Effects of magnesium and related divalent metal ions in topoisomerase structure and function. *Nucleic acids research* **37**, 702-711 (2009). https://doi.org/10.1093/nar/gkp024

12 Narula, G. *et al.* The Strictly Conserved Arg-321 Residue in the Active Site of Escherichia coli Topoisomerase I Plays a Critical Role in DNA Rejoining *. *Journal of Biological Chemistry* **286**, 18673-18680 (2011). https://doi.org/10.1074/jbc.M111.229450

13 Dudev, M. & Lim, C. Discovering structural motifs using a structural alphabet: Application to magnesium-binding sites. *BMC Bioinformatics* **8**, 106 (2007). https://doi.org/10.1186/1471-2105-8-106

14 Duguet, M., Serre, M.-C. & Bouthier de La Tour, C. A Universal Type IA Topoisomerase Fold. *Journal of Molecular Biology* **359**, 805-812 (2006). https://doi.org/https://doi.org/10.1016/j.jmb.2006.04.021

15 Sissi, C., Cheng, B., Lombardo, V., Tse-Dinh, Y.-C. & Palumbo, M. Metal ion and inter-domain interactions as functional networks in E. coli topoisomerase I. *Gene* **524**, 253-260 (2013). https://doi.org/https://doi.org/10.1016/j.gene.2013.04.008

16 Theillet, F.-X. *et al.* Physicochemical Properties of Cells and Their Effects on Intrinsically Disordered Proteins (IDPs). *Chemical Reviews* **114**, 6661-6714 (2014). https://doi.org/10.1021/cr400695p

17 Halma, M. T. J. & Xu, L. Life under tension: the relevance of force on biological polymers. *Biophysics Reports* **10**, 48-56 (2024). https://doi.org/10.52601/bpr.2023.230019

18 Hill, H. J. & Golic, K. G. Chromosome Tug of War: Dicentric Chromosomes and the Centromere Strength Hypothesis. *Cells* **11**, 3550 (2022).

19 Tyrrell, J., McGinnis, J. L., Weeks, K. M. & Pielak, G. J. The Cellular Environment Stabilizes Adenine Riboswitch RNA Structure. *Biochemistry* **52**, 8777-8785 (2013). https://doi.org/10.1021/bi401207q

REVIEWERS' COMMENTS

We sincerely thank all the reviewers for their careful evaluation of our manuscript and for their constructive feedback, which has greatly strengthened our work.

Reviewer #1 (Remarks to the Author):

All comments have been adequately addressed. The revised manuscript substantially clarifies the single-molecule methods and interpretation. This work represents a valuable contribution to understanding magnesium's role in type IA topoisomerases.

Reviewer #2 (Remarks to the Author):

I co-reviewed this manuscript with one of the reviewers who provided the listed reports. This is part of the Nature Communications initiative to facilitate training in peer review and to provide appropriate recognition for Early Career Researchers who co-review manuscripts.

Reviewer #3 (Remarks to the Author):

The authors have made substantial efforts to address the referees' comments, and those raised by me have been addressed satisfactorily.

Reviewer #4 (Remarks to the Author):

The revised manuscript of Seol et al. addresses adequately the concerns of the reviewers. The addition of data in the presence of other divalent cations does help strengthen the argument in favor of magnesium specificity.
I think that the manuscript would benefit from another round of careful editing as there are still some statements that are not clear or misleading. For example:
Page 6, line 25 and subsequent. The authors first state that " Although simulations at such high forces are not ideal for obtaining experimentally relevant kinetics or an accurate transition path …*" In the next sentence they state "The SMD results revealed that gate opening proceeds via two distinct transition pathways under …" If the simulations are not adequate to obtain a transition pathway, why mention next that a transition pathway was obtained. It seems contradictory or misleading. Clarification is needed.*

Overall, the revised manuscript is stronger and the authors make a much better case for the role of magnesium in conformational changes.

We apologize that our statement did not fully convey our intention to the reviewer.

As the reviewer had correctly pointed out in the previous review, the effect of high force would smooth out the rugged energy landscape by reducing the level of fluctuation. Thus, the detailed energy landscape and corresponding transition path at high force may not be exactly same as those at no or lower force. However we understand why it could sound inconsistent with the following sentence as the reviewer pointed out. We revised the sentence to clarify this issue.

Original sentence

" Although simulations at such high forces are not ideal for obtaining experimentally relevant kinetics or an accurate transition path …"

Revised sentence

"Although simulations at such high-forces are not ideal for obtaining experimentally relevant kinetics or the exact energy landscape, they can still reveal the qualitative structural mechanisms, identify accessible intermediates and distinct conformational pathways[42]. Additionally, they can provide valuable molecular insights into how the residues of interest influence the gate opening transition. The SMD results revealed that gate opening proceeds via two distinct conformational pathways under identical simulation conditions:.."
